# DEprescribing: Perceptions of PAtients living with advanced cancer. A multicentre, prospective mixed observational study protocol

**DOI:** 10.1371/journal.pone.0305737

**Published:** 2024-08-20

**Authors:** Adrien Evin, Marianne Bourdon, Pierre Nizet, Jean-Benoit Hardouin, Caroline Victorri-Vigneau, Jean-François Huon

**Affiliations:** 1 Service de Soins Palliatifs et de Support, CHU de Nantes, Nantes Université, Nantes, France; 2 INSERM, Methods in Patients-centered Outcomes and Health Research, SPHERE, CHU Tours, CHU Nantes, Tours Université, Nantes Université, Nantes, France; 3 Integrative Center for Oncology, Angers, Nantes, France; 4 Pharmacie, CHU de Nantes, Nantes Université, Nantes, France; 5 Direction de la Recherche et de l’Innovation, Plateforme de Méthodologie et Biostatistique Unit, CHU de Nantes, Nantes Université, Nantes, France; 6 Centre d’évaluation et d’information sur la Pharmacodépendance-addictovigilance, CHU de Nantes, Nantes Université, Nantes, France; PLOS: Public Library of Science, UNITED KINGDOM OF GREAT BRITAIN AND NORTHERN IRELAND

## Abstract

**Introduction:**

Polypharmacy in patients with advanced cancer represents a major public health problem, leading to risk of iatrogenesis, decrease of quality of life and increase of healthcare costs. In the field of geriatrics, health policies have been developed to address polypharmacy through the use of deprescribing tools. Recently, palliative care initiatives have been introduced, yet these have not fully considered the specificities of this population, particularly their perceptions. It is therefore important to better understand patients’ perceptions of deprescribing in order to adapt tools and actions to make these approaches more effective.

**Objectives:**

The aim is to investigate patients’ perceptions of deprescribing in palliative oncology care, and to explore factors that may influence patients’ attitudes and beliefs about deprescribing and to validate a specific questionnaire (rPATD) in this population. An ancillary study will investigate the relationship between patients’ health literacy and their perception of deprescribing.

**Method:**

A prospective, observational, multicenter study will be conducted using a sequential mixed exploratory design in a population of patients living with advanced cancer and with a physician-estimated life expectancy of less than 1 year. The study will include an initial qualitative phase. Individual semi-structured interviews using a descriptive approach (thematic analysis) will be conducted (upon saturation). Following analysis of the qualitative data, a quantitative study including 300 patients will be realized to meet secondary objectives. Several data will be collected and 2 self-questionnaires will be administered: the BMQ (beliefs about medicine) and rPATD (perception of deprescribing) possibly supplemented by additional items if required by the qualitative analysis. The auxiliary study will be conducted during this second phase, using a validated self-questionnaire to assess patients’ level of literacy.

**Conclusion:**

The disparate outcomes will facilitate the understanding of the perception of deprescribing in palliative oncology care, enabling the development of tailored approaches adapted to this population.

**Trial registration:**

**ClinicalTrials Identifier**: NCT06193083.

## Introduction

### Polymedication in palliative care

Polymedication is defined by the World Health Organisation as the administration of several drugs at the same time or the administration of an excessive number of drugs [1]. It is a common phenomenon in patients suffering from serious, life-threatening and incurable illnesses [2, 3], with the average number of prescribed medications per patient ranging from 3 to 23 [3]. However, polymedication can be associated with reduced quality of life and an increased risk of iatrogenicity [2, 4], which increasingly leads clinicians to limit drugs to those that are necessary for the patient. Some prescribed drugs can be considered as inappropriate for patients (PIMs—Potentially Inappropriate Medications) due to their age, state of health or co-morbidities. This may result in adverse effects or potentially dangerous drug interactions. In international studies conducted in palliative care, the prevalence of patients with at least one potentially inappropriate prescription is reported to range from 15% to 92% [3].

### Deprescribing and palliative care

Deprescribing is currently a crucial issue in medicine. It is defined as the process of reducing or stopping the use of a drug or drug treatment because of its ineffectiveness, inappropriateness, adverse effects, dangerous drug interactions or according to patient preference. This approach also aims to optimize drug therapy by reducing the risks and costs associated with unnecessary or inappropriate drug treatments [2, 5].

To date, international deprescribing policy has focused mainly on the elderly. However, in recent years there has been debate about adapting this policy to the palliative care population [4, 6], especially for advanced cancer patients, who are at higher risk of multiple drug interactions [4, 7, 8], especially with cancer-specific treatments [9]. Deprescribing in palliative oncology care requires individualized care, since uncontrolled symptoms and adverse effects can appear after stopping treatments [10]. Careful planning and clear communication between healthcare professionals, patients and caregivers are needed to promote effective, safe and accepted deprescribing [11].

In light of these concerns, tools have been developed to assist physicians in the process of deprescribing [12]. For instance, the STOPPFrail 2 tool [13] (in S1 File) aims to identify whether a patient is taking PIMs. It has been validated in a geriatric population with an estimated life expectancy of less than one year, and is not specific to oncology. In the context of oncology palliative care, the OncPal tool has been developed to identify PIMS in patients with a life expectancy of less than 6 months [14]. To our knowledge, no tool has been developed to identify PIMs in cancer patients with more than 6 months of life expectancy. In addition, there is a lack of tools to assess how deprescribing is perceived by cancer patients, while the rPATD tool assesses patients’ beliefs and attitudes regarding deprescribing in the geriatric population [15].

Few studies have examined the perceptions of deprescribing among cancer patients [16, 17]. Results from a study on the cessation of statins [17] suggest that palliative care patients may not be concerned about the consequences of stopping their preventive treatments. However, for some patients, it can be challenging to question the discontinuation of a “lifelong” treatment, as it may cast doubt on the rest of their lives. They may interpret it as an imminent death.

Physicians themselves may be reluctant to suggest treatment discontinuation and its consequences for patients [18]. Consequently, further research in this area is required to better support deprescribing in palliative care, which is an integral part of care and can help to improve patients’ quality of life [10]. It seems necessary to better understand patients’ perceptions of deprescribing, in particular for potentially inappropriate drugs, in order to adapt support to each patient. Indeed, we believe that these elements can be influenced by the health literacy of our patients, defined as the ability to acquire, understand and use information in ways that promote and maintain good health [19]. To date, and to the best of our knowledge, no study has yet investigated this hypothesis.

The main aim of our study will be to describe patients’ attitudes and beliefs towards deprescribing, using a patient-centered approach. As secondary objectives, the factors that may influence patients’ attitudes and beliefs regarding deprescribing will be studied, and the rPATD will be adapted and validated in this population. Finally, the relationship between patients’ level of health literacy and their perception of deprescribing will be examined.

## Methods

### Study design

A prospective, observational, multicenter study will be conducted using a sequential mixed exploratory design in a population of patients living with advanced cancer and with a physician-estimated life expectancy of less than 1 year.

The study will include an initial qualitative phase concerning patients’ attitudes and beliefs towards deprescribing. Individual semi-structured interviews using a descriptive approach will be conducted. Following the analysis of the qualitative data, a quantitative study will be conducted to understand the factors that may influence patients’ attitudes and beliefs regarding deprescribing. This will be achieved through the use of questionnaires that explore two keys areas: *(I)* patients’ representations of medicine (BMQ) and *(II)* patient’s perception of deprescribing. The rPATD questionnaire may be adapted depending on the results of the qualitative study. The psychometric properties of the rPATD in this population will then be studied to validate this tool.

During this second phase, an ancillary study will be conducted based on voluntary patient participation to investigate the relationship between the patients’ level of health literacy and their perception of deprescribing.

### Study setting and scientific committee

This multicenter study will be conducted in oncology and/or palliative care departments of hospitals located in western France. In this geographical area, all 5 oncology/ palliative care centers are participating.

The scientific committee of this study is a multidisciplinary team of clinicians and researchers, comprising pharmacists, pharmacologists, physicians, biostatisticians and psychologists. They are interested in the patient-centered approach and the proper use of medication. They supervise all phases of the studies, from methodological choices to the analysis and interpretation of results. For each phase (qualitative and quantitative), there are experts in each methodology.

### Population

The study will be introduced to adult patients with advanced "solid" cancer who have at least one PIM, followed either as inpatients or outpatients, and whose life expectancy is estimated by the physician to be less than one year.

### Inclusion criteria

- Patients over 18 years old
- Locally advanced or metastatic solid cancer (i.e., palliative care as defined by the World Health Organization [20])
- Life expectancy estimated by the physician at inclusion of less than 1 year (will be estimated by the attending physician’s surprise question:"Would you be surprised if your patient died within a year?" [21–23]).
- Hospitalised (whether in conventional hospitalization or in a day hospital) or in consultation
- With at least one PIM (identified using STOPPfrail 2) [13] (in S1 File). This is a tool developed in geriatrics, validated for a population with an estimated life expectancy of less than one year, and is not specific to oncology.
- Patient not having expressed opposition to participating in the study after receiving information from the physician.

And for patients in the qualitative study:

- Patient having signed the authorization to have their voice recorded during the semi-structured interview for written transcription.

### Non-inclusion criteria

- Major under guardianship, protected person
- Patient unable to speak or write French
- Patients with impaired judgment, cognitive or sensory impairments preventing them from receiving informed information, answering questionnaires or participating in study interviews.

### Data collection and measurement

#### First part of our mixed study: Qualitative phase

This methodology describes patient perceptions without limiting hypotheses in an inductive approach, thus allowing to perceive the specificities of our population and to create themes that have potentially not been developed in the literature [24].

Individual semi-structured interviews will be conducted. An interview guide (in S2 File) will be used to help the interviewer to address the guide themes, based on literature data [17] from studies in other populations and the experience of the scientific committee. The guide will be tested with patients meeting the study’s inclusion criteria. Each patient will be offered a semi-structured face-to-face interview on the same day of inclusion or within 15 days. If, for health reasons or scheduling constraints, a physical meeting cannot take place, the interview will be carried out by videoconference or telephone. These interviews will be digitally double-recorded (2 voice recorders). A researcher’s diary will be kept during all interviews, in order to note information perceived by the researcher but potentially not perceptible on the recordings. This will provide information likely to help interpret the data discussed. There will be only one interview per participant. The average duration of an interview is estimated at 30–45 minutes. The individual interviews will be conducted by two researchers. The main researcher (AE) is a palliative care physician who has received training in qualitative methodology and semi-structured interviews. The second researcher (PN), who will be conducting interviews with patients followed by the aforementioned physician, is a clinical pharmacist also trained in qualitative methods and semi-structured interviews.

A service provider will fully transcribe the interviews. The data will then be subjected to analysis by the main researcher (AE), following the six-stages of reflexive thematic analysis methodology described by Braun and Clarke [25–27], namely: (I) becoming familiar with the dataset, (II) creating code, (III) creating initial themes, (IV) developing and revising themes, (V) refining, defining and naming themes, and (VI) writing a report. In addition, a bottom-up approach [27] and semantic (descriptive) coding will be employed. The dataset will be coded by two researchers, and the scientific committee will be consulted for the creation of themes. NVivo software version 14 will be used to create the codes and the themes.

The sample of interviewees will be diversified according to primary cancer type, patient age and follow-up hospital sites. Recruitment will cease when the saturation of themes is reached, i.e. when the coding of two successive interviews does not substantially modify the themes. Based on samples in qualitative studies using individual research interviews, the recruitment of about 25 patients is planned. It may be increased if data saturation is not reached at 25.

### Second part of our mixed study: Quantitative phase

Once the conclusions of the qualitative study have been drawn (depending on the results of the qualitative study, items not included in the rPATD may be added), a quantitative study will be conducted (including 300 patients).

1. In order to meet our **main objective** (investigating the perception of deprescribing held by patients in palliative cancer care), we will describe the distribution of rPATD scores (and potentially added items not present in rPATD) in our sample. The rPATD is a patient self-questionnaire that has been validated and adapted in French [15] (in S3 File) to assess patients’ perceptions of their treatments and deprescribing. It is available for use without license. The questionnaire comprises 22 questions, which are assessed on a 5-point Likert scale. It includes two questions on overall satisfaction with medication use and willingness to accept deprescribing recommendations, as well as 20 questions grouped into four validated factors: (I) perceived drug burden (Burden factor), (II) attitudes towards the appropriateness of prescribed medications (Relevance factor), (III) concerns about stopping medications (Concerns about stopping medications factor), and (IV) participants’ level of knowledge about their medications and their degree of involvement in the medication decision-making process (Involvement factor). The instrument was initially employed in the geriatric population, but recent studies have utilised it in adult patients of all ages [28–30].
2. In order to meet **secondary objective 1** (studying factors that may influence patients’ attitudes and beliefs regarding de-prescribing), a linear model will be employed to explain the rPATD questionnaire scores by the various covariates collected. These include the sex, age, highest level of education, type of cancer (by major primary site), number of months since cancer onset, number of metastatic sites, performance status [31] at inclusion, presence of systemic cancer treatments (chemotherapy, targeted therapy, immunotherapy, hormone therapy…), presence of follow-up by palliative care team, patient’s place of care (hospitalization, home, nursing home…), number of medications (number of different molecules prescribed and taken by the patient), number of PIMs (using the STOPPfrail 2 tool [13]), the physician’s estimate of the patient’s life expectancy at 3 and 6 months (using the surprise question "Would you be surprised if your patient died within 3 months?") [21–23], person in charge of medication management (patient, caregiver) and patient’s beliefs about treatments in general. For the latter, the Beliefs about Medicine Questionnaire (BMQ) will be utilized. The BMQ is a 18-item self-administered questionnaire, designed to explore patients’ representations of medicine (in S4 File). It is validated in French [32] with license-free use. Ten items concern specific beliefs about prescribed treatments (the need to take a treatment to stay healthy items 1, 3, 4, 7, 10) and fear of treatment-related risks items 2, 5, 6, 8, 9). Furthermore, eight items concern general beliefs (the idea that physicians overuse medicines items 11, 14, 17, 18 and fear of potential danger associated with medication in general items 12, 13, 15, 16). A 5-point Likert-type scale was used for each item, with higher scores indicating stronger beliefs.
3. In order to meet the **secondary objective 2** (validating the rPATD in this population by evaluating the psychometric properties of the rPATD in this population), a confirmatory factor analysis (CFA) will be performed on the rPATD items. The structure of the rPATD will be deemed coherent if the CFI value is greater than 0.9. and the RMSEA value less than 0.08. The reliability will be considered good if Cronbach’s alpha value is greater than 0.7 for each dimension. Concurrent validity will be considered to be good if Spearman correlations between rPATD scores and BMQ questionnaire scores are significant.

#### Ancillary study

In order to meet the **objective of the ancillary study** (investigating the relationship between patients’ level of health literacy and their perception of deprescribing), we will calculate the Spearman correlation coefficients between the rPATD scores and the FCCHL/HLS14 scores. The FCCHL (Functional, Communicative and Critical Health Literacy) / HLS14 (14-item health literacy scale) (in S5 File) is self-administered questionnaire [33], validated in French. It is available without a license and comprises 14 items with a 5-point Likert scale to assess health literacy [34] which can be categorized as functional literacy, communicative or interactive literacy, and critical literacy.

### Ethical considerations and patient information

Each patient will be provided with oral and written information on the study (in S6 File). The physician who included the patients will be required to obtain a written consent. Patients who agree to participate in the qualitative study will be asked to sign an authorization form for voice recording and interview transcription (in S7 File).

The study protocol was approved by the “Groupe Nantais d’Ethique dans le Domaine de la santé” (GNEDS) and registered under the reference number 23-156-12-272. Data processing will be recorded in the Nantes University Hospital’s RGPD register. As such, the data will be pseudo-anonymised with coding that complies with the regulations.

This study has been registered on ClinicalTrials.gov under the identifier NCT06193083.

### Safety

This study is observational, with no direct risks associated with participation. Given the potential fragility of the patients, each of them will be able to benefit from psychological support as part of their follow-up.

### Study timetable

**Fig 1:**
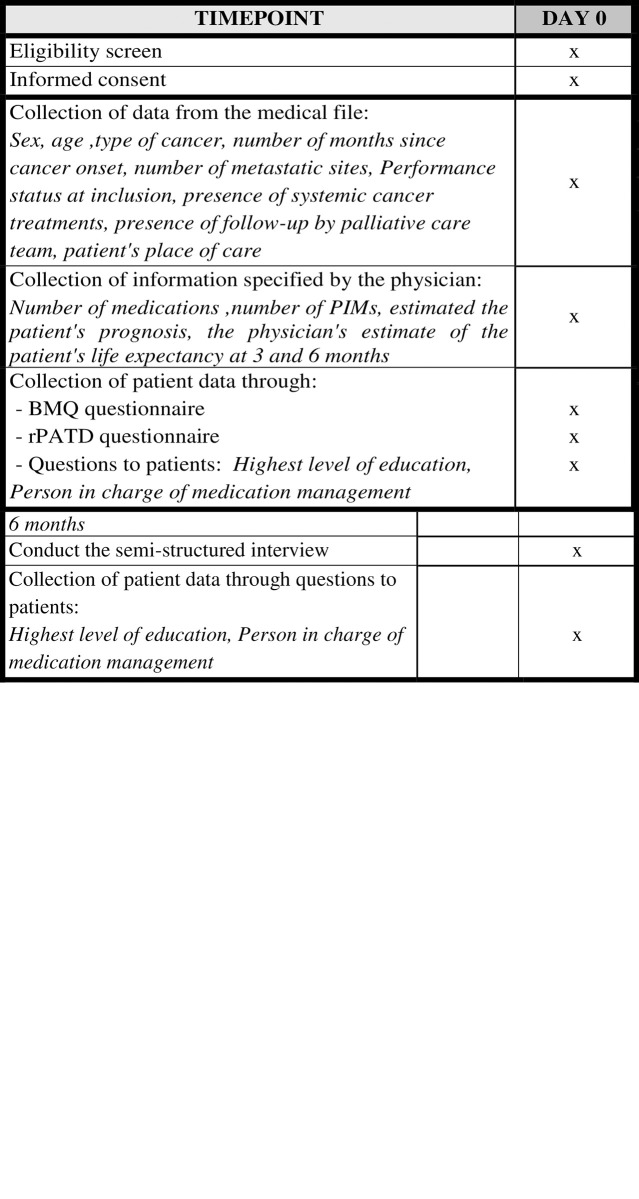
SPIRIT schedule. a. Timepoint for Qualitative Phase. b. Timepoint for Quantitative Phase.

For greater clarity of the timetable and stages of the study, we will provide details by phase of the study (in **Fig 1**). For the qualitative phase, the recruitment period began on 28 February 2024 and will last 8 months, until 28 October 2024. For he quantitative phase, the recruitment period is scheduled to begin around March 1, 2025 and will last 12 months, until March 12, 2026.

## Discussion

To our knowledge and to date, this multicentric study is the first to address the perception of deprescribing in advanced cancer patients. Its mixed-design will enable to describe the differences in perception of this population and the factors that may influence patients’ attitudes and beliefs about deprescribing. This project will also study patients’ health literacy, which is innovative in palliative care, and the link with the perception of deprescribing. This project will link research to care and teaching. In addition, the findings of this project could be used to create deprescribing tools for the various healthcare professionals involved in (de)prescribing. The project is conducted by a multidisciplinary team, which is a major strength of this study. This multidisciplinary team is recognized for its clinical research on the evaluation and study of patient perceptions, particularly in palliative care, and has expertise in a variety of methodologies.

### Strengths, limitations and discussion about the study design

#### Concerning the choice of the population

A purely solid oncology population has been selected, as it would be inappropriate to study perceptions of deprescribing for totally different trajectories of palliative care patients [35, 36]. A life expectancy of less than one year has been chosen because if the patient’s life expectancy seems very short, the question of deprescribing seems less complex. There is no reference prognostic score for assessing the life expectancy of a patient in an advanced oncologic situation. The literature shows that the surprise question is a reliable tool that calls on the physician’s clinical experience [21–23].

The choice of the tool used to identify PIMs (the STOPPfrail 2) is debatable, as there is no specific tool for the study population [12]. Given that the target population has a life expectancy of less than a year, it would appear more appropriate to utilise a tool that has been validated in a different population but with the same life expectancy, rather than one validated in a population of cancer patients with a shorter life expectancy [14]. Indeed, it can be argued that PIMs are not the same according to estimated life expectancy.

#### Concerning the choice of the inclusion centers

With regard to the selection of the inclusion centers, it should be noted that this study is not international in scope. Consequently, the results may not be fully generalisable. However, the inclusion of five centers in this western region allows for the recruitment of inpatients and outpatients, thereby facilitating the recruitment of a diverse population in terms of cancer type, age and living area (rural, urban).

#### Concerning the qualitative methodology

A descriptive approach is employed that aims to "simply describe a phenomenon, situation or event in its context" and describe “people’s personal experiences and responses to an event or situation" [24, 37, 38], which appears appropriate to the objectives.

In terms of the robustness of the results, the interviews will be conducted by two researchers, the dataset will be coded also by two researchers, and the scientific committee will be consulted for the creation of themes. Given the objectives of the study (perception of a patient, not a group) and the specificities of the population, focus groups do not appear to be an appropriate data collection method in this case. Furthermore, they do not seem to be an appropriate method for triangulation of methods.

#### Concerning the choice of questionnaires: BMQ, rPATD and FCCHL /HLS14

As there is no validated questionnaire for this specific population, questionnaires that are frequently used in the literature, validated in French and with license-free use were chosen. The rPTAD is a reference questionnaire to study the perception of deprescribing by patients. Data are available on this tool in large populations, which will enable the results of this study to be compared with those in the literature [39]. The BMQ is a questionnaire regularly used in psychometric validation studies for the translation of the rPTAD [40]. It was also utilised for psychometric validation during the construction of the initial rPATD [41]. Finally, for the FCCHL /HLS14, it was preferable to use a questionnaire that had already been validated in French, which was short and easy for patients to use.

#### Concerning the choice of an ancillary study

As previously stated, we have deliberately chosen to leave patients free to participate in this complementary study, to avoid the principal study being perceived as too burdensome by our often tired patients. This is why we refer to it as an auxiliary study, given that the questionnaire is not systematic. This choice introduces a population selection bias, but the risk of the study appearing too burdensome seems to us to outweigh this bias. The objective is simply to obtain preliminary results that will provide information for future studies on the link between the level of health literacy and patients’ understanding of the phenomenon of deprescribing.

#### Concerning our sample size

With regard to the qualitative part, the saturation of themes during analysis is a determining factor. As stated by Braun and Clarke [34], the sample size cannot be determined in advance.

With regard to the quantitative phase, the literature indicates that this type of descriptive quantitative methodology is carried out on a fairly large patient population, generally 300 to 400 patients [39, 42]. It is challenging to determine the requisite number of subjects in this study, as there is no primary hypothesis to be tested. However, it is crucial to have sufficient data to identify potential influencing factors with sufficient statistical power. Therefore, we anticipate 300 subjects in this part of the study.

#### Feasibility

One of the challenges will be to recruit at least 300 patients. It is well documented in the literature that there is a significant phenomenon of gatekeeping [43] (« the process by which those involved in the research process prevent the participation of eligible patients in clinical research ») in palliative care. This can limit inclusions. As one of the team’s researchers has worked on this subject, we hope that our experiment will help to mitigate this risk. Furthermore, the inclusion of patients in the design of the project could have reduced this risk. Currently, there are no patients with advanced cancer in our working groups, which is due to the fragility of their clinical condition. This is another weakness of the study.

In addition, the multidisciplinary team is in a position to conduct this study, given the active file of patients in the various centers and the fact that these centers cover two departments. Given the high prevalence of cancer and PIMs, it is reasonable to expect the target number of participants within 12 months.

### Dissemination

The findings of the research will be disseminated through the publication of articles in international peer-reviewed journals and the presentation of research at national and international conferences. Additionally, communication initiatives aimed at the general public and patients will be conduct at public conferences. Training seminars on the subject will also be organized for healthcare professionals.

## Conclusion

The disparate outcomes will enhance our comprehension of the perception of deprescribing in palliative oncology care, thereby enabling the development of tailored approaches that align with the distinctive characteristics of our population. This project will facilitate the integration of care, research, and teaching. These findings may be employed to create deprescribing tools for the various healthcare professionals involved in (de)prescribing.

## Supporting information

S1 Checklist SPIRIT check-list. (DOC)

S1 File STOPPfrail 2. (DOCX)

S2 File Interview guide. (DOC)

S3 File rPATD. (DOCX)

S4 File BMQ. (DOCX)

S5 File FCCHL /HLS14. (DOCX)

S6 File Written information and consent for patient. (PDF)

S7 File Authorization form voice recording and interview transcription. (PDF)

S8 File Protocol French version. (DOCX)

S9 File Protocol English version. (DOCX)
