# Supplementary material for: DEprescribing: Perceptions of PAtients living with advanced cancer. A multicentre, prospective mixed observational study protocol
Source: PLoS One. 2024 Aug 20;19(8):e0305737. doi: 10.1371/journal.pone.0305737 (PMC11335145; doi:10.1371/journal.pone.0305737)

**STOPPFRAIL version 2 (French translation)**

| STOPPFrail est une liste d'indicateurs de prescription potentiellement inappropriés conçus pour aider les médecins à prendre des décisions de déprescription. Elle s'adresse initialement aux personnes âgées ayant une espérance de vie limitée et pour lesquelles l'objectif des soins est d'optimiser la qualité de vie et de minimiser le risque de morbidité liée aux médicaments.  Les candidats appropriés à la déprescription guidée par STOPPFrail répondent généralement à TOUS les critères suivants : 1. Activités de dépendance de la vie quotidienne (c'est-à-dire aide à l'habillage, à la toilette, au transfert, à la marche) et/ou maladie chronique grave et/ou maladie terminale.  2. Fragilité sévère et irréversible, c'est-à-dire risque élevé de complications médicales aiguës et de détérioration clinique.  3. Le médecin supervisant les soins du patient ne serait pas surpris si le patient décédait au cours des 12 prochains mois. | |
| --- | --- |
| **Section A : Général** | • Tout médicament que le patient ne parvient pas à prendre ou à tolérer de manière persistante malgré une éducation adéquate et la prise en compte de toutes les formulations appropriées.  • Tout médicament sans indication clinique claire. (ex : IPP…)  • Tout médicament pour les symptômes désormais résolus (par exemple douleur, nausée, vertiges, prurit) |
| **Section B : Système de cardiologie** | • Thérapies hypolipidémiantes (statines, ézétimibe, chélateurs des acides biliaires, fibrates, acide nicotinique, lomitapide et acipimox).  • Traitements antihypertenseurs : Réduisez ou arrêtez soigneusement ces médicaments chez les patients présentant une tension artérielle systolique (PAS) persistante <130 mmHg. Un objectif de PAS approprié chez les personnes âgées fragiles est de 130 à 160 mmHg. Avant d'arrêter, déterminez si le médicament traite d'autres affections (par exemple, un bêtabloquant pour le contrôle de la fréquence de la fibrillation auriculaire, des diurétiques pour l'insuffisance cardiaque symptomatique).  • Thérapie anti-angineuse (en particulier les nitrates, le nicorandil, la ranolazine) : Aucun de ces médicaments anti-angineux n'a démontré sa capacité à réduire la mortalité cardiovasculaire ou le taux d'infarctus du myocarde. Essayez de réduire et d'arrêter soigneusement ces médicaments chez les patients qui n'ont présenté aucun symptôme angineux signalé au cours des 12 mois précédents ET qui ne présentent aucune preuve prouvée ou objective de maladie coronarienne. |
| **Section C : Système de coagulation** | • Antiplaquettaires : Aucune preuve de bénéfice pour la prévention cardiovasculaire primaire (par opposition à la prévention secondaire).  • Aspirine pour la prévention des accidents vasculaires cérébraux en cas de fibrillation auriculaire : L'aspirine n'a que peu ou pas de rôle dans la prévention des accidents vasculaires cérébraux chez les personnes âgées fragiles qui ne sont pas candidates à un traitement anticoagulant et peut augmenter considérablement le risque de saignement. |
| **Section D : Système nerveux central** | • Antipsychotiques neuroleptiques chez les patients atteints de démence : viser à réduire la dose et à arrêter ces médicaments chez les patients qui les prennent pendant plus de 12 semaines s'il n'y a pas de caractéristiques cliniques actuelles de symptômes comportementaux et psychiatriques de démence (SCPD).  • Mémantine : Arrêter et surveiller les patients atteints de démence modérée à sévère, à moins que la mémantine n'ait clairement amélioré les SCPD. |
| **Section E : Système gastro-intestinal** | • Inhibiteurs de la pompe à protons : Réduire la dose des inhibiteurs de la pompe à protons lorsqu'ils sont utilisés à la dose thérapeutique complète ≥ 8 semaines, sauf symptômes dyspeptiques persistants à une dose d'entretien plus faible.  • Antagoniste des récepteurs H2 : Réduire la dose des antagonistes des récepteurs H2 lorsqu'ils sont utilisés à dose thérapeutique complète pendant ≥ 8 semaines, sauf symptômes dyspeptiques persistants à une dose d'entretien plus faible. |
| **Section F : Système respiratoire** | • Théophylline et aminophylline : Ces médicaments ont un index thérapeutique étroit, ont un bénéfice thérapeutique douteux et nécessitent une surveillance des taux sériques et interagissent avec d'autres médicaments couramment prescrits, exposant les patients à un risque accru d'EIM.  • Antagonistes des leucotriènes (montélukast, zafirlukast) : ces médicaments n'ont pas de rôle prouvé dans la maladie pulmonaire obstructive chronique ; ils ne sont indiqués que dans l'asthme. |
| **Section G : Système musculo-squelettique** | • Suppléments de calcium : il est peu probable qu'ils présentent un quelconque bénéfice à court terme, sauf hypocalcémie symptomatique prouvée.  • Vitamine D (ergocalciférol et colécalciférol) : manque de preuves claires pour soutenir l'utilisation de la vitamine D pour prévenir les chutes et les fractures, les événements cardiovasculaires ou les cancers.  • Médicaments anti-résorption/anabolisants osseux *pour l'ostéoporose* (bisphosphonates, strontium, tériparatide, dénosumab)  • Anti-inflammatoires non stéroïdiens oraux à long terme : risque accru d'effets secondaires (par exemple, ulcère gastroduodénal, saignement, aggravation de l'insuffisance cardiaque) lorsqu'ils sont pris régulièrement pendant ≥2 mois.  • Corticostéroïdes oraux à long terme : risque accru d'effets secondaires majeurs (par exemple fractures de fragilité, myopathie proximale, ulcère gastroduodénal) lorsqu'ils sont pris régulièrement pendant ≥ 2 mois. Envisagez une réduction prudente de la dose et un arrêt du traitement. |
| **Section H : Système urogénital** | • Médicaments contre l'hyperplasie bénigne de la prostate (inhibiteurs de la 5-alpha réductase et alpha-bloquants) chez les patients masculins cathétérisés : aucun bénéfice avec un cathétérisme vésical à long terme.  • Médicaments contre l'hyperactivité vésicale (antagonistes muscariniques et mirabegron) : aucun bénéfice chez les patients présentant une incontinence urinaire persistante et irréversible, sauf antécédents clairs d'hyperactivité détrusorienne douloureuse. |
| **Section I : Système endocrinien** | • Médicaments antidiabétiques : désintensifier le traitement. Évitez les objectifs d'HbA1c (HbA1C <7,5 % [58 mmol/ml] associé à un préjudice net dans cette population). L'objectif des soins est de minimiser les symptômes liés à l'hyperglycémie (ex : soif excessive, polyurie). |
| **Section J : Divers** | • Suppléments combinés multivitaminés : Arrêtez-les lorsqu'ils sont prescrits pour la prophylaxie plutôt que pour le traitement de l'hypovitaminose.  • Acide folique : Arrêtez lorsque le traitement est terminé. La durée habituelle du traitement est de 1 à 4 mois, sauf malabsorption, malnutrition ou utilisation concomitante de méthotrexate.  • Suppléments nutritionnels : Arrêtez-les lorsqu'ils sont prescrits à titre prophylactique plutôt que pour le traitement de la malnutrition. |

**STOPPFRAIL version 2 (original English version)**


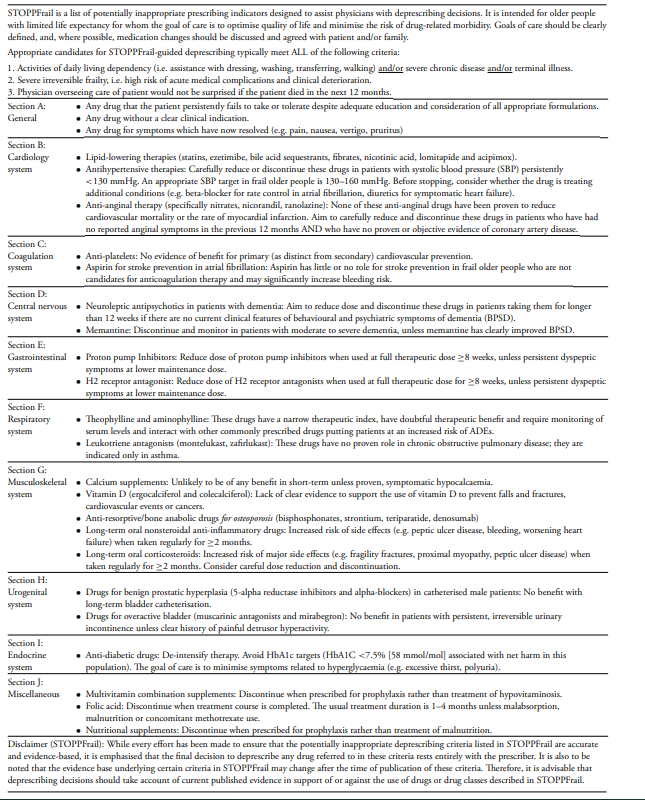

Supplement: S1 File — (DOCX) [file pone.0305737.s002.docx]
