# Supplementary material for: DEprescribing: Perceptions of PAtients living with advanced cancer. A multicentre, prospective mixed observational study protocol
Source: PLoS One. 2024 Aug 20;19(8):e0305737. doi: 10.1371/journal.pone.0305737 (PMC11335145; doi:10.1371/journal.pone.0305737)
Supplement: S2 File — (DOC) [file pone.0305737.s003.doc]

**French Version of interview guide**

**Guide d’entretien :**

**Introduction :**

*« Bonjour,*

*Merci d’être présent ici aujourd’hui.*

*Je me présente :****nom, prénom, chercheur***

*Dans le cadre de l’étude DéPal, nous nous intéressons à la perception des patients atteints de cancer d’un potentiel arrêt de certains traitements, des médicaments qui ne seraient plus appropriés.*

*L’objectif de notre étude est de mieux accompagner la prise en charge des patients, notre discussion d'aujourd'hui a pour objectif d'en savoir plus sur la façon dont vous percevez vos médicaments et de mieux comprendre votre opinion sur l'arrêt de certains médicaments notamment ceux qui ne seraient plus appropriés.*

*Cet entretien va durer environ 30 à 45 min. à tout moment vous pouvez décider d’arrêter.*

*Pour éviter de déformer vos propos et rester attentif à ce que vous me dites, êtes-vous d’accord pour que j’enregistre nos échanges ?*

*Je pourrais aussi prendre de temps en temps quelques notes.*

*Je tiens à préciser que je n’attends aucune réponse en particulier.*

*Si vous êtes toujours d’accord, je vais commencer à enregistrer. »*

**Brise-Glace**

**Pouvez-vous me dire comment vous vous sentez aujourd’hui ?**

**Partie 1**

**- Perception des médicaments (évaluation du fardeau) et de la polymédication**

**-Évolution de cette perception au cours de la maladie**

**- Adéquation du traitement actuel**

Question de lancement : **Quelle est votre expérience en matière de prise de médicaments ?**

Relance : Au cours des derniers mois ? quel est votre perception des traitements que vous prenez ?

En termes de quantité ?

En termes de rythme des prises ?

En termes d’utilité ? besoins ?

Les -

En termes de contraintes liés aux traitements ?

En terme d’effet indésirables ?

Les + :

En termes d’avantages liés aux traitements ?

Relance : Ces perceptions ont-elles évolué ces derniers temps ? et comment ?

Relance : s’il y avait des choses à faire évoluer dans vos traitements, qu’est-ce que se serait ? Qu’est-ce qui vous amène à penser ça ?

**Partie 2**

**Implication du patient dans les décisions concernant ces traitements**

Question de lancement :

**Quelle est votre implication dans le choix des traitements que vous prenez ?**

Relance : Connaissez-vous les traitements que vous prenez actuellement ?

Relance : Savez-vous pourquoi on vous a prescrit ces traitements ?

Relance : Souhaitez-vous êtes informé sur vos médicaments ?

Relance : Comment souhaiteriez-vous être informé sur vos médicaments ? par qui ? quand ? et par quel moyen ?

Relance : Dans votre cas, racontez-moi comment ça s’est passé la mise en place de vos traitements ? Décrivez-moi votre rôle dans la prise de ces décisions ? et quel rôle souhaiteriez-vous ?

**Partie 3**

**Perception d’une potentielle déprescription proposée par le médecin**

Question de lancement :

**Les médecins passent en revue régulièrement la liste de médicaments des patients. Si votre médecin vous disait qu'il était possible d'arrêter un ou plusieurs de vos médicaments, que penseriez-vous ?**

Relance : Pourquoi pensez-vous cela ?

Relance : Qu’est-ce que cela signifierait ?

Relance : Quelles seraient vos émotions ? soulagé ? inquiet ?

Relance : Comment souhaiteriez-vous que cela soit abordé ?

Relance : Que pensez-vous d’avoir l’avis d’une autre personne et si oui qui ? + quelles suggestions : seriez-vous pour …médecin traitant ou oncologue…

**Conclusion**

*Nous allons maintenant conclure.*

Question :

**Selon vous, quel est le point important auquel nous devrions prêter attention lorsqu'il s'agit de réfléchir à la question de l’arrêt de médicaments notamment** **des médicaments qui ne seraient plus appropriés ?**

Question de clôture :

**Que souhaitez-vous ajouter, préciser ?**

*« Cet entretien arrive à sa fin.*

*Je tiens à vous remercier pour avoir répondu à ces questions. »*

**English version of interview guide**

**Interview Guide :**

**Introduction :**

"*Hello,*

*Thank you for being here today.*

*Let me introduce myself: last name, first name, researcher*

*As part of the DéPal study, we are interested in cancer patients' perceptions of the potential discontinuation of certain treatments, drugs that would no longer be appropriate.*

*The aim of our study is to provide better support for patient care, and the purpose of today's discussion is to find out more about how you perceive your medication, and to better understand your opinion on stopping certain drugs, particularly those that are no longer appropriate.*

*This interview will last around 30 to 45 minutes. At any time, you can decide to stop.*

*To avoid misrepresenting what you're saying, and to remain attentive to what you're telling me, do you agree to me recording our exchanges?*

*I could also take a few notes from time to time.*

*I'd like to make it clear that I'm not expecting any particular answer.*

*If you still agree, I'll start recording*."

**Icebreaker**

**Can you tell me how you're feeling today?**

**Part 1**

**- Perception of medication (burden assessment) and polymedication**

**-Changes in perception over the course of the disease**

**- Adequacy of current treatment**

Opening question: **What is your experience of taking medication?**

Relaunch: Over the past few months, how do you perceive the treatments you are taking?

In terms of quantity?

In terms of frequency?

In terms of usefulness? needs?

The -

In terms of treatment constraints?

In terms of side effects?

The + :

In terms of treatment benefits?

Relaunch: Have these perceptions changed recently? How have they changed?

Relaunch: If there were things to change in your treatments, what would they be? What makes you think so?

**Part 2**

**Involving patients in treatment decisions**

Opening question: **How involved are you in choosing the treatments you take?**

Relaunch: Do you know the treatments you are currently taking?

Relaunch: Do you know why you have been prescribed these treatments?

Relaunch: Would you like to be informed about your medication?

Relaunch: How would you like to be informed about your medication? by whom? when? and by what means?

Relaunch: In your case, can you tell me what it was like to start taking your medication? Describe your role in making these decisions? and what role would you like to play?

**Part 3**

**Perception of a potential deprescription proposed by the physician**

Opening question:

**Physicians regularly review patients' medication lists. If your physician told you it was possible to stop one or more of your medications, what would you think?**

Relaunch: Why would you think that?

Relaunch: What would that mean?

Relaunch: How would you feel? Relieved? Worried?

Relaunch: How would you like this to be addressed?

Relaunch: How would you feel about having another person's opinion, and if so, who? + any suggestions: would you be in favor of ...attending physician or oncologist...

**Conclusion**

*We'll now come to a close.*

Question:

**In your opinion, what is the important point we should pay attention to when it comes to thinking about the issue of discontinuing medications, particularly medications that would no longer be appropriate?**

Closing question:

**What would you like to add?**

*"This interview is coming to an end.*

*I'd like to thank you for answering these questions."*
