# Supplementary material for: DEprescribing: Perceptions of PAtients living with advanced cancer. A multicentre, prospective mixed observational study protocol
Source: PLoS One. 2024 Aug 20;19(8):e0305737. doi: 10.1371/journal.pone.0305737 (PMC11335145; doi:10.1371/journal.pone.0305737)

**rPATD (French version)**

•S'il vous plaît, indiquer dans quelle mesure vous êtes en accord ou en désaccord avec ces propositions en mettant une croix dans la case appropriée (réponse qui vous correspond le plus).

•Il n'y a pas de bonnes ou de mauvaises réponses.

| Tout à fait  d’accord | D’accord | Incertain | En  désaccord | Fortement en désaccord |
| --- | --- | --- | --- | --- |

| Q1 | Je dépense beaucoup d’argent pour mes médicaments |
| --- | --- |
| Q2 | Prendre mes médicaments tous les jours n’est pas très pratique |
| Q3 | Je trouve que je prends un grand nombre de médicaments |
| Q4 | Je trouve que mes médicaments sont une contrainte pour moi |
| Q5 | Parfois, je pense que je prends trop de médicaments |
| Q6 | Je trouve que je prends peut-être un ou plusieurs médicaments dont je n’ai plus besoin |
| Q7 | J’aimerais essayer d’arrêter un de mes médicaments pour voir comment je me sentirais sans celui-ci |
| Q8 | J’aimerais que mon médecin réduise la dose d’un ou plusieurs de mes médicaments |
| Q9 | Je pense qu’un ou plusieurs de mes médicaments ne sont peut-être pas efficaces |
| Q10 | Je crois qu’un ou plusieurs de mes médicaments peuvent me donner en ce moment des effets indésirables |
| Q11 | Je serais réticent(e) à arrêter un médicament que je prends depuis longtemps |
| Q12 | Si un de mes médicaments était arrêté, je serais inquiet(e) de passer à côté de ses futurs bénéfices |
| Q13 | Je suis inquiet(e)/stressé(e) chaque fois que mes médicaments sont changés |
| Q14 | Si mon médecin me recommandait d’arrêter un médicament, j’aurais le sentiment qu’il renonce à me soigner. |
| Q15 | J’ai déjà eu une mauvaise expérience quand un médicament a été arrêté |
| Q16 | Je comprends bien pourquoi on m’a prescrit chacun de mes médicaments |
| Q17 | Je sais exactement quels médicaments je prends en ce moment et/ou je tiens à jour une liste de médicaments |
| Q18 | J’aime en savoir le plus possible sur mes médicaments |
| Q19 | J’aime être impliqué(e) avec mes médecins dans les décisions qui sont prises concernant mes médicaments |
| Q20 | Je demande toujours au médecin, au pharmacien ou à un autre professionnel de la santé s’il y a quelque chose que je ne comprends pas au sujet de mes médicaments |
| Q21 | Si mon médecin disait que cela est possible, je serais prêt(e) à arrêter un ou plusieurs de mes médicaments habituels |
| Q22 | Globalement, je suis satisfait(e) de mes médicaments actuels |

**rPATD (English version)**


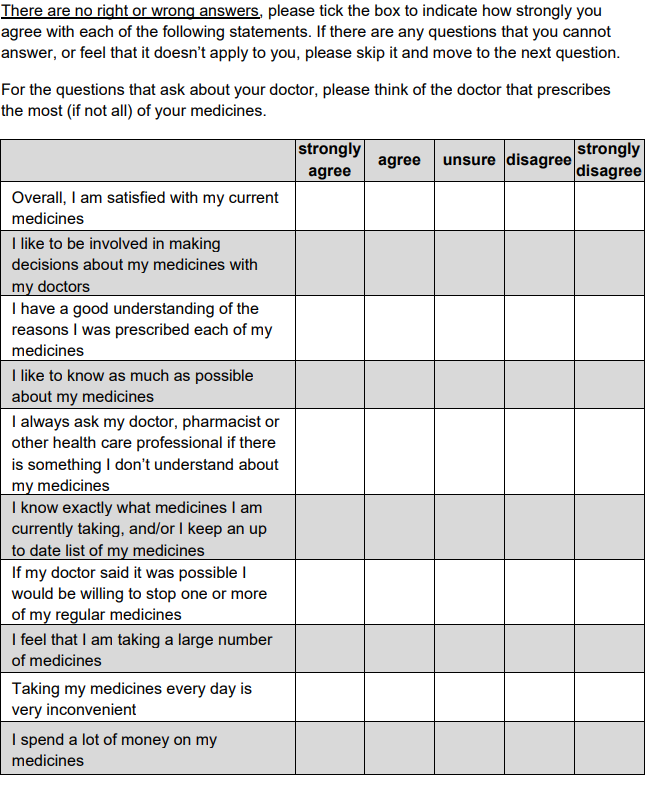


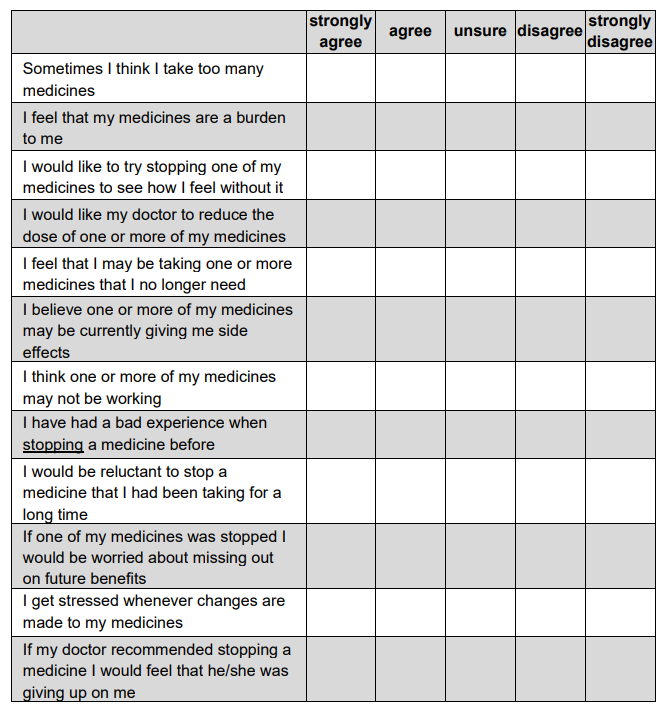

Supplement: S3 File — (DOCX) [file pone.0305737.s004.docx]
