# Supplementary material for: DEprescribing: Perceptions of PAtients living with advanced cancer. A multicentre, prospective mixed observational study protocol
Source: PLoS One. 2024 Aug 20;19(8):e0305737. doi: 10.1371/journal.pone.0305737 (PMC11335145; doi:10.1371/journal.pone.0305737)
Supplement: S4 File — (DOCX) [file pone.0305737.s005.docx]

**French version of the Beliefs about Medicines Questionnaire.**

Nous voudrions vous poser des questions sur votre point de vue personnel concernant les traitements qui vous sont prescrits. Les déclarations suivantes ont été faites par d'autres personnes concernant leurs médicaments.

**•S'il vous plaît, indiquer dans quelle mesure vous êtes en accord ou en désaccord avec eux en faisant une croix dans la case appropriée (réponse qui vous correspond le plus).**

**•Il n'y a pas de bonnes ou de mauvaises réponses.**

**Nous sommes intéressés par vos opinions personnelles**

| Tout à fait  d’accord | D’accord | Incertain | En  désaccord | Fortement en désaccord |
| --- | --- | --- | --- | --- |

**Croyances spécifiques :**

1. Ma santé, aujourd’hui, dépend de mon traitement.

2. Avoir à prendre un traitement m’inquiète.

3. Ma vie serait impossible sans mon traitement.

4. Sans mon traitement, je serais très malade.

5. Je m’inquiète parfois à propos des effets à long terme de mon traitement.6. Mon traitement est un mystère pour moi.

7. Ma santé future dépend de mon traitement.

8. Mon traitement perturbe ma vie.

9. Je suis parfois inquiet(e) de devenir trop dépendant(e) de mon traitement.

10. Mon traitement empêche mon état d’empirer.

**Croyances générales** :

11. Les médecins utilisent trop de traitements.

12. Les personnes qui prennent des médicaments devraient arrêter leur traitement de temps en temps.

13. La plupart des traitements provoquent une dépendance.

14. Les remèdes naturels sont plus sûrs que les traitements médicaux.

15. Les traitements font plus de mal que de bien.

16. Tous les traitements sont des poisons.

17. Les médecins accordent trop de confiance aux traitements.

18. Si les médecins passaient plus de temps avec les patients, ils prescriraient moins de traitements.

**English version of the Beliefs about Medicines Questionnaire.**

We'd like to ask you some questions about your personal views on your prescribed treatments. The following statements have been made by other people about their medication.

-Please indicate the extent to which you agree or disagree with them by placing a cross in the appropriate box (the answer that best corresponds to you).

-There are no right or wrong answers.

We are interested in your personal opinions

| Strongly  Agree | Agree | Uncertain | Disagree | Strongly disagree |
| --- | --- | --- | --- | --- |

1. My health, at present, depends on my medicines.
2. My life would be impossible without my medicines.
3. Without my medicines I would be very ill.
4. My health in the future will depend on my medicines.
5. My medicines protect me from becoming worse.
6. Specific-Concern (BMQ-SC)
7. Having to take medicines worries me.
8. I sometimes worry about long-term effects of my medicines.
9. My medicines are a mystery to me.
10. My medicines disrupt my life.
11. I sometimes worry about becoming too dependent on my medicines.
12. Doctors use too many medicines.
13. Natural remedies are safer than medicines.
14. Doctors place too much trust on medicines.
15. If doctors had more time with patients they would prescribe fewer medicines.
16. People who take medicines should stop their treatment for a while every now and
17. again.
18. Most medicines are addictive.
19. Medicines do more harm than good.
20. All medicines are poisons
