# Supplementary figures and images for: DEprescribing: Perceptions of PAtients living with advanced cancer. A multicentre, prospective mixed observational study protocol
Source: PLoS One. 2024 Aug 20;19(8):e0305737. doi: 10.1371/journal.pone.0305737 (PMC11335145; doi:10.1371/journal.pone.0305737)

**French version of FCCHL / HLS14**


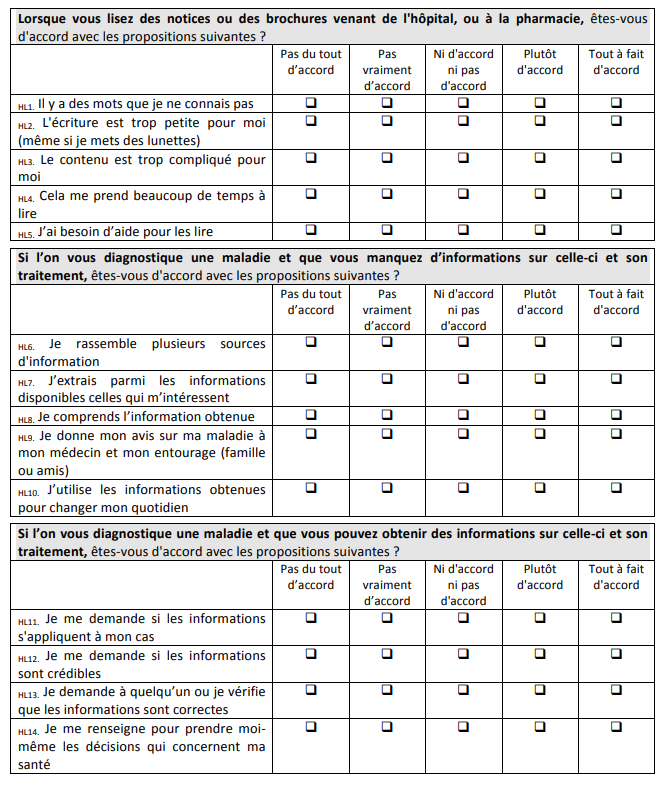


**Original Japanese/English version of FCCHL / HLS14**


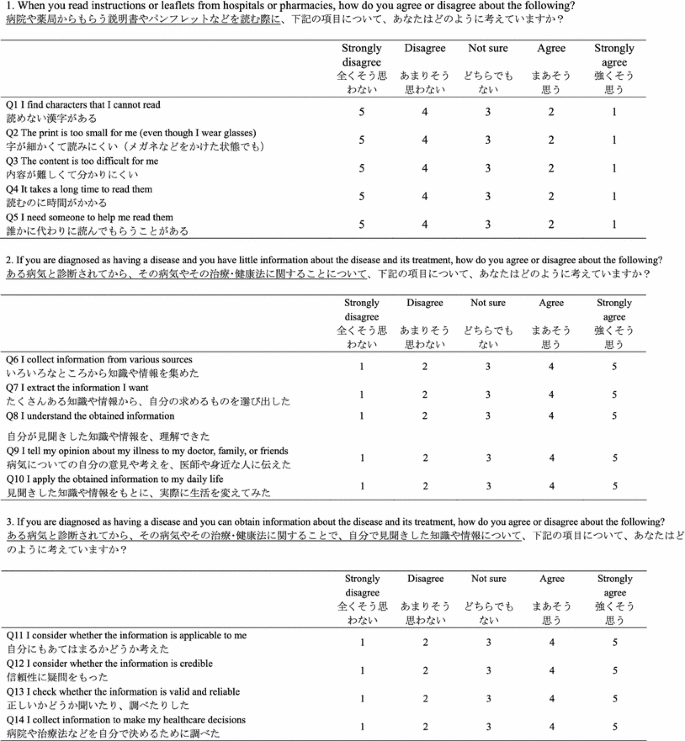

Supplement: S5 File — (DOCX) [file pone.0305737.s006.docx]
