# Supplementary material for: DEprescribing: Perceptions of PAtients living with advanced cancer. A multicentre, prospective mixed observational study protocol
Source: PLoS One. 2024 Aug 20;19(8):e0305737. doi: 10.1371/journal.pone.0305737 (PMC11335145; doi:10.1371/journal.pone.0305737)
Supplement: S6 File — (PDF) [file pone.0305737.s007.pdf]

Note d'information pour la participation à la recherche

« DEprescribing: perceptions of PAtients Living with advanced cancer
A multicentre, prospective mixed observational study »
« La déprescription: perceptions chez les patients vivant avec un cancer avancé. Une étude multicentrique observationnelle prospective mixte »

DEPAL

Étude qualitative

Promoteur : CHU de Nantes
RC23_0563

Ce document est remis à la personne participant à la recherche

Madame, Monsieur,

Le Centre Hospitalier et Universitaire de Nantes souhaite mener une recherche dont il est le promoteur (c'est à dire qu'il en est responsable et qu'il l'organise). L'objectif de cette recherche est de décrire, comprendre et expliquer les attitudes et croyances des patients vivant avec un cancer avancé (c'est-à-dire avec un cancer localement avancé ou métastatique) concernant la déprescription (*ceci correspond à la réduction de la dose et/ou l'arrêt des médicaments qui peuvent nuire, qui peuvent ne plus fournir de bénéfice, ou être considérés comme n'étant plus appropriés*).

L'étude comporte deux périodes différentes :
- Une première période qui correspond à un entretien semi-dirigé **auquel il vous est proposé de participer**;
- une seconde qui comporte un recueil de données avec passation de questionnaires qui débutera dans 8 mois et pour lequel il ne vous sera pas demandé de participer.

Nous vous proposons de réaliser ce jour ou dans les 15 jours à venir un **entretien semi-dirigé en tête à tête qui** porte sur votre perception des médicaments et notamment ceux qui potentiellement ne seraient plus appropriés. Un seul entretien sera réalisé dont la durée moyenne est estimée à 45 min environ (donné à titre indicatif). Un double enregistrement (2 dictaphones) numérisé de l'entretien sera réalisé. **Les entretiens individuels seront réalisés par deux chercheurs**. **Les entretiens seront retranscrits** intégralement en respectant de manière rigoureuse vos propos par un prestataire français externe au CHU de Nantes. Les enregistrements audio de l'entretien seront détruits après la retranscription écrite.

Si pour des raisons sanitaires ou des contraintes d'agendas, une rencontre physique ne peut avoir lieu, l'entretien se fera par visioconférence ou par téléphone.

Cette recherche est réalisée en collaboration avec l'équipe INSERM UMR 1246 SPHERE.

Votre participation à la recherche, au cas où vous donneriez votre accord, ne pourra vous être confirmée qu'à la condition que vous remplissiez tous les critères d'inclusion pour participer à cette recherche.

Cette recherche ne présente pas de risque pour votre santé. Les résultats qui en seront issus serviront à la recherche et n'apporteront pas d'informations supplémentaires concernant votre santé. Ils favoriseront le développement des connaissances dans le domaine de la prise en charge médicamenteuse chez des patients vivants avec un cancer avancé et devront être confirmés, ensuite, par des études cliniques complémentaires, afin de permettre l'essor de nouvelles méthodes de gestion des thérapeutiques.

TRAITEMENT DES DONNEES

Le traitement de vos données personnelles a pour fondement juridique l'exécution d'une mission d'intérêt public que constitue cette recherche (article 6.1.e du Règlement (UE) 2016/679 du Parlement européen et du Conseil du 27 avril 2016 relatif à la protection des personnes physiques à l'égard du traitement des données à caractère personnel et à la libre circulation de ces données (RGPD)). Il s'agit d'une des exceptions prévues par l'article 9 du même texte, permettant le traitement des données de santé.

Le traitement est conforme à la méthodologie de référence MR004 publiée par la CNIL.

Dans le cadre de cette recherche, un traitement informatique de vos données personnelles va être mis en œuvre : cela permettra d'analyser les résultats de la recherche et de remplir l'objectif de la recherche.

Pour cela, les données vous concernant seront recueillies dans un fichier informatique auquel aura accès le Promoteur de la recherche (CHU de Nantes). Des données de suivi pourront être recueillies au-delà de la stricte durée de l'étude ;

Afin d'assurer leur confidentialité, vos données seront identifiées par un numéro de code et vos initiales.

Les personnes intervenant dans la recherche dans le cadre de leurs missions auront accès à vos données codées.

Les seules personnes qui auront accès à vos données directement identifiantes sont les personnes qui assurent votre prise en charge dans le cadre de la recherche et les personnes responsables du contrôle et de l'assurance qualité de la recherche.

Vos données pourront, dans des conditions assurant leur confidentialité, via des plateformes ou serveurs sécurisés, faire l'objet de transferts nationaux ou internationaux (comme, par exemple, aux personnes ou sociétés agissant pour le compte du CHU, aux autorités sanitaires habilitées) pour cette étude, ou pour d'autres recherches ultérieures, exclusivement à des fins scientifiques. Au sein de l'Europe la protection de vos données est garantie (Règlement européen UE 2016/679). Hors Europe vos données pourront être transmises dans des états n'ayant pas le même niveau d'exigence en termes de protection des données. Le cas échéant, le Promoteur prendra toutes les mesures nécessaires pour protéger les données recueillies et devra s'engager à assurer un niveau de sécurité équivalent à celui couvert par les lois françaises et européennes pour les données envoyées à l'étranger. Si vous le souhaitez, vous pouvez obtenir la copie de l'ensemble des mesures prises pour assurer l'ensemble de la protection de vos données auprès du Délégué à la Protection des Données (DPD) voir en fin de document, paragraphe « vos contacts ».

Vos données pseudonymisées (identifiées par un numéro de code et vos initiales) seront susceptibles d'être exploitées dans le cadre de publications ou de communications ; dans ce cas votre anonymat sera préservé.

Conformément aux dispositions de la loi relative à l'informatique aux fichiers et aux libertés (loi modifiée du 6 janvier 1978), et du Règlement (UE) 2016/679 du Parlement européen et du Conseil du 27 avril 2016 relatif à la protection des personnes physiques à l'égard du traitement des données à caractère personnel et à la libre circulation de ces données (RGPD), vous disposez d'un droit d'accès, de rectification, de limitation et d'opposition du traitement de vos données personnelles. Si vous décidez de retirer votre consentement pour participer à la recherche, les données obtenues avant que celui-ci n'ait été retiré seront utilisées conformément à l'article L.1122-1-1 du CSP. Les données recueillies après le retrait de votre consentement ne seront pas utilisées pour cette recherche et resteront destinées à l'usage strict du soin. Vous pouvez également porter une réclamation auprès d'une autorité de contrôle (CNIL pour la France : https://www.cnil.fr/fr/webform/adresser-une-plainte/).

Ces données pourront être utilisées lors de recherches ultérieures exclusivement à des fins scientifiques. Vous pouvez retirer votre consentement à cette utilisation ultérieure ou exercer votre faculté d'opposition à tout moment.

Vous pouvez également accéder directement ou par l'intermédiaire d'un médecin de votre choix à l'ensemble de vos données médicales en application des dispositions de l'article L 1111-7 du Code de la Santé Publique.

Vos données seront conservées tout au long de la recherche. Après la fin de la recherche, les données seront archivées pour une durée de 15 ans, puis détruites.

L'investigateur pourra vous informer, sur votre demande, des résultats globaux de cette recherche.

Pour en savoir plus ou exercer vos droits concernant vos données, voir en fin de document, paragraphe « vos contacts ».

Cette recherche est conforme :
- à la loi « Informatique et Libertés » du 6 janvier 1978 modifiée et la loi n° 2018-493 du 20 juin 2018 relative à la protection des données personnelles
- au Règlement (UE) 2016/679 du Parlement européen et du Conseil du 27 avril 2016 relatif à la protection des personnes physiques à l'égard du traitement des données à caractère personnel et à la libre circulation de ces données (RGPD)

Ce projet ainsi que le présent document ont été présentés au Groupe Nantais d'éthique dans le domaine de la Santé (GNEDS).

Votre participation à cette recherche est libre. Vous pouvez refuser de participer à cette recherche, et vous pouvez à tout moment vous retirer de cette recherche, sans préjudice. Cela n'aura aucune conséquence sur la qualité des soins qui vous seront donnés ; vous devez simplement en informer l'investigateur.

VOS CONTACTS :

Pour toute question concernant l'étude, retrait de consentement, ou pour exercer vos droits concernant vos données (accès, rectification, etc…), votre contact privilégié est :

L'investigateur coordonnateur de la recherche :

Dr EVIN Adrien

✉ Service Interdisciplinaire Douleur, Soins Palliatifs et de Support, Médecine intégrative CHU de NANTES - Hôpital Guillaume et René LAENNEC Boulevard Jacques MONOD 44093 NANTES cedex 1

Pour toute question générale sur le traitement de vos données :

Le promoteur de la recherche, responsable du traitement :

CHU de Nantes, direction de la recherche et de l'innovation

5 allée de l'Ile Gloriette, 44093 NANTES Cedex 1

Le Délégué à la Protection des Données (DPD) / Data Protection Officer (DPO) :

A compléter par la personne participant à la recherche

Prénom/Nom : …………………………………………………………..

J'accepte que mes données soient utilisées pour cette recherche : ☐ **oui** ☐ **non**

Si OUI : **J'accepte d'être contacté(e) par téléphone ou par mail par le CHU de Nantes pour réaliser un entretien semi-dirigé avec un médecin du CHU de Nantes** : ☐ **oui** ☐ **non**

Date : ………./………/…………
Signature :

Merci de conserver cette notice d'information et de consentement
