# Supplementary material for: DEprescribing: Perceptions of PAtients living with advanced cancer. A multicentre, prospective mixed observational study protocol
Source: PLoS One. 2024 Aug 20;19(8):e0305737. doi: 10.1371/journal.pone.0305737 (PMC11335145; doi:10.1371/journal.pone.0305737)
Supplement: S7 File — (PDF) [file pone.0305737.s008.pdf]

CENTRE HOSPITALIER UNIVERSITAIRE DE NANTES

Formulaire d'autorisation de droit à la voix, de leur reproduction et exploitation
Protocole de recherche « DEPAL » - RC23_0563

La présente autorisation définit dans les strictes conditions suivantes l'utilisation et la diffusion des prises de vue qui s'inscrivent dans le cadre suivant présenté ci-dessous.

Objet de la prise d'image ou de tout autre attribut de la personnalité : *Enregistrement vocale d'un entretient Médecin/patient pour retranscription écrite post-entretient dans le cadre de l'étude DEPAL (RC23_0563) dont la note de présentation de l'étude a été remise à l'intéressé(e) au moment de son recrutement dans le projet.*
Personne en charge de l'image ou de tout autre attribut de la personnalité : Docteur Adrien EVIN

Je soussigné(e) (*Nom, Prénom*) ______________________________

Joignable au (*adresse, coordonnées téléphoniques ou mail*) _____________________________________.

Autorise le CHU de Nantes à :

☐ **<u>Enregistrer ma voix lors d'un entretien en présentiel ou en distanciel dans le cadre de l'étude citée ci-dessous</u>.**

Cette autorisation est consentie dans les strictes conditions suivantes :

L'intéressé(e) autorise expressément le CHU de Nantes à faire usage des enregistrements de la voix, dans le cadre du projet de recherche suivant :

DEPAL : « La déprescription : perceptions chez les patients vivant avec un cancer avancé. Une étude multicentrique observationnelle prospective mixte »

Ref. : RC23_0563

La personne en charge de l'enregistrements s'engage à détruire les enregistrements de la voix dès lors que la retranscription écrite de ce dernier aura été effectué (avant la fin de la recherche).

La présente autorisation est délivrée uniquement au CHU de Nantes. J'autorise toutefois le CHU de Nantes à recourir à des tiers qu'elle aura dûment habilités, pour réaliser la retranscription de mon enregistrement.

La présente autorisation est conservée par l'établissement de santé assurant ma prise en charge et m'ayant transmis les informations en lien avec la recherche DEPAL.

La présente autorisation est soumise au droit français ; tout litige relatif à son interprétation ou à son exécution sera soumis aux tribunaux français.

Sous réserve du respect de l'ensemble de ces conditions, je délivre mon consentement libre et éclairé.

Fait à _____________________, le __/__/____

Signature manuscrite de l'intéressé(e)

Cas d'une recherche : Document original à conserver dans le classeur investigateur, remettre une copie à l'intéressé(e)

S'agissant de vos données personnelles hors données médicales, conformément au Règlement européen général relatif à la protection des personnes physiques à l'égard des traitements des données à caractère personnel et à la libre circulation de ces données (RGPD) et à la « Loi informatique et Libertés » du 6 janvier 1978 modifiée, vous bénéficiez d'un droit d'accès, de rectification, d'opposition, de portabilité, à l'effacement (ou « droit à l'oubli ») de celles-ci ou une limitation de traitement. Vous disposez également du droit de retirer votre consentement à tout moment en envoyant un mail à l'adresse : vosdonneespersonnelles@chu-nantes.fr précisant votre nom, prénom et date de naissance. Vous avez enfin la possibilité d'introduire une réclamation auprès d'une autorité de contrôle.
