# Supplementary material for: DEprescribing: Perceptions of PAtients living with advanced cancer. A multicentre, prospective mixed observational study protocol
Source: PLoS One. 2024 Aug 20;19(8):e0305737. doi: 10.1371/journal.pone.0305737 (PMC11335145; doi:10.1371/journal.pone.0305737)

***Protocole DEPAL study***

**«** **DEprescribing: perceptions of PAtients Living with advanced cancer.**  **A multicentre, prospective mixed observational study. »**

**Ref. : RC23_0563**

**Investigateur Coordonnateur :**

**Dr Adrien EVIN**

Praticien Hospitalier (MD, PhD student)

Unité de Soins Palliatifs (5 ème étage, aile Sud)

Service Interdisciplinaire Douleur, Soins Palliatifs et de Support, Médecine intégrative

CHU de NANTES - Hôpital Guillaume et René LAENNEC

Boulevard Jacques MONOD

44093 NANTES cedex 1

INSERM UMR 1246 SPHERE et UIC 22

Tel 02 53 48 27 36 [Adrien.evin@chu-nantes.fr](mailto:Adrien.evin@chu-nantes.fr)

**Méthodologiste :**

**Jean-Benoit Hardouin**

Maitre de conférences - Praticien Hospitalier, PhD

Service de Santé Publique - Plateforme de Méthodologie et Biostatistique - CHU de Nantes

INSERM UMR 1246-SPHERE

[jean-benoit.hardouin@univ-nantes.fr](mailto:jean-benoit.hardouin@univ-nantes.fr)

**Comité scientifique :**

**Marianne Bourdon**

Psychologue clinicienne, PhD

Institut de Cancérologie de l'Ouest, Nantes, Angers, France

INSERM UMR 1246 SPHERE

**Dr Jean-François HUON**

Maitre de conférences - Praticien Hospitalier, PharmD, PhD

Pharmacie Clinique & Santé publique, Faculté Pharmacie de Nantes, CHU de Nantes

INSERM UMR 1246 SPHERE

**Dr Pierre Nizet**

Assistant hospitalier universitaire, PharmD, PhD student

Pharmacie Clinique & Santé publique, Faculté Pharmacie de Nantes, CHU de Nantes

INSERM UMR 1246 SPHERE

**Pr Caroline Victorri-Vigneau**

PU-PH, PharmD, PhD, HDR

Service de Pharmacologie Clinique, Faculté de Médecine, CHU de Nantes

INSERM UMR 1246 SPHERE

**Promoteur :**
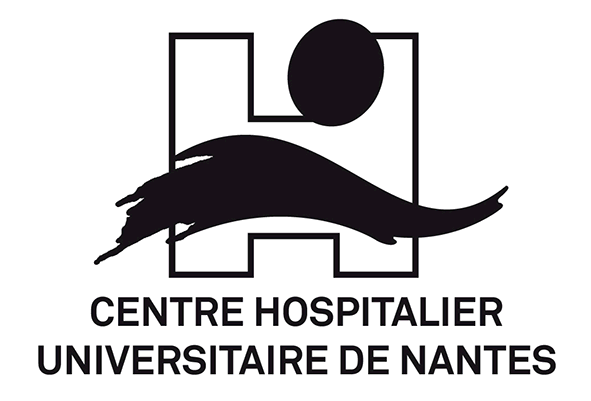
 **CHU de Nantes**


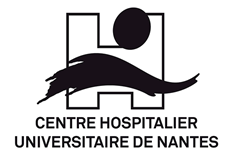
Direction des Affaires Médicales

et de la Recherche
5, allée de l’île Gloriette
44 093 Nantes cedex 01 (FRANCE)

[Bp-prom-regl@chu-nantes.fr](mailto:Bp-prom-regl@chu-nantes.fr)

Tel : 02 53 48 28 35
Fax : 02 53 48 28 36

***Table des Matières***

[1. INTRODUCTION/Contexte scientifique 3](#_Toc161245794)

[2. Justification de l’etude 3](#_Toc161245795)

[3. Objectifs et criteres de jugement 4](#_Toc161245796)

[3.1. Objectif et critère d’évaluation principal 4](#_Toc161245797)

[3.2. Objectifs et critères d’évaluation secondaires 5](#_Toc161245798)

[3.3. Étude Ancillaire : 5](#_Toc161245799)

[4. Population étudiée 5](#_Toc161245800)

[4.1. Description de la population 5](#_Toc161245801)

[4.2. Critères d’inclusion 6](#_Toc161245802)

[4.3. Critères de non-inclusion 6](#_Toc161245803)

[5. DESIGN ET Déroulement de l’étude 6](#_Toc161245804)

[5.1. Méthodologie générale de la recherche 6](#_Toc161245805)

[5.2. Calendrier de l’étude 10](#_Toc161245806)

[6. Aspects administratifs et réglementaires 12](#_Toc161245807)

[7. retombées attendues au plan scientifique et en termes de santé publique 12](#_Toc161245808)

[Liste des annexes 13](#_Toc161245809)

[ANNEXE 1: RÉFÉRENCES BIBLIOGRAPHIQUES 14](#_Toc161245810)

[ANNEXE 2 : LISTING DES INTERVENANT PRINCIPAUX 16](#_Toc161245811)

[ANNEXE 3 : QUESTIONNAIRES 17](#_Toc161245812)

[ANNEXE 4 : NOTES D’INFORMATION PATIENT 21](#_Toc161245813)

[ANNEXE 5 : AUTORISATION DE DROIT A LA VOIX 27](#_Toc161245814)

[ANNEXE 6 : Engagement de conformité à la MR004 28](#_Toc161245815)

# INTRODUCTION/Contexte scientifique

**La polymédication, une problématique néfaste pour le patient, notamment en soins palliatifs.**

La polymédication (définie par l’organisation mondiale de la santé comme l'administration de nombreux médicaments en même temps ou l'administration d'un nombre excessif de médicaments (1)) est fréquente chez les patients atteints d'une maladie grave et incurable potentiellement mortelle (2,3). Une récente revue de la littérature portant sur des études internationales a mis en évidence que trois à vingt-trois médicaments sont prescrits en moyenne dans cette population (3). Or, il a été démontré une association entre polymédication et diminution de la qualité de vie d’une part et augmentation des risques de iatrogénie (4) d’autre part, surtout chez cette population vulnérable, ce qui justifie de lutter contre ce phénomène.

**Encore trop de médicaments potentiellement inappropriés prescrits en soins palliatifs.**

Parmi les traitements prescrits, certains peuvent être considérés comme inappropriés pour les patients (PIMs, pour Potentially Inappropriate Medications = médicament potentiellement inapproprié) en raison de leur âge, de leur état de santé ou de leurs comorbidités, et peuvent entraîner des effets indésirables ou des interactions médicamenteuses potentiellement dangereuses. Ces prescriptions sont néanmoins fréquentes, la prévalence des patients en soins palliatifs ayant au moins une prescription potentiellement inappropriée variant de 15 à 92 % dans les études internationales (3).

**La déprescription, une nécessité.**

La déprescription est devenue ces dernières années un enjeu crucial dans de nombreuses spécialités. Elle correspond au processus de réduction ou d'arrêt de l'utilisation d'un médicament ou d'un traitement médicamenteux en cours, en raison de son inefficacité, de son caractère inapproprié, de ses effets indésirables, de ses interactions médicamenteuses potentiellement dangereuses ou de la préférence du patient. C'est une approche qui vise à optimiser la thérapie médicamenteuse en réduisant les risques et les coûts associés aux traitements médicamenteux inutiles ou inappropriés (2,5).

**Une nécessité aussi en soins palliatifs pour améliorer la qualité de vie de nos patients.**

Jusqu'à présent, la politique internationale de déprescription s'est concentrée principalement sur les personnes âgées, mais depuis quelques années une réflexion est en cours sur l'adaptation de cette politique à la population des soins palliatifs (4,6).

Cette réflexion est particulièrement menée chez les patients atteints de cancer qui sont plus à risque d’être polymédiqués (4,7,8) et dont les interactions médicamenteuses avec les traitements spécifiques du cancer sont fréquentes (9).

Ces actions de déprescriptions sont réalisées dans l'optique d'améliorer la qualité de vie et les coûts des soins de ces patients (10) qui ont une espérance de vie limitée.

**Une approche complexe en soins palliatifs.**

La déprescription en soins palliatifs oncologiques (c’est-à-dire pour des patients vivant avec un cancer avancé) est complexe et nécessite une approche individualisée en raison des risques de symptômes non contrôlés et d'effets indésirables liés à l'arrêt brutal de certains traitements. Ainsi, une planification minutieuse et une communication claire entre les différents professionnels de santé impliqués, le patient et ses proches sont nécessaires pour assurer une déprescription efficace, sécurisée, comprise et acceptée par chacun (11).

Nous devons continuer à développer la recherche dans ce domaine pour améliorer l’accompagnement de la déprescription en soins palliatifs qui fait partie intégrante des soins, par l’amélioration potentielle de la qualité de vie qui en découle (12).

# Justification de l’etude

La déprescription est une priorité de santé publique mais qui cumule des difficultés pour être mise en place en soins palliatifs oncologiques (13).

**Une absence de recommandations et d’organisation des équipes de soins.**

Il n’existe pas de recommandations sur la politique de déprescription en soins palliatifs, contrairement à d’autres populations (11). Alors même que les équipes de soins palliatifs n’ont pas été formées et organisées pour repérer la polymédication et ensuite l’aborder avec le patient pour questionner la déprescription (13). Pour autant, la situation de ces patients, particulièrement en oncologie, est souvent complexe comme on l’a vu précédemment avec notamment un grand nombre de médicaments en lien avec les symptômes variés qu’ils présentent (14).

**Des patients différents.**

Des outils pour aider le médecin (15) ont été construits mais, à l’image de la gériatrie, sans tenir compte de la spécificité de l'abord du patient en soins palliatifs (plus jeune, dans une dynamique de soins et une trajectoire de vie différentes) alors même que la littérature scientifique a démontré la nécessité « d'adopter une approche individualisée des prescriptions et exige (…) de reconsidérer la communication avec le patient » (12).

Il semble donc essentiel de connaitre et comprendre la perception de la déprescription par les patients en soins palliatifs avant même d’adapter des outils ou une quelconque politique de déprescription. Or, très peu d’études se sont questionnées sur les spécificités de cette perception dans cette population particulière (16,17). Les médecins eux-mêmes peuvent craindre ce que pourrait provoquer chez le patient, l’annonce d’un potentiel arrêt d’un traitement (13).

Il semble que les patients en soins palliatifs ne soient pas inquiets des conséquences de l’arrêt de leurs traitements préventifs (une seule étude portant l’arrêt des statines (16)). Cependant, nous ne savons pas ce que, potentiellement, nous pouvons provoquer en leur expliquant qu’un traitement préventif prescrit pour améliorer leur espérance de vie n’a plus lieu d’exister. Pour certains patients, il peut être difficile de questionner l’arrêt d’un traitement « prescrit à vie » (pouvant signifier pour lui jusqu’à la fin de sa vie) (17).

**Une nécessaire compréhension de la perception des patients.**

Dans ce contexte, nous devons mieux connaitre les différentes perceptions et enjeux que peut ressentir le patient lors d’un questionnement de déprescription, notamment d’un médicament potentiellement inapproprié pour individualiser chaque accompagnement.

Via une approche centrée sur le patient, l’étude que nous allons mener permettra de décrire, comprendre et expliquer les attitudes et croyances des patients autour de la déprescription de ces traitements, contribuant ainsi à une pratique clinique plus efficace et adaptée aux spécificités des soins palliatifs notamment oncologiques.

**Une équipe de recherche spécialisée dans l’évaluation et l’étude des perceptions des patients.**

A cet effet, nous allons mener une étude avec une équipe pluridisciplinaire (pharmacien, pharmacologue, psychologue, clinicien de soins palliatifs et méthodologiste) reconnus pour leurs recherches cliniques sur l’évaluation et l’étude des perceptions des patients, notamment en soins palliatifs. Ces membres émargent au sein de l’équipe INSERM UMR 1246 SPHERE (<https://sphere-inserm.fr/fr>), équipe dont « la philosophie est que le patient doit être considéré dans son ensemble, à savoir en prenant en compte son environnement, et en intégrant ses perceptions, son vécu et ses souhaits. »

Nous allons ainsi réaliser une étude multicentrique de méthodologie mixte séquentielle exploratoire : avec un recueil de données qualitatives qui permettra dans un premier temps de décrire les perceptions des patients à propos de la déprescription de médicaments potentiellement inappropriés, puis dans un second temps, un recueil de données quantitatives qui permettra de décrire la répartition des différentes perceptions dans la population et connaitre ce qui influence ces perceptions.

# Objectifs et criteres de jugement

## Objectif et critère d’évaluation principal

### Objectif principal

Étudier la perception de la déprescription qu’ont les patients en situation de soins palliatifs en cancérologie.

### Critère d’évaluation principal

Décrire, comprendre et expliquer les attitudes et croyances des patients concernant la déprescription en réalisant des entretiens individuels semi-dirigés (méthodologie d'approche descriptive avec analyse thématique réflexive) et dans un second temps à l’aide du questionnaire rPATD (questionnaire permettant d’évaluer la perception des patients concernant leurs traitements et leur déprescription) pouvant être adapté suite à l’analyse des données qualitatives.

## Objectifs et critères d’évaluation secondaires

### Objectif(s) secondaire(s)

1. Étudier les facteurs pouvant influencer les attitudes et croyances des patients concernant la dé-prescription.
2. Évaluer les propriétés psychométriques du rPATD dans cette population

### Critère(s) d’évaluation secondaire(s)

1. Influence du sexe, âge, niveau de diplôme, type de cancer, nombre de sites métastatiques, Score *Performans status*, présence d’un traitement systémique contre le cancer (chimiothérapie, thérapie ciblée, immunothérapie, hormonothérapie...), suivi par une équipe de soins palliatifs, lieu de soins du patient, personnes en charge du traitement, nombre de médicaments, nombre de « *potentially inappropriate medications* » (PIMs), pronostic évalué par le médecin référent, niveau de diplôme du patient et croyances sur les traitements en général (BMQ questionnaire) sur les scores issus du questionnaire rPATD
2. Description de données de validation du rPATD dans notre population : validation de la structure du questionnaire, mesure de la fiabilité des scores, validation concourante avec le questionnaire BMQ

## Étude Ancillaire :

Pour les patients qui le souhaitent (selon la fatigabilité de ces derniers), nous étudierons le lien entre le niveau de littératie en santé des patients et leur perception de la déprescription (la littératie en matière de santé est définie comme la capacité d'acquérir, de comprendre et d'utiliser des informations de manière à promouvoir et à maintenir une bonne santé (18)).

Pour se faire nous évaluerons le lien entre le niveau de littératie des patients (à l’aide de l’auto-questionnaire FCCHL (Functional, Communicative and Critical Health Literacy) / HLS14 (14-item health literacy scale) et leur perception de la déprescription.(en annexe 4)

# Population étudiée

## Description de la population

Nous nous intéresserons aux patients majeurs atteints d’un cancer « solide » en situation palliative (cancer localement avancé ou métastatique donc relevant de soins palliatifs selon la définition de l’Organisation mondiale de la santé(19)) et qui ont au moins un PIMs, suivis en hospitalisation ou en soins externes, ayant une espérance de vie estimée à moins de 1 an par le médecin prenant en charge le patient.

Pour repérer si un patient a au moins un PIMs, nous utiliserons l’outil STOPPFrail 2 (20) (en annexe 3). C’est un outil développé en gériatrie, validé pour une population avec une espérance de vie estimé de moins de 1 an et qui est non spécifique de l’oncologie. Dans notre population (soins palliatifs oncologiques), nous n’avons pas d’outil considéré de référence pour aider au repérage des PIMs (l’outil OncPal étant réservé au patient ayant moins de 6 mois d’espérance de vie)(21). L’espérance de vie de moins d’un an sera estimée par la question surprise par le médecin prenant en charge le patient (« seriez-vous surpris si votre patient décède dans l’année ? ») (22–24).

Nous réaliserons une étude multicentrique dans des services d’oncologies et/ou de soins palliatifs d’hôpitaux situés dans l’Ouest de la France.

Les centres ayant donné leur accord sont au nombre de 5 et couvrent les soins oncologiques de deux départements (la Loire-Atlantique et la Vendée) :

- Au sein du CHU de Nantes : service de soins palliatifs et soins de support, service d’oncologie médicale
- L’institut de cancérologie de l’ouest (CLCC), site de Saint-Herblain
- L’équipe mobile de soins palliatifs et de support du CH de Châteaubriant
- Le service de soins palliatifs du CH de Saint Nazaire
- Le service de soins palliatifs du CH de la Roche-sur-Yon

La participation à l’étude sera proposée par l’oncologue lors d’une consultation de suivi ou par le médecin en charge du patient si ce dernier est hospitalisé (que ce soit en hospitalisation conventionnelle ou en hôpital de jour).

Il y aura deux périodes d’inclusion différentes du fait design mixte séquentiel. Une première période qui correspondra au recueil de données qualitatives et s’étendra sur 8 mois ; une seconde (recueil de données quantitatives) sur 12 mois pour inclure les patients. Les deux périodes se succèderont, c’est-à-dire que l’analyse des données qualitatives pourra influencer la méthodologie de recueil des données quantitatives (les items des questionnaires seront potentiellement complétés par d’autres items suite à l’analyse des données qualitatives).

## Critères d’inclusion

- Patient de plus de 18 ans
- Atteint d’un cancer solide localement avancé ou métastatique (donc relevant de soins palliatifs selon la définition de l’Organisation mondiale de la santé)
- Espérance de vie estimée par le médecin à l’inclusion de moins de 1 an (utilisation de la question surprise pour aider le clinicien à estimer cette espérance de vie)
- Hospitalisé ou en consultation
- Ayant au moins un PIMs (repéré par l’utilisation de l’outil STOPPfrail 2)
- Patient n’ayant pas exprimé son opposition à participer à l’étude après avoir reçu l’information de la part du médecin.

**Pour les patients de l’étude qualitative :**

- Patient ayant signé l’autorisation de droit à l’enregistrement de leur voix au cour de l’entretient semi-structuré en vue d’une retranscription écrite

## Critères de non-inclusion

- Mineur
- Majeur sous tutelle, personne protégée
- Patient ne maitrisant pas la langue française orale et écrite
- Patient atteint de troubles du jugement, altération cognitive ou sensorielle importante ne permettant pas de recevoir une information éclairée ou de répondre aux questionnaires ou de participer à un entretien dans le cadre de l’étude.

# DESIGN ET Déroulement de l’étude

## Méthodologie générale de la recherche

La recherche présente les caractéristiques suivantes :

- Étude **multicentrique** nationale, **prospective, observationnelle,** de **design mixte séquentiel exploratoire**
  - Première partie qualitative : entretiens individuels semi dirigés avec méthodologie d’approche descriptive
  - Seconde partie quantitative : passation des questionnaires rPATD et BMQ et récolte de données socio-démographiques et médicales des patients.

### Description de la partie Qualitative

**La première partie de notre étude mixte sera une étude de méthodologie qualitative**.

Cette méthodologie a pour but d’étudier la perception des patients sans limiter les hypothèses dans une démarche inductive. Ainsi nous allons pouvoir percevoir les spécificités de notre population et créer des thèmes qui potentiellement n’ont pas été développés dans la littérature (25).

Nous faisons le choix de réaliser des **entretiens individuels semi-dirigés** avec une **méthodologie d'approche descriptive** (dont le but est de « décrire simplement un phénomène, une situation ou en événement dans son contexte») (25–27)**. Les données seront analysées en suivant les étapes de l'analyse thématique réflexive décrites par Braun et Clarke** (28–30). Nous avons fait le choix d’une méthodologie d’approche descriptive qui est « une méthode idéale pour décrire les expériences personnelles et les réponses des personnes à un évènement ou à une situation » (25–27).

Notre méthodologie est décrite ci-dessous. Nous nous sommes assurés de répondre à la grille COREQ : *COnsolidated criteria for REporting Qualitative research* (31), qui vise à vérifier que notre méthodologie qualitative est rigoureuse, et de répondre aux critères de qualité spécifiques à l’approche de Braun et Clarke (32).

Il a été décidé de **réaliser un échantillon de convenance (répondant aux critères d’inclusion et de non inclusion sus-cités) et diversifié** à minima en fonction du type de cancer primitif, des âges des patients et des sites hospitaliers de suivis. La taille de l’échantillon ne peut pas être déterminé au préalable selon Braun et Clarke (33). Malgré tout, la littérature nous permet d’émettre l’hypothèse d’un **recrutement de 25 patients afin d’obtenir une saturation des données.** Le recrutement des patients sera arrêté lorsque la saturation des données sera atteinte. Nous faisons le choix d'une saturation des thèmes avec une analyse au fil de l'eau des entretiens. Cette saturation des thèmes sera considérée comme atteinte lorsque l’analyse de deux entretiens successifs ne permettra pas d'identifier de nouveaux thèmes pertinents pour notre question de recherche.

Pour chaque patient inclus, il lui sera proposé de réaliser le jour même ou dans les 15 jours un **entretien semi-dirigé en tête à tête.** Si pour des raisons sanitaires ou des contraintes d’agendas, une rencontre physique ne peut avoir lieu, l’entretien se fera par visioconférence ou par téléphone. Un double enregistrement (2 dictaphones) numérisé de ces entretiens sera réalisé. Un journal de bord des chercheurs sera tenu lors de l’ensemble des entretiens afin de pouvoir noter des éléments perçus par le chercheur mais potentiellement non perceptibles sur les enregistrements, et d’apporter des informations qui pourront aider à interpréter les données en discussion. Ces éléments seront expliqués au patient à l’écrit par une note d’information et à l’oral par le médecin investigateur (oncologue ou médecin de soins palliatifs qui suit le patient) le jour de l’inclusion. Il lui sera stipulé que la recherche porte sur la perception des médicaments et notamment ceux qui sont potentiellement inappropriés. Il sera précisé les éléments réglementaires en lien avec la recherche (validation par un comité d’éthique locale, RGPD…décrit plus loin dans le paragraphe « aspects administratifs et réglementaires »).

Un seul entretien sera réalisé par participant. Il est estimé une durée moyenne des entretiens de 45 min (donné à titre indicatif pour permettre aux participants de pouvoir anticiper le temps nécessaire pour cet entretien). Notre population étant fragile, il apparait complexe de réaliser des focus groupes, c’est pourquoi cette méthode de collecte de données n’a pas été retenue.

**Les entretiens individuels seront réalisés par deux chercheurs**. Le chercheur principal, Adrien Evin, médecin de soins palliatifs, chercheur formé à la méthodologie qualitative et aux entretiens semi-dirigés et un second chercheur, pour les patients suivis par ce médecin, Pierre Nizet, pharmacien clinicien, formé aux méthodes qualitatives et aux entretiens semi-dirigés. Les deux chercheurs et l’ensemble de l’équipe scientifique du projet sont des cliniciens et chercheurs (pharmaciens, médecins ou psychologues) qui s’intéressent à l’approche centrée patient et au bon usage des médicaments.

Pour la réalisation de ces entretiens, les chercheurs utiliseront **un guide d’entretien** qui sert de support et aide le chercheur à aborder différents thèmes. Ce guide sera construit à partir des données de la littérature (17) issues d'études dans d’autres populations, et le fruit d'une réflexion et d'un travail de l’ensemble du comité scientifique. Pour s’assurer de la qualité de ce guide (à la fois en termes d’acceptabilité mais aussi de robustesse des thèmes abordés) il sera préalablement testé auprès de patients répondant aux critères d'inclusion de l'étude.

**Les entretiens seront retranscrits** intégralement en respectant de manière rigoureuse les propos du patient. Cette mission sera réalisée par un prestataire formé à la retranscription d’entretien dans le cadre de protocole de recherche.

Une **analyse thématique réflexive sera réalisée à partir de l’ensemble des données en suivant les six étapes de Braun et Clarke** (se familiariser avec l'ensemble de données, coder, créer des thèmes initiaux, développer et réviser les thèmes, affiner, définir et nommer les thèmes et enfin rédiger)**. De plus on adoptera une approche inductive** (*bottom-up*) (30) et un codage sémantique (descriptif). Un double codage sera réalisé et un comité scientifique sera consulté pour la création des thèmes. Le logiciel NVivo (déjà utilisé par le comité scientifique) sera utilisé pour cela.

### Description de la partie quantitative

Dans un second temps, après avoir réalisé les conclusions de l’étude qualitative, nous réaliserons une étude de méthodologie quantitative afin de savoir comment se répartissent les différents profils au sein de cette population et de connaitre les facteurs influençant la perception de la déprescription.

- Pour chaque patient inclus (correspondant aux patients remplissant après une information écrite et oral par le médecin les critères d’inclusion et n’ayant pas de critères non inclusion), **différentes données seront recueillies (un e-crf Ennov Clinical sera réalisé pour cette partie de l’étude):**
- Des données du dossier médical (recueillies par un attaché de recherche clinique) **:**

Sexe, Age (en année),Type de cancer (par grande localisation du primitif), Nombre de mois depuis le début du cancer, Nombre de sites métastatiques, Performance status (34) à l’inclusion, Présence de traitements systémiques contre le cancer (chimiothérapie, thérapie ciblée, immunothérapie, hormonothérapie...),Présence d’un suivi par équipe de soins palliatifs, Lieu de soins du patient (hospitalisation, domicile, EHPAD…).

- Des données recueillies auprès du médecin qui a inclus le patient par un formulaire papier :

- **Le nombre de médicaments** (nombre de molécules différentes prescrites et prises par le patient),

- **Le nombre de PIMs.** Pour cela le médecin utilisera l’outil STOPPfrail 2 (20) validé dans la population gériatrique avec une espérance de vie estimée à moins d’un an par le médecin. Il n’y a pas de grille de référence(15,21).

- Et en plus d’avoir estimé à l’inclusion le pronostic du patient à moins de un an, nous ferons estimer **le pronostic par le médecin pour la période moins de 6 mois et moins 3 mois par l’utilisation de la question surprise** (ex :« seriez-vous surpris si votre patient décède dans les 3 mois ? ») (22–24) afin d’estimer plus précisément le pronostic du patient (nous pourrons ainsi répartir notre échantillon en 3 groupes en fonction du pronostic estimé:<3 mois, <6 mois ou <12 mois).

- Des données recueillies auprès du patient par un formulaire papier :

- **Niveau du diplôme le plus élevé**,

- **Personne en charge de la gestion des médicaments** (par le patient lui-même, par un aidant, par un soignant)

- **Auto-questionnaire BMQ version française**, qui étudie la perception par le patient des médicaments (35) (en annexe 4).

Le *Beliefs about Medicine Questionnaire* (BMQ) est un auto-questionnaire qui explore en 18 items les représentations du patient. Il est validé en français, son utilisation est libre et gratuite.

Il est constitué de 10 items concernant les croyances spécifiques relatives aux traitements prescrits :

- la nécessité de prendre son traitement pour se maintenir en bonne santé (items 1, 3, 4, 7,10),

- les craintes portant sur les risques liés à son traitement (items 2, 5, 6, 8,9),

Et de huit items concernent les croyances générales :

- la notion de sur-utilisation des médicaments par les médecins (items 11, 14, 17, 18),

- la crainte d’un potentiel danger lié aux médicaments en général (items 12, 13, 15, 16).

Pour chaque item, une échelle de type Likert en 5 points est utilisée. Plus la somme des scores obtenus est élevée, plus la croyance du sujet est forte.

- **Auto-questionnaire rPATD** patient, version française (36) (en annexe 4)

Le rPATD est un questionnaire validé et adapté en français pour évaluer la perception des patients concernant leurs traitements et leur déprescription. Cet auto questionnaire est composé de 22 questions cotées par une échelle de Likert en 5 points.

Le questionnaire comprend deux questions sur la satisfaction globale à l'égard de l'utilisation des médicaments et la volonté d'accepter les recommandations de déprescription, ainsi que 20 questions regroupées en quatre facteurs validés : (I) la perception du fardeau de la médication (facteur Fardeau), (II) les attitudes à l'égard de la pertinence des médicaments prescrits (facteur Pertinence), (III) les préoccupations relatives à l'arrêt des médicaments (facteur Préoccupations relatives à l'arrêt), et (IV) le degré de connaissance des participants à l'égard de leurs médicaments et leur degré d'implication dans le processus de prise de décision concernant les médicaments (facteur Implication).

Il a été initialement utilisé en population gériatrique mais des récentes études l’utilisent chez le patient majeur quel que soit l’âge (37–39).

- En fonction des résultats de l’étude qualitative, **des items non présents dans l’auto questionnaire rPATD, pourront être ajoutés**.

- Pour les patients acceptant, un questionnaire supplémentaire (étude ancillaire), sera proposé : l’auto-questionnaire **FCCHL (Functional, Communicative and Critical Health Literacy) / HLS14 (14-item health literacy scale)** (40) (<https://reflis.fr/wp-content/uploads/2020/07/FCCHL-HLS14-Questionnaire-Litteratie-sante.pdf>)

Auto-questionnaire validé en français, libre d’utilisation et composé de14 items avec une échelle de Likert en 5 points permettant d’évaluer le niveau de littératie en santé (41):

- Littératie fonctionnelle : compétences de base suffisantes en lecture et en écriture pour pouvoir fonctionner efficacement dans les situations de tous les jours.

- Littératie communicative ou interactive : compétences cognitives et d'alphabétisation plus avancées qui, associées aux compétences sociales, peuvent être utilisées pour participer activement aux activités quotidiennes, extraire des informations et tirer des significations de différentes formes de communication, et appliquer de nouvelles informations aux circonstances changeantes.

- Littératie critique : compétences cognitives plus avancées qui, associées à des aptitudes sociales, peuvent être appliquées à l'analyse critique de l'information et à l'utilisation de cette information pour exercer un plus grand contrôle sur les événements et les situations de la vie.

- **Nombres de sujets à inclure :**

D’après la littérature, ce type de méthodologie quantitative descriptive est réalisée dans une population de patients assez nombreuse, généralement sur 300 à 400 patients (42,43). Il n’est pas évident (dans cette étude) de réaliser un calcul de nombre de sujets nécessaires car nous n’avons pas d’hypothèse principale à tester. Nous souhaitons cependant avoir suffisamment de données pour pouvoir réaliser des analyses afin de faire émerger de potentiels facteurs influençant avec une puissance suffisante.

**Nous envisageons ainsi 300 inclusions pour cette partie de l’étude**.

En effet, nous sommes en capacité de réaliser cette étude au vu des files actives de patients des différents centres et de la couverture par ces centres de deux départements. La prévalence des cancers et celle des PIMS étant des phénomènes fréquents (comme nous l’avons décrit dans notre introduction), nous devrions atteindre l’objectif d’inclusions fixé en 12 mois. L’ensemble de partenaires est formé aux bonnes pratiques de la recherche clinique et a l’habitude de participer à des études. Enfin, le comité scientifique fait partie des deux grands centres d’oncologie permettant de s’assurer du suivi du rythme des inclusions. Cette taille d’échantillon semble a priori suffisante pour obtenir une bonne puissance pour répondre à nos différents objectifs

- **Méthodes d’analyses (phase qualitative)**

1. **Afin de répondre à l’objectif principal** (Étudier la perception de la déprescription qu’ont les patients en situation de soins palliatifs en cancérologie.), nous décrirons la répartition des scores du rPATD (et des items non présents dans rPATD, potentiellement rajoutés) dans notre échantillon.
2. **Afin de répondre à l’objectif secondaire 1** (Étudier les facteurs pouvant influencer les attitudes et croyances des patients concernant la dé-prescription), nous utiliserons un modèle linéaire expliquant les scores du questionnaire rPATD par les différentes covariables recueillies (sexe, âge, niveau de diplôme, type de cancer, nombre de sites métastatiques, Score Performans status, présence d’un traitement systémique contre le cancer, suivi par une équipe de soins palliatifs, lieu de soins du patient, personnes en charge du traitement, nombre de médicaments, nombre de « potentially inappropriate medications » (PIMs), pronostic évalué par le médecin référent, niveau de diplôme du patient et croyances sur les traitements en général (scores BMQ))
3. **Afin de répondre à l’objectif secondaire 2** (Évaluer les propriétés psychométriques du rPATD dans cette population), nous réaliserons une analyse factorielle confirmatoire (CFA) sur les items du rPATD pour valider la structure (structure jugée cohérente si CFI>0.9 et RMSEA<0.08), nous estimerons la fiabilité par le coefficient alpha de Cronbach dans chaque dimension (bonne fiabilité si alpha >0.7) et nous estimerons la validité concourante en estimant les corrélations de Spearman entre les scores rPATD et les scores au questionnaire BMQ (bonne validité si des corrélations sont significatives)
4. **Afin de répondre à l’objectif de l’étude ancillaire** (étudier le lien entre le niveau de littératie en santé des patients et leur perception de la déprescription), nous calculerons les coefficients de corrélation de Spearman entre les scores rPATD et les scores BMQ (des corrélations significatives sont attendues)

## Calendrier de l’étude

L’étude sera réalisée sur trois ans.

Pour plus de clarté dans le calendrier de l’étude et les jalons de cette dernière, nous détaillerons dans un premier temps par phase d’étude pour ensuite présenter un calendrier global.

| **Détails de la phase qualitative**  **(8 mois d’inclusion des patients) :** | **Détails de la phase quantitative**  **(12 mois d’inclusion des patients) :** |
| --- | --- |
| \| **Actions** \| **J0** \| **entre J0 à J15** \| \| --- \| --- \| --- \| \| Information \| x \|  \| \| Recueil de non opposition \| x \|  \| \| Recueil d’autorisation de droit à la voix \| x \|  \| \| Recueil des coordonnées (mail et téléphone) \| x \|  \| \| Recueil des données du dossier médical \| x \|  \| \| Recueil des éléments précisés par le médecin \| x \|  \| \| Réalisation de l'entretien \|  \| x \| \| Recueil des données patients \|  \| x \| | \| **Actions** \| **J0** \| \| --- \| --- \| \| Information \| x \| \| Recueil de non opposition \| x \| \| Recueil des données du dossier médical \| x \| \| Recueil des données patients : Questionnaire rPATD , BMQ et autres questions patients \| x \| \| Recueil des éléments précisés par le médecin \| x \| \| Recueil Questionnaire Littéracie (étude ancillaire) \| x \| |

**Calendrier global des 3 ans**


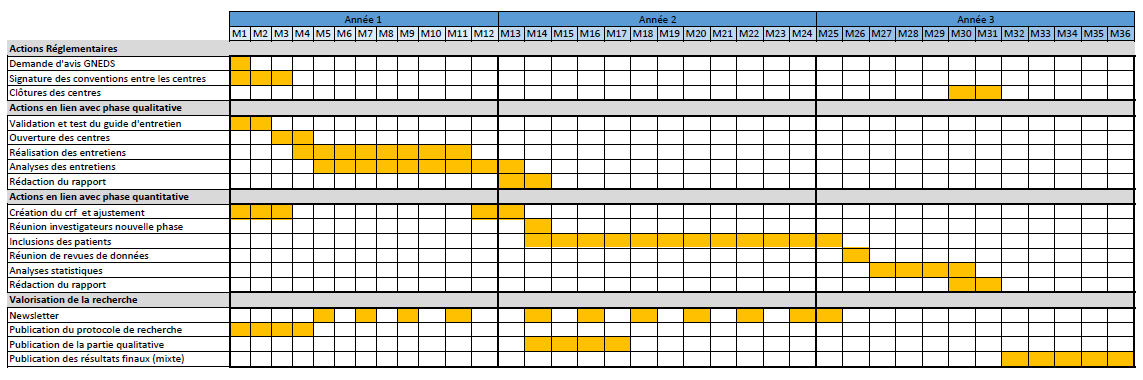


**Faisabilité du calendrier**

La faisabilité du calendrier de l’étude a été étudiée par le comité scientifique afin d’être cohérent avec les objectifs. L’investigateur coordinateur du projet a un temps de 20 % dédié pour ce projet. Le comité scientifique est pluridisciplinaire (une des richesses de ce projet) et est expérimenté en recherche clinique (deux d’entre eux travaillent pour des directions de la recherche de leur établissement). Il est soudé et fait partie d’une même équipe de recherche INSERM. L’ensemble des membres de ce comité sont universitaires et publient très régulièrement dans des revues indexées internationales. Ce projet sera aussi soutenu par des travaux d’étudiants en master 2 (les membres du comité scientifique étant enseignants dans plusieurs masters). Pour avoir un nombre suffisant de sujet à inclure, il a été fait le choix d’une étude multicentrique. La sélection des centres a été faite en fonction des liens qui existent entre eux. Ce qui va permettre une dynamique forte d’inclusion (staff mensuel en place depuis 2 ans entre les centres). Ils sont tous à proximité de Nantes et, pour les deux plus grands centres, des membres du comité scientifique y travaillent régulièrement. Afin de s’assurer de la dynamique et du suivi rigoureux des inclusions, une coordinatrice d’étude clinique (infirmière de recherche clinique habituée aux recherches sur la fin de vie) sera impliquée. Enfin, du temps médical et du temps de technicien de recherche clinique sont également prévus au budget pour permettre une saisie de données de qualité et de s’assurer que les centres maintiennent un rythme d’inclusion en adéquation avec les objectifs de l’étude.

# Aspects administratifs et réglementaires

Nous sommes dans une étude en sciences humaines et sociales dans le domaine de la santé, observationnelle sans risque pour le patient. Nous sommes donc hors loi Jardé. Nous solliciterons avant de débuter l’étude l’avis du comité d’éthique locale Nantais (GNEDS Groupe Nantais d'Éthique dans le Domaine de la Santé).

Dans le cadre de la réglementation en cours, l’investigateur s’engage à informer le patient de façon claire et juste du protocole (note d’information écrite de non opposition à la recherche en plus de l’information orale). Il remettra au patient un exemplaire de la note d’information. Celle-ci précisera la possibilité pour le patient de refuser de participer à la recherche et de se retirer à tout moment.

Au vue des retours concernant l’utilisation dans des lettres d’information de précédentes études du terme « patients en situation palliative », le comité scientifique a décidé d’utiliser le terme « patient vivant avec un cancer avancé ». En effet, le terme « palliatif » est mal compris encore aujourd’hui dans la population et est synonyme de fin de vie imminente ce qui n’est pas le cas du terme « cancer avancé ». Ce choix vise à éviter que le patient par cette lettre d’information ou par cette étude se méprenne sur son pronostic vital.

Une autorisation de droit à la voix sera recueillie auprès des patients qui participeront à la partie qualitative afin de pouvoir réaliser les enregistrements des entretiens semi dirigés.

L’accord écrit du patient à être recontacté par un médecin du CHU de Nantes sera également recueilli.

L’investigateur notera dans le dossier du patient que celui-ci a bien été informé oralement, a reçu la note d’information et a donné son accord écrit pour participer à la recherche ainsi que pour l’utilisation de ses données pour la recherche; il datera cette information.

Le traitement des données sera enregistré dans le registre RGPD du CHU de Nantes.

Les données recueillies au cours de l’étude seront conservées dans un fichier informatique respectant la loi « informatique et libertés » du 6 janvier 1978 modifiée, la loi n° 2018-493 du 20 juin 2018 relative à la protection des données personnelles et le Règlement (UE) 2016/679 du Parlement européen et du Conseil du 27 avril 2016 relatif à la protection des personnes physiques à l'égard du traitement des données à caractère personnel et à la libre circulation de ces données (RGPD).Elles seront donc pseudo-anonymisées avec une codification respectant la réglementation.

Le protocole entre dans le champ de la Méthodologie de référence MR004 à laquelle se conforme le CHU de Nantes qui a l’autorisation de la CNIL.

# retombées attendues au plan scientifique et en termes de santé publique

- Les résultats de cette étude permettront d’**améliorer les connaissances** dans le domaine du bon usage des médicaments et plus particulièrement de la **lutte contre la polymédication** à travers la déprescription. La perception de la déprescription chez les patients en situation palliative oncologique sera mieux appréhendée, afin de **pouvoir au mieux adapter les outils mais aussi les discours/positionnements des soignants sur la déprescription**. L’objectif est de **diminuer la polymédication à terme dans cette population, permettant ainsi d’améliorer la qualité de vie des patients et de diminuer les coûts des soins** (10).

- L’étude permettra de mieux comprendre les liens/associations pouvant exister entre les perceptions des patients sur la déprescription et des éléments d’ordre médical, socio démographique ou encore de littératie. Ainsi, **les soignants pourront au mieux s’adapter aux éléments spécifiques du patient.**

- L’étude ancillaire sur la littératie en santé de cette population, nous permettra de mieux **appréhender à la fois le niveau de littératie de nos patients et de réfléchir aux actions à mettre en place pour potentiellement l’améliorer**. Pour rappel, en santé publique de nos jours « améliorer le niveau de littératie est un enjeu majeur de santé publique pour que la population soit en capacité de prendre en charge au mieux sa santé. (…) Un effort important de recherche au niveau international doit être entrepris pour hausser le niveau de littératie du plus grand nombre ».( <https://www.santepubliquefrance.fr/docs/la-litteratie-en-sante-un-concept-critique-pour-la-sante-publique> )

- Indirectement cette étude va aussi **modifier probablement les pratiques des centres en sensibilisant les médecins à la question des PIMs et de la déprescription en soins palliatifs**. Des **formations à destination des professionnels de santé** pourront être organisées à partir de nos résultats sur les thématiques de la polymédication et de la déprescription en soins palliatifs.

- Cette étude sera aussi le début d’un projet, qui sera poursuivi après par **la mise en œuvre d’actions de déprescription adaptées à cette population (en évaluant de manière plus spécifique les différents domaines de qualité de vie des patients potentiellement impactés, les coûts en santé…**).

Liste des annexes

- ANNEXE 1 : Références bibliographiques
- ANNEXE 2 : Listing des intervenants principaux
- ANNEXE 3 : Questionnaires
- ANNEXE 4 : Note d’information patient et autorisation de droit à la voix et à l’image
- ANNEXE 5 : Engagement de conformité à la MR004

ANNEXE 1: RÉFÉRENCES BIBLIOGRAPHIQUES

1. WHO Centre for Health Development (Kobe J. A glossary of terms for community health care and services for older persons [Internet]. Kobe, Japan: WHO Centre for Health Development; 2004 [cité 26 avr 2023]. Report No.: WHO/WKC/Tech.Ser./04.2. Disponible sur: https://apps.who.int/iris/handle/10665/68896

2. Scott IA, Hilmer SN, Reeve E, Potter K, Le Couteur D, Rigby D, et al. Reducing inappropriate polypharmacy: the process of deprescribing. JAMA Intern Med. mai 2015;175(5):827‑34.

3. Cadogan CA, Murphy M, Boland M, Bennett K, McLean S, Hughes C. Prescribing practices, patterns, and potential harms in patients receiving palliative care: A systematic scoping review. Explor Res Clin Soc Pharm. sept 2021;3:100050.

4. Schenker Y, Park SY, Jeong K, Pruskowski J, Kavalieratos D, Resick J, et al. Associations Between Polypharmacy, Symptom Burden, and Quality of Life in Patients with Advanced, Life-Limiting Illness. J Gen Intern Med. avr 2019;34(4):559‑66.

5. Reeve E, Gnjidic D, Long J, Hilmer S. A systematic review of the emerging deﬁnition of « deprescribing » with network analysis: implications for future research and clinical practice. Br J Clin Pharmacol. déc 2015;80(6):1254‑68.

6. Morin L, Wastesson JW, Laroche ML, Fastbom J, Johnell K. How many older adults receive drugs of questionable clinical benefit near the end of life? A cohort study. Palliat Med. sept 2019;33(8):1080‑90.

7. Lees J, Chan A. Polypharmacy in elderly patients with cancer: clinical implications and management. Lancet Oncol. déc 2011;12(13):1249‑57.

8. Todd A, Al-Khafaji J, Akhter N, Kasim A, Quibell R, Merriman K, et al. Missed opportunities: unnecessary medicine use in patients with lung cancer at the end of life - an international cohort study. Br J Clin Pharmacol. déc 2018;84(12):2802‑10.

9. Blower P, de Wit R, Goodin S, Aapro M. Drug–drug interactions in oncology: Why are they important and can they be minimized? Crit Rev Oncol Hematol. 1 août 2005 [cité 26 avr 2023];55(2):117‑42.

10. Kutner JS, Blatchford PJ, Taylor DH, Ritchie CS, Bull JH, Fairclough DL, et al. Safety and Benefit of Discontinuing Statin Therapy in the Setting of Advanced, Life-Limiting Illness. JAMA Intern Med. mai 2015;175(5):691‑700.

11. Meyer-Junco L. Time to Deprescribe: A Time-Centric Model for Deprescribing at End of Life. J Palliat Med. févr 2021;24(2):273‑84.

12. Tjia J, Karakida M, Alcusky M, Furuno JP. Perspectives on deprescribing in palliative care. Expert Rev Clin Pharmacol. 19 avr 2023;1‑11.

13. Paque K, Vander Stichele R, Elseviers M, Pardon K, Dilles T, Deliens L, et al. Barriers and enablers to deprescribing in people with a life-limiting disease: A systematic review. Palliat Med. janv 2019;33(1):37‑48.

14. Harris D. Safe and effective prescribing for symptom management in palliative care. Br J Hosp Med Lond Engl 2005. 2 déc 2019;80(12):C184‑9.

15. Van Merendonk LN, Crul M. Deprescribing in palliative patients with cancer: a concise review of tools and guidelines. Support Care Cancer Off J Multinatl Assoc Support Care Cancer. avr 2022;30(4):2933‑43.

16. Tjia J, Kutner JS, Ritchie CS, Blatchford PJ, Bennett Kendrick RE, Prince-Paul M, et al. Perceptions of Statin Discontinuation among Patients with Life-Limiting Illness. J Palliat Med. oct 2017;20(10):1098‑103.

17. Todd A, Holmes H, Pearson S, Hughes C, Andrew I, Baker L, et al. « I don’t think I’d be frightened if the statins went »: a phenomenological qualitative study exploring medicines use in palliative care patients, carers and healthcare professionals. BMC Palliat Care. 29 janv 2016;15:13.

18. Sørensen K, Van den Broucke S, Fullam J, Doyle G, Pelikan J, Slonska Z, et al. Health literacy and public health: a systematic review and integration of definitions and models. BMC Public Health. 25 janv 2012;12:80.

19. Soins palliatifs. OMS [Internet]. [cité 26 avr 2023]. Disponible sur: https://www.who.int/fr/news-room/fact-sheets/detail/palliative-care

20. Curtin D, Gallagher P, O’Mahony D. Deprescribing in older people approaching end-of-life: development and validation of STOPPFrail version 2. Age Ageing. 26 févr 2021;50(2):465‑71.

21. McNeill R, Hanger HC, Chieng J, Chin P. Polypharmacy in Palliative Care: Two Deprescribing Tools Compared with a Clinical Review. J Palliat Med. mai 2021;24(5):661‑7.

22. Hui D, Paiva CE, Del Fabbro EG, Steer C, Naberhuis J, van de Wetering M, et al. Prognostication in advanced cancer: update and directions for future research. Support Care Cancer Off J Multinatl Assoc Support Care Cancer. juin 2019;27(6):1973‑84.

23. White N, Kupeli N, Vickerstaff V, Stone P. How accurate is the « Surprise Question » at identifying patients at the end of life? A systematic review and meta-analysis. BMC Med. 2 août 2017;15(1):139.

24. White N, Reid F, Harris A, Harries P, Stone P. A Systematic Review of Predictions of Survival in Palliative Care: How Accurate Are Clinicians and Who Are the Experts? PloS One. 2016;11(8):e0161407.

25. Fortin MF, Gagnon J. Fondements et étapes du processus de recherche. 4e édition. CHENELIERE; 2022. 496 p.

26. Sandelowski M. Whatever happened to qualitative description? Res Nurs Health. août 2000;23(4):334‑40.

27. Sandelowski M. What’s in a name? Qualitative description revisited. Res Nurs Health. févr 2010;33(1):77‑84.

28. Braun V, Clarke V. Using thematic analysis in psychology. Qual Res Psychol . 1 janv 2006;3(2):77‑101.

29. Braun V, Clarke V. Reflecting on reflexive thematic analysis. Qual Res Sport Exerc Health. 8 août 2019;11(4):589‑97.

30. Braun V, Clarke V. Conceptual and design thinking for thematic analysis. Qual Psychol. 2022;9:3‑26.

31. Tong A, Sainsbury P, Craig J. Consolidated criteria for reporting qualitative research (COREQ): a 32-item checklist for interviews and focus groups. Int J Qual Health Care J Int Soc Qual Health Care. déc 2007;19(6):349‑57.

32. Braun V, Clarke V. One size fits all? What counts as quality practice in (reflexive) thematic analysis? Qual Res Psychol . 3 juill 2021;18(3):328‑52.

33. Braun V, Clarke V. To saturate or not to saturate? Questioning data saturation as a useful concept for thematic analysis and sample-size rationales. Qual Res Sport Exerc Health. 4 mars 202;113(2):201‑16.

34. Oken MM, Creech RH, Tormey DC, Horton J, Davis TE, McFadden ET, et al. Toxicity and response criteria of the Eastern Cooperative Oncology Group. Am J Clin Oncol. déc 1982;5(6):649‑55.

35. Fall E, Gauchet A, Izaute M, Horne R, Chakroun N. Validation of the French version of the Beliefs about Medicines Questionnaire (BMQ) among diabetes and HIV patients. Eur Rev Appl Psychol. 1 nov 2014;64(6):335‑43.

36. Roux B, Sirois C, Niquille A, Spinewine A, Ouellet N, Pétein C, et al. Cross-cultural adaptation and psychometric validation of the revised Patients’ Attitudes Towards Deprescribing (rPATD) questionnaire in French. Res Soc Adm Pharm RSAP. août 2021;17(8):1453‑62.

37. Gaurang N, Priyadharsini R, Balamurugesan K, Prakash M, Reka D. Attitudes and beliefs of patients and primary caregivers towards deprescribing in a tertiary health care facility. Pharm Pract. 2021;19(3):2350.

38. Lukacena KM, Keck JW, Freeman PR, Harrington NG, Huffmyer MJ, Moga DC. Patients’ attitudes toward deprescribing and their experiences communicating with clinicians and pharmacists. Ther Adv Drug Saf. 2022;13:20420986221116464.

39. Nguyen-Soenen J, Rat C, Gaultier A, Schirr-Bonnans S, Tessier P, Fournier JP. Effectiveness of a multi-faceted intervention to deprescribe proton pump inhibitors in primary care: protocol for a population-based, pragmatic, cluster-randomized controlled trial. BMC Health Serv Res. 17 févr 2022;22(1):219.

40. Ousseine YM, Rouquette A, Bouhnik AD, Rigal L, Ringa V, Smith A « Ben », et al. Validation of the French version of the Functional, Communicative and Critical Health Literacy scale (FCCHL). J Patient-Rep Outcomes. 2017;2(1):3.

41. Nutbeam D. Health literacy as a public health goal: a challenge for contemporary health education and communication strategies into the 21st century. Health Promot Int . 1 sept 2000 ;15(3):259‑67.

42. Crutzen S, Abou J, Smits SE, Baas G, Hugtenburg JG, Heringa M, et al. Older people’s attitudes towards deprescribing cardiometabolic medication. BMC Geriatr. 16 juin 2021;21(1):366.

43. Roux B, Rakheja B, Sirois C, Niquille A, Pétein C, Ouellet N, et al. Attitudes and beliefs of older adults and caregivers towards deprescribing in French-speaking countries: a multicenter cross-sectional study. Eur J Clin Pharmacol. 1 oct 2022;78(10):1633‑46.

ANNEXE 2 : LISTING DES INTERVENANT PRINCIPAUX

| **NOM ET PRENOM** | **Spécialité**  **& Fonction** | **Nom de l’établissement** | **Nom du service de rattachement** | **Rôle dans l’étude** |
| --- | --- | --- | --- | --- |
| Adrien EVIN | MCU-PH (médecine palliative) | Nantes Université et CHU de Nantes | Service Interdisciplinaire Douleur, Soins Palliatifs et de Support, Médecine intégrative | Investigateur principal |
| André COLPAERT | PH (médecine palliative) | CH CNP et CHU Nantes | Service Interdisciplinaire Douleur, Soins Palliatifs et de Support, Médecine intégrative et Unité mobile de soins palliatifs CH CNP | Investigateur |
| Véronique BARBAROT | Praticien des centre (oncologue/médecine palliative) | ICO site Nantes | Service d’oncologie médicale | Investigateur |
| Yann TOUCHEFEU | PU-PH (gastroentérologue-oncologue) | CHU Nantes | unité pluridisciplinaire d'oncologie médicale | Investigateur |
| Elvire PONS-TOSTIVIN | MCU-PH | CHU Nantes | Oncologie Thoracique | Investigateur |
| Gaëlle QUEREUX BAUMGARTNER | MCU-PH | CHU Nantes | Onco-Dermatologie | Investigateur |
| Virginie DESSUS-CHEVREL | PH (médecine palliative) | CH ST Nazaire | Unité de soins palliatifs | Investigateur |
| Caroline HENNION | PH (médecine palliative) | CHD Vendée | Unité de soins palliatifs | Investigateur |

ANNEXE 3 : QUESTIONNAIRES

*
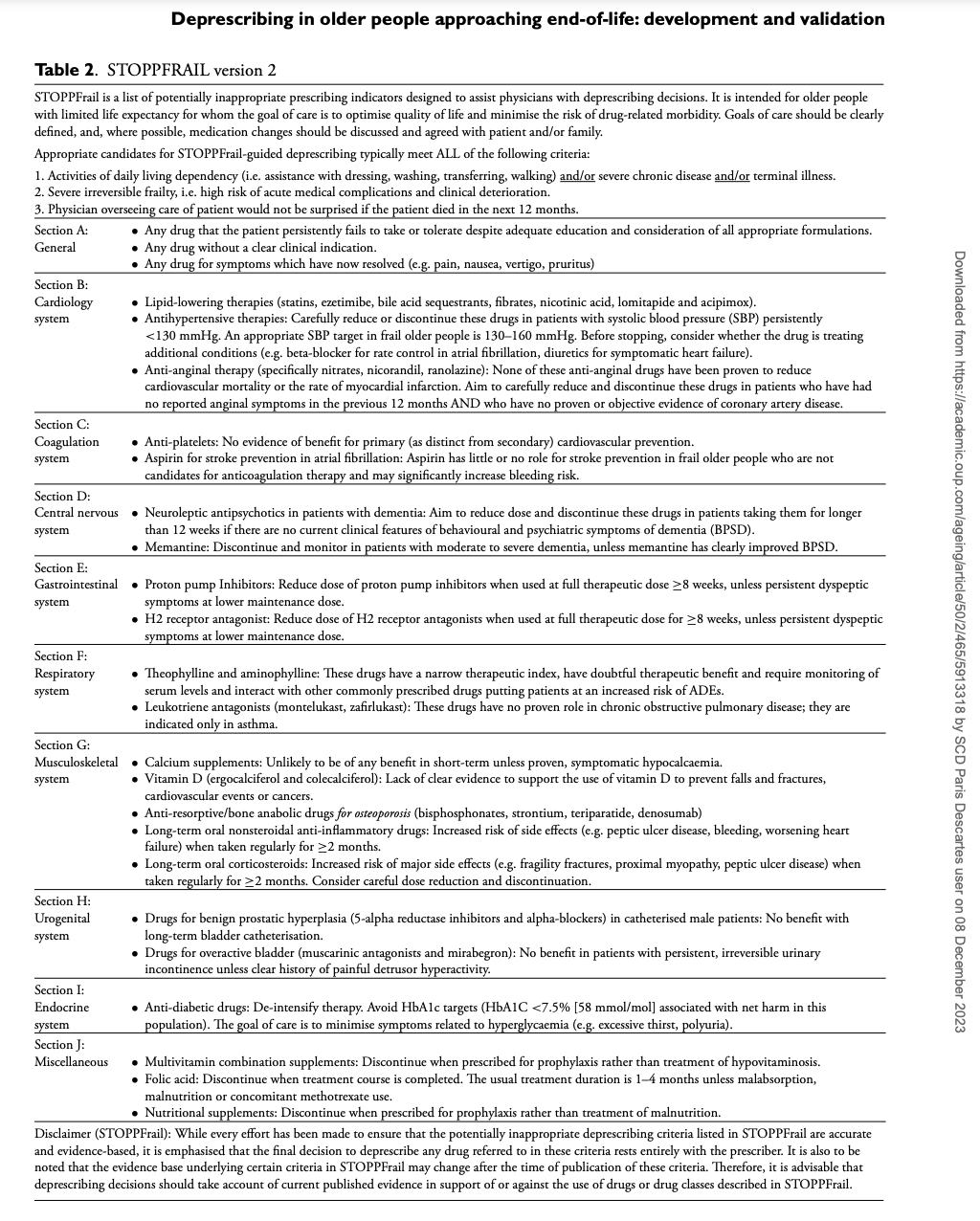
*

***French version of the Beliefs about Medicines Questionnaire.***

Nous voudrions vous poser des questions sur votre point de vue personnel concernant les traitements qui vous sont prescrits. Les déclarations suivantes ont été faites par d'autres personnes concernant leurs médicaments.

**• S'il vous plaît, indiquer dans quelle mesure vous êtes en accord ou en désaccord avec eux en faisant une croix dans la case appropriée (réponse qui vous correspond le plus).**

**• Il n'y a pas de bonnes ou de mauvaises réponses.**

**Nous sommes intéressés par vos opinions personnelles**

| Tout à fait  d’accord | D’accord | Incertain | En  désaccord | Fortement en désaccord |
| --- | --- | --- | --- | --- |

**Croyances spécifiques :**

1. Ma santé, aujourd’hui, dépend de mon traitement.

2. Avoir à prendre un traitement m’inquiète.

3. Ma vie serait impossible sans mon traitement.

4. Sans mon traitement, je serais très malade.

5. Je m’inquiète parfois à propos des effets à long terme de mon traitement.

6. Mon traitement est un mystère pour moi.

7. Ma santé future dépend de mon traitement.

8. Mon traitement perturbe ma vie.

9. Je suis parfois inquiet(e) de devenir trop dépendant(e) de mon traitement.10. Mon traitement empêche mon état d’empirer.

**Croyances générales** :

11. Les médecins utilisent trop de traitements.

12. Les personnes qui prennent des médicaments devraient arrêter leur traitement de temps en temps.

13. La plupart des traitements provoquent une dépendance.

14. Les remèdes naturels sont plus sûrs que les traitements médicaux.

15. Les traitements font plus de mal que de bien.

16. Tous les traitements sont des poisons.

17. Les médecins accordent trop de confiance aux traitements.

18. Si les médecins passaient plus de temps avec les patients, ils prescriraient moins de traitements.

***rPATD version française***

• S'il vous plaît, indiquer dans quelle mesure vous êtes en accord ou en désaccord avec ces propositions en mettant une croix dans la case appropriée (réponse qui vous correspond le plus).

• Il n'y a pas de bonnes ou de mauvaises réponses.

| Tout à fait  d’accord | D’accord | Incertain | En  désaccord | Fortement en désaccord |
| --- | --- | --- | --- | --- |

| Q1 | Je dépense beaucoup d’argent pour mes médicaments |
| --- | --- |
| Q2 | Prendre mes médicaments tous les jours n’est pas très pratique |
| Q3 | Je trouve que je prends un grand nombre de médicaments |
| Q4 | Je trouve que mes médicaments sont une contrainte pour moi |
| Q5 | Parfois, je pense que je prends trop de médicaments |
| Q6 | Je trouve que je prends peut-être un ou plusieurs médicaments dont je n’ai plus besoin |
| Q7 | J’aimerais essayer d’arrêter un de mes médicaments pour voir comment je me sentirais sans celui-ci |
| Q8 | J’aimerais que mon médecin réduise la dose d’un ou plusieurs de mes médicaments |
| Q9 | Je pense qu’un ou plusieurs de mes médicaments ne sont peut-être pas efficaces |
| Q10 | Je crois qu’un ou plusieurs de mes médicaments peuvent me donner en ce moment des effets indésirables |
| Q11 | Je serais réticent(e) à arrêter un médicament que je prends depuis longtemps |
| Q12 | Si un de mes médicaments était arrêté, je serais inquiet(e) de passer à côté de ses futurs bénéfices |
| Q13 | Je suis inquiet(e)/stressé(e) chaque fois que mes médicaments sont changés |
| Q14 | Si mon médecin me recommandait d’arrêter un médicament, j’aurais le sentiment qu’il renonce à me soigner. |
| Q15 | J’ai déjà eu une mauvaise expérience quand un médicament a été arrêté |
| Q16 | Je comprends bien pourquoi on m’a prescrit chacun de mes médicaments |
| Q17 | Je sais exactement quels médicaments je prends en ce moment et/ou je tiens à jour une liste de médicaments |
| Q18 | J’aime en savoir le plus possible sur mes médicaments |
| Q19 | J’aime être impliqué(e) avec mes médecins dans les décisions qui sont prises concernant mes médicaments |
| Q20 | Je demande toujours au médecin, au pharmacien ou à un autre professionnel de la santé s’il y a quelque chose que je ne comprends pas au sujet de mes médicaments |
| Q21 | Si mon médecin disait que cela est possible, je serais prêt(e) à arrêter un ou plusieurs de mes médicaments habituels |
| Q22 | Globalement, je suis satisfait(e) de mes médicaments actuels |

***FCCHL (FUNCTIONAL, COMMUNICATIVE AND CRITICAL HEALTH LITERACY) / HLS14 (14-ITEM HEALTH LITERACY SCALE)***


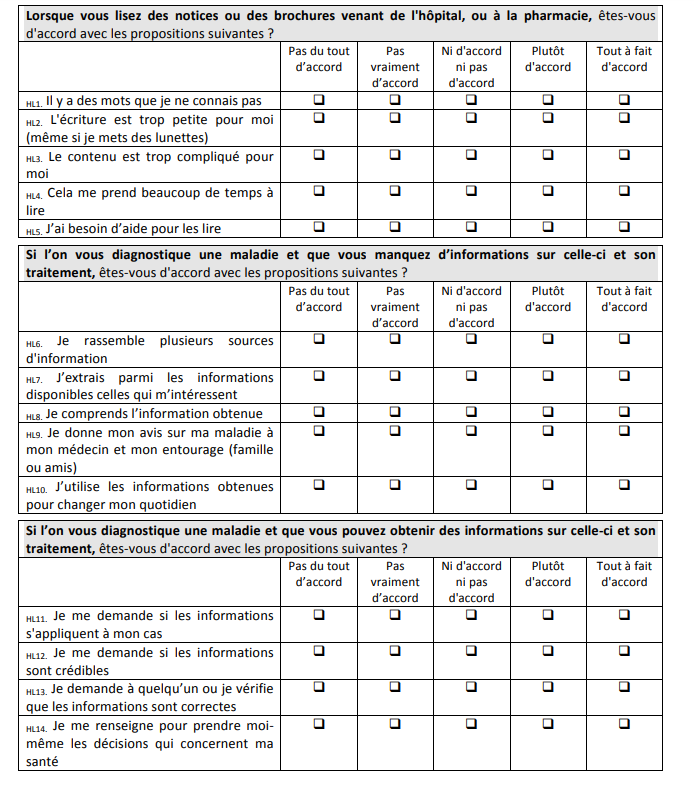


ANNEXE 4 : NOTES D’INFORMATION PATIENT


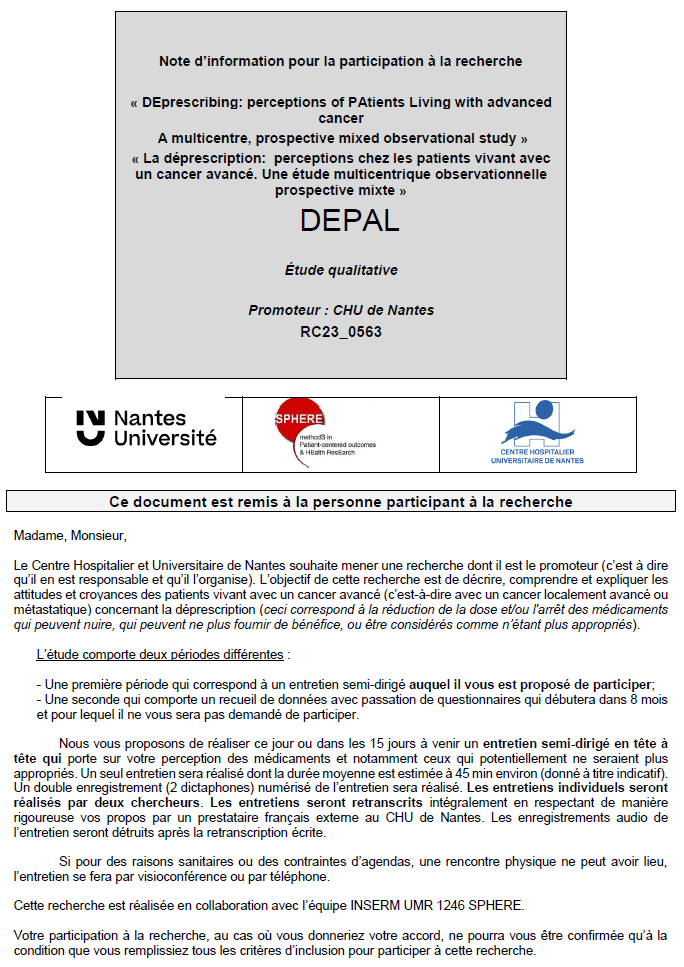


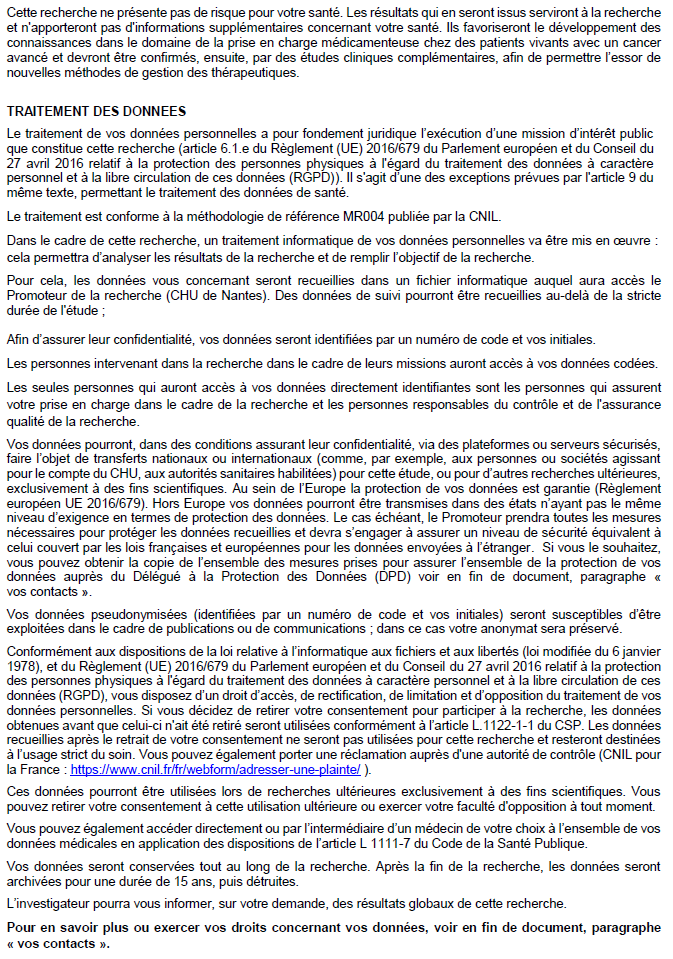


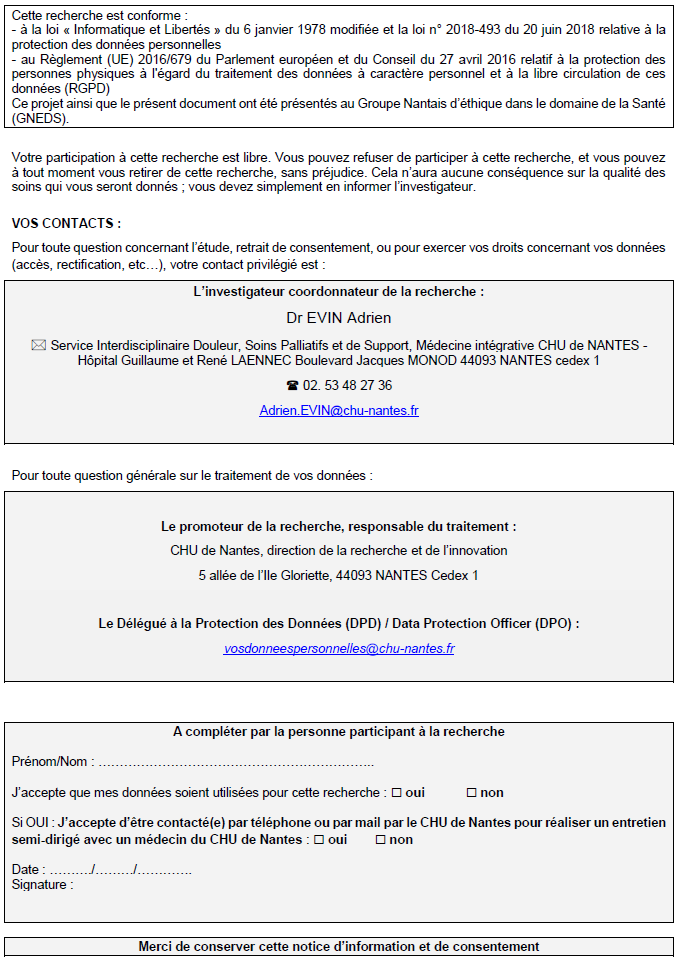


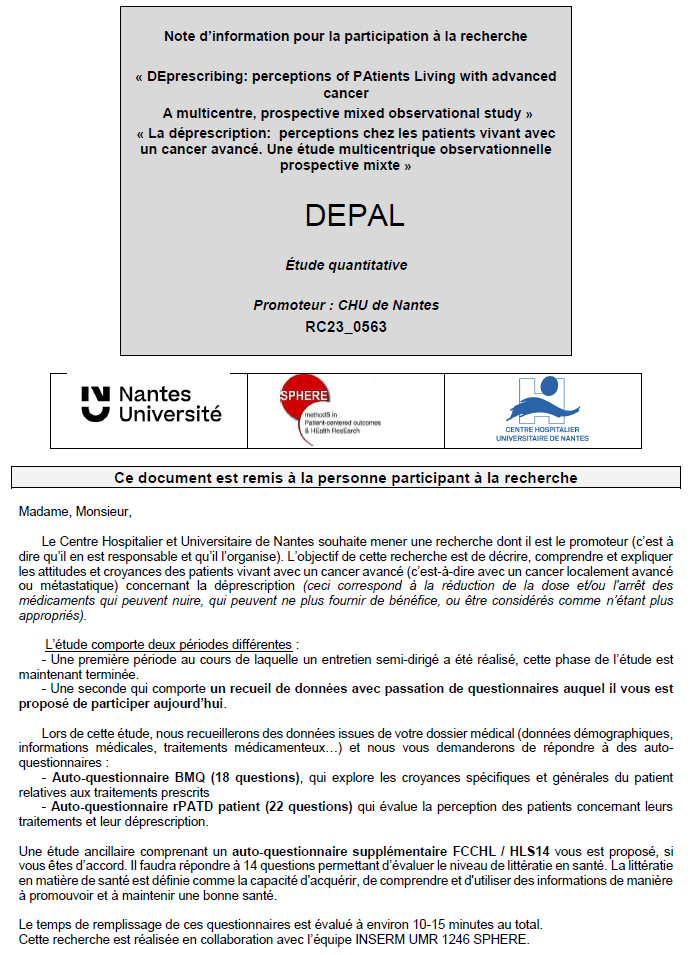


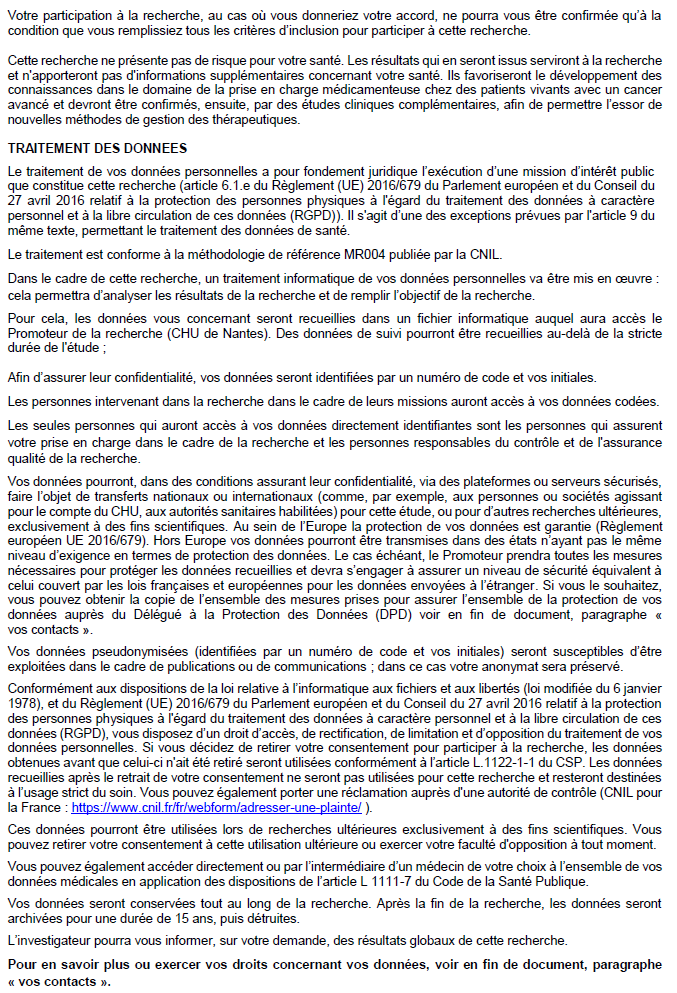


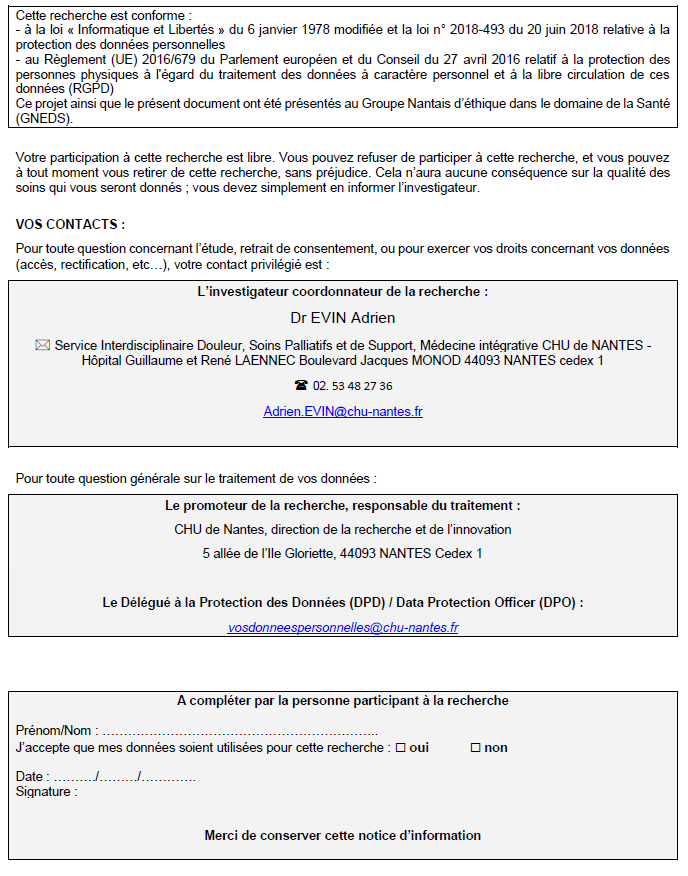


ANNEXE 5 : AUTORISATION DE DROIT A LA VOIX


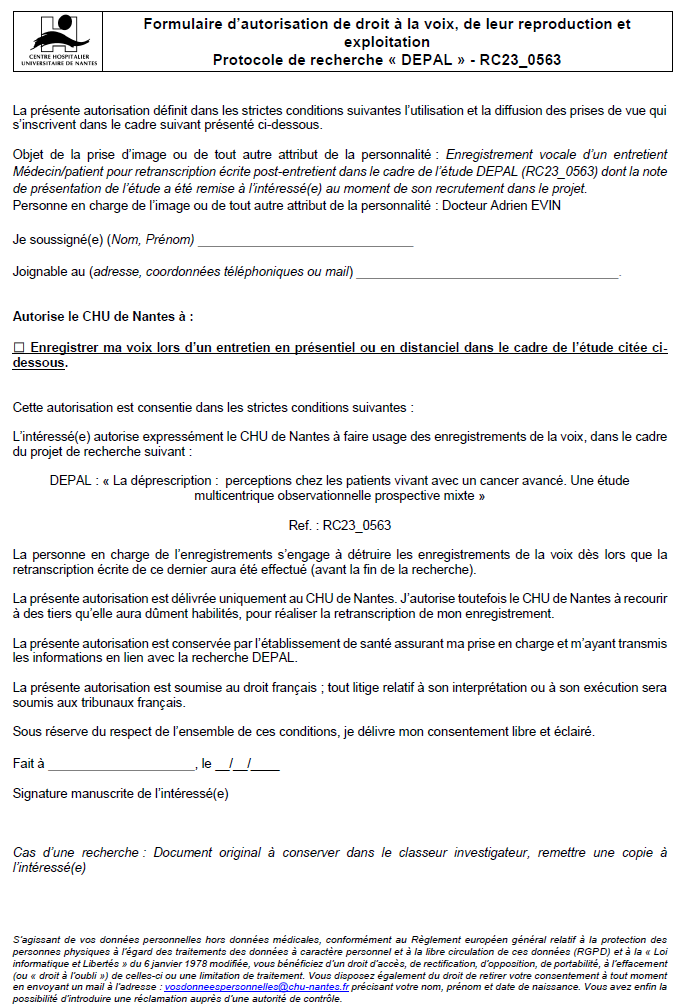


ANNEXE 6 : Engagement de conformité à la MR004


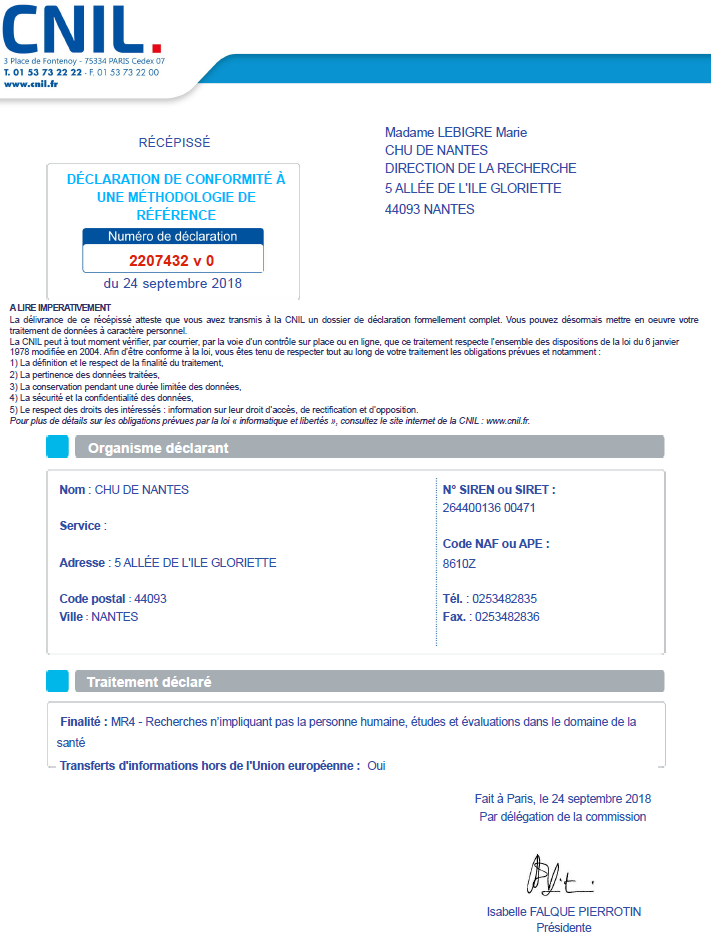

Supplement: S8 File — (DOCX) [file pone.0305737.s009.docx]
