# Supplementary material for: DEprescribing: Perceptions of PAtients living with advanced cancer. A multicentre, prospective mixed observational study protocol
Source: PLoS One. 2024 Aug 20;19(8):e0305737. doi: 10.1371/journal.pone.0305737 (PMC11335145; doi:10.1371/journal.pone.0305737)

***DEPAL study protocol***

**“** **DEprescribing: perceptions of PAtients Living with advanced cancer.** **A multicenter, prospective mixed observational study. »**

**Ref. : RC23_0563**

**Coordinating Investigator:**

**Dr Adrien EVIN**

Hospital Practitioner (MD, PhD student)

Palliative Care Unit (5th floor, south wing)

Interdisciplinary Pain Service, Palliative and Supportive Care, Integrative Medicine

NANTES University Hospital - Guillaume and René LAENNEC Hospital

Boulevard Jacques MONOD

44093 NANTES cedex 1

INSERM UMR 1246 SPHERE and UIC 22

Tel 02 53 48 27 36 [Adrien.evin@chu-nantes.fr](mailto:Adrien.evin@chu-nantes.fr)

**Methodologist:**

**Jean-Benoit Hardouin**

Lecturer - Hospital Practitioner, PhD

Public Health Service - Methodology and Biostatistics Platform - Nantes University Hospital

INSERM UMR 1246-SPHERE

[jean-benoit.hardouin@univ-nantes.fr](mailto:jean-benoit.hardouin@univ-nantes.fr)

**Scientific committee:**

**Marianne Bourdon**

Clinical psychologist, PhD

Western Cancer Institute, Nantes, Angers, France

INSERM UMR 1246 SPHERE

**Dr Jean-François HUON**

Lecturer - Hospital Practitioner, PharmD, PhD

Clinical Pharmacy & Public Health, Faculty of Pharmacy of Nantes, University Hospital of Nantes

INSERM UMR 1246 SPHERE

**Dr Pierre Nizet**

University hospital assistant, PharmD, PhD student

Clinical Pharmacy & Public Health, Faculty of Pharmacy of Nantes, University Hospital of Nantes

INSERM UMR 1246 SPHERE

**Professor Caroline Victorri-Vigneau**

PU-PH, PharmD, PhD, HDR

Department of Clinical Pharmacology, Faculty of Medicine, Nantes University Hospital

INSERM UMR 1246 SPHERE

**Promoter:**
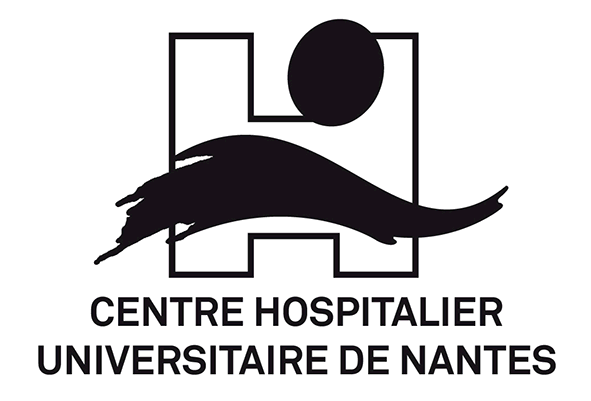
**Nantes University Hospital**


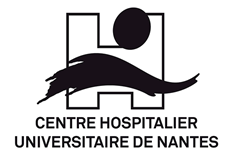
Medical Affairs Department

and Research
5, allée de l’île Gloriette
44 093 Nantes cedex 01 (FRANCE)

[Bp-prom-regl@chu-nantes.fr](mailto:Bp-prom-regl@chu-nantes.fr)

Tel: 02 53 48 28 35
Fax: 02 53 48 28 36

***Contents***

[1.](#_Toc161245794)  [INTRODUCTION/Scientific context](#_Toc161245794)  [3](#_Toc161245794)

[2.](#_Toc161245795)  [Rationale for study](#_Toc161245795)  [3](#_Toc161245795)

[3.](#_Toc161245796)  [Objectives and judgment criteria](#_Toc161245796)  [4](#_Toc161245796)

[3.1.](#_Toc161245797)  [Objective and primary endpoint](#_Toc161245797)  [4](#_Toc161245797)

[3.2.](#_Toc161245798)  [Secondary objectives and endpoints](#_Toc161245798)  [5](#_Toc161245798)

[3.3.](#_Toc161245799)  [Ancillary Study :](#_Toc161245799)  [5](#_Toc161245799)

[4.](#_Toc161245800)  [Population studied](#_Toc161245800)  [5](#_Toc161245800)

[4.1.](#_Toc161245801)  [Description of population](#_Toc161245801)  [5](#_Toc161245801)

[4.2.](#_Toc161245802)  [Inclusion criteria](#_Toc161245802)  [6](#_Toc161245802)

[4.3.](#_Toc161245803)  [Non-inclusion criteria](#_Toc161245803)  [6](#_Toc161245803)

[5.](#_Toc161245804)  [DESIGN AND Conduct of the study](#_Toc161245804)  [6](#_Toc161245804)

[5.1.](#_Toc161245805)  [General research methodology](#_Toc161245805)  [6](#_Toc161245805)

[5.2.](#_Toc161245806)  [Study Schedule](#_Toc161245806)  [10](#_Toc161245806)

[6.](#_Toc161245807)  [Administrative and regulatory aspects](#_Toc161245807)  [12](#_Toc161245807)

[7.](#_Toc161245808)  [expected scientific and public health benefits](#_Toc161245808)  [12](#_Toc161245808)

[List of annexes](#_Toc161245809)  [13](#_Toc161245809)

[APPENDIX 1: BIBLIOGRAPHICAL REFERENCES](#_Toc161245810)  [14](#_Toc161245810)

[ANNEX 2: LIST OF MAIN SPEAKERS](#_Toc161245811)  [16](#_Toc161245811)

[APPENDIX 3: QUESTIONNAIRES](#_Toc161245812)  [17](#_Toc161245812)

[APPENDIX 4: PATIENT INFORMATION NOTES](#_Toc161245813)  [21](#_Toc161245813)

[APPENDIX 5: AUTHORIZATION OF RIGHT TO VOICE](#_Toc161245814)  [27](#_Toc161245814)

[ANNEX 6: Commitment to compliance with MR004](#_Toc161245815)  [28](#_Toc161245815)

# INTRODUCTION /Scientific context

**Polypharmacy, a harmful problem for the patient, particularly in palliative care.**

Polypharmacy (defined by the World Health Organization as the administration of many medications at the same time or the administration of an excessive number of medications (1) ) is common among patients with serious, incurable illness. potentially fatal (2,3) . A recent literature review of international studies highlighted that three to twenty-three medications are prescribed on average in this population (3) . However, an association has been demonstrated between polypharmacy and a reduction in quality of life on the one hand and an increase in the risk of iatrogenics (4) on the other hand, especially in this vulnerable population, which justifies fighting against this phenomenon.

**Still too many potentially inappropriate medications prescribed for palliative care.**

Among the treatments prescribed, some may be considered inappropriate for patients (PIMs, for Potentially Inappropriate Medications = potentially inappropriate medication) due to their age, their state of health or their comorbidities, and can lead to adverse effects or potentially dangerous drug interactions. These prescriptions are nevertheless common, with the prevalence of palliative care patients having at least one potentially inappropriate prescription varying from 15 to 92% in international studies (3) .

**Deprescribing, a necessity.**

In recent years, deprescribing has become a crucial issue in many specialties. It corresponds to the process of reducing or stopping the use of a medication or drug treatment in progress, due to its ineffectiveness, its inappropriate nature, its adverse effects, its potentially dangerous drug interactions or of patient preference. It is an approach that aims to optimize drug therapy by reducing the risks and costs associated with unnecessary or inappropriate drug treatments (2,5) .

**A necessity also in palliative care to improve the quality of life of our patients.**

Until now, international deprescribing policy has focused mainly on the elderly, but for several years consideration has been underway on the adaptation of this policy to the palliative care population (4,6) .

This reflection is particularly carried out in cancer patients who are more at risk of being polymedicated (4,7,8) and whose drug interactions with specific cancer treatments are frequent (9) .

These deprescribing actions are carried out with the aim of improving the quality of life and the costs of care of these patients (10) who have a limited life expectancy.

**A complex approach to palliative care.**

Deprescribing in oncological palliative care (i.e. for patients living with advanced cancer) is complex and requires an individualized approach due to the risks of uncontrolled symptoms and adverse effects linked to abrupt cessation of certain treatments. Thus, careful planning and clear communication between the different healthcare professionals involved, the patient and their loved ones are necessary to ensure effective, safe deprescribing, understood and accepted by everyone (11) .

We must continue to develop research in this area to improve support for deprescribing in palliative care, which is an integral part of care, through the potential improvement in quality of life that results from it (12) .

# Rationale of the study

Deprescribing is a public health priority but one that faces difficulties in being implemented in oncological palliative care (13) .

**A lack of recommendations and organization of care teams.**

There are no recommendations on deprescribing policy in palliative care, unlike in other populations (11) . Even though palliative care teams have not been trained and organized to identify polypharmacy and then discuss it with the patient to question deprescription (13) . However, the situation of these patients, particularly in oncology, is often complex as we have seen previously, notably with a large number of medications linked to the varied symptoms they present (14) .

**Different patients.**

Tools to help the doctor (15) have been built but, like geriatrics, without taking into account the specificity of approaching the patient in palliative care (younger, in a dynamic of care and a trajectory of different lives) even though the scientific literature has demonstrated the need “to adopt an individualized approach to prescriptions and requires (…) to reconsider communication with the patient” (12) .

It therefore seems essential to know and understand the perception of deprescribing by patients in palliative care before even adapting tools or any deprescribing policy. However, very few studies have questioned the specificities of this perception in this particular population (16,17) . Doctors themselves may fear what the announcement of a potential cessation of treatment could cause in the patient (13) .

It seems that palliative care patients are not worried about the consequences of stopping their preventive treatments (only one study involving stopping statins (16) ). However, we do not know what, potentially, we can cause by explaining to them that a preventive treatment prescribed to improve their life expectancy no longer exists. For some patients, it can be difficult to question stopping a treatment “prescribed for life” (which could mean for them until the end of their life) (17) .

**A necessary understanding of patient perception.**

In this context, we must better understand the different perceptions and issues that the patient may feel when questioning deprescription, particularly of a potentially inappropriate medication, in order to individualize each support.

Via a patient-centered approach, the study we are going to conduct will make it possible to describe, understand and explain patients' attitudes and beliefs around the deprescription of these treatments, thus contributing to more effective clinical practice adapted to the specificities of palliative care, particularly oncology.

**A research team specializing in the evaluation and study of patient perceptions.**

To this end, we will conduct a study with a multidisciplinary team (pharmacist, pharmacologist, psychologist, palliative care clinician and methodologist) recognized for their clinical research on the evaluation and study of patient perceptions, particularly in palliative care. These members emerge within the INSERM UMR 1246 SPHERE team ( <https://sphere-inserm.fr/fr>), a team whose “ philosophy is that the patient must be considered as a whole, namely by taking into account their environment, and by integrating their perceptions, their experiences and their wishes. »

We will therefore carry out a multicenter study of exploratory sequential mixed methodology: with a collection of qualitative data which will firstly allow us to describe patients' perceptions about the deprescription of potentially inappropriate medications, then secondly, a collection of quantitative data which will make it possible to describe the distribution of different perceptions in the population and to know what influences these perceptions.

# Objectives and judgment criteria

## Objective and primary endpoint

### Primary objective

To study the perception of deprescribing by patients in palliative care situations in oncology.

### Primary endpoint

Describe, understand and explain patients' attitudes and beliefs regarding deprescribing by carrying out individual semi-structured interviews (descriptive approach methodology with reflective thematic analysis) and secondly using the rPATD questionnaire (questionnaire allowing evaluate patients' perceptions regarding their treatments and their deprescription) which can be adapted following the analysis of qualitative data.

## Secondary Objectives and Endpoints

### Secondary objective(s)

1. To study the factors that may influence patients' attitudes and beliefs regarding de-prescription.
2. Evaluate the psychometric properties of the rPATD in this population

### Secondary endpoint(s)

1. Influence of sex, age, level of education, type of cancer, number of metastatic sites, Score *Performans status* , presence of systemic treatment against cancer (chemotherapy, targeted therapy, immunotherapy, hormonal therapy, etc.), followed by a palliative care team, patient's place of care, people in charge of treatment, number of medications, number of “ *potentially inappropriate medications* ” (PIMs), prognosis assessed by the referring doctor, patient's educational level and beliefs about treatments in general (BMQ questionnaire) on the scores from the rPATD questionnaire
2. Description of rPATD validation data in our population: validation of the questionnaire structure, measurement of score reliability, concurrent validation with the BMQ questionnaire

## Ancillary Study :

For patients who wish it (depending on their fatigue), we will study the link between patients' level of health literacy and their perception of deprescribing (health literacy is defined as the ability to acquire, understand and use information in ways that promote and maintain good health (18)).

To do this, we will evaluate the link between the level of literacy of patients (using the self-questionnaire FCCHL (Functional, Communicative and Critical Health Literacy) / HLS14 (14-item health literacy scale) and their perception of deprescription.(in appendix 4)

# Population studied

## Description of the population

We will focus on adult patients with “solid” cancer in a palliative situation (locally advanced or metastatic cancer, therefore requiring palliative care according to the definition of the World Health Organization (19) ) and who have at least one PIMs , followed in hospitalization or outpatient care, with a life expectancy estimated at less than 1 year by the doctor caring for the patient.

To identify whether a patient has at least one PIMs, we will use the STOPPFrail 2 tool (20) (in appendix 3). It is a tool developed in geriatrics, validated for a population with an estimated life expectancy of less than 1 year and which is not specific to oncology. In our population (palliative oncological care), we do not have a reference tool to help identify PIMs (the OncPal tool is reserved for patients with less than 6 months of life expectancy) (21) . Life expectancy of less than one year will be estimated by the surprise question by the doctor caring for the patient (“would you be surprised if your patient died within one year?”) (22–24) .

We will carry out a multicenter study in oncology and/or palliative care departments of hospitals located in the West of France.

There are 5 centers that have given their agreement and cover oncological care in two departments (Loire-Atlantique and Vendée):

- Within the Nantes University Hospital: palliative care and supportive care service, medical oncology service
- The Western Cancer Institute (CLCC), Saint-Herblain site
- The mobile palliative care and support team at Châteaubriant Hospital
- The palliative care service at the Saint Nazaire CH
- The palliative care service at the CH de la Roche-sur-Yon

Participation in the study will be proposed by the oncologist during a follow-up consultation or by the doctor in charge of the patient if the latter is hospitalized (whether in conventional hospitalization or in a day hospital).

There will be two different inclusion periods due to the sequential mixed design. A first period which will correspond to the collection of qualitative data and will extend over 8 months; a second (quantitative data collection) over 12 months to include patients. The two periods will follow one another, that is to say that the analysis of the qualitative data may influence the methodology for collecting the quantitative data (the items in the questionnaires will potentially be supplemented by other items following the analysis of the qualitative data ).

## Inclusion criteria

- Patient over 18 years old
- Suffering from locally advanced or metastatic solid cancer (therefore requiring palliative care according to the definition of the World Health Organization)
- Life expectancy estimated by the doctor at inclusion of less than 1 year (use of the surprise question to help the clinician estimate this life expectancy)
- Hospitalized or in consultation
- Having at least one PIMs (identified by using the STOPPfrail 2 tool)
- Patient who did not express his opposition to participating in the study after having received the information from the doctor.

**For patients in the qualitative study:**

- Patient having signed the authorization for the right to record their voice during the semi-structured interview for written transcription

## Non-inclusion criteria

- Minor
- Adult under guardianship, protected person
- Patient not mastering the oral and written French language
- Patient suffering from impaired judgment, significant cognitive or sensory impairment which prevents him from receiving informed information or answering questionnaires or participating in an interview as part of the study.

# DESIGN AND Conduct of the study

## General research methodology

The research has the following characteristics:

- National, **prospective, observational, exploratory sequential mixed** design **multicenter** study
  - First qualitative part: semi-directed individual interviews with descriptive approach methodology
  - Second quantitative part: administration of the rPATD and BMQ questionnaires and collection of socio-demographic and medical data from patients.

### Description of the Qualitative part

**The first part of our mixed study will be a qualitative methodology study** .

This methodology aims to study patients' perceptions without limiting hypotheses in an inductive approach. Thus we will be able to perceive the specificities of our population and create themes which potentially have not been developed in the literature (25) .

We choose to carry out **semi-structured individual interviews** with a **descriptive approach methodology** (the aim of which is to “simply describe a phenomenon, a situation or an event in its context”) (25–27) **. Data will be analyzed following the steps of reflective thematic analysis described by Braun and Clarke** (28–30) . We have chosen a descriptive approach methodology which is “an ideal method for describing personal experiences and people's responses to an event or situation” (25–27) .

Our methodology is described below. We made sure to respond to the COREQ grid: *COnsolidated criteria for REporting Qualitative research* (31) , which aims to verify that our qualitative methodology is rigorous, and to respond to the quality criteria specific to the Braun and Clarke approach (32). ) .

It was decided to **carry out a convenience sample (meeting the aforementioned inclusion and non-inclusion criteria) and diversified** at least according to the type of primary cancer, the ages of the patients and the follow-up hospital sites. The sample size cannot be determined in advance according to Braun and Clarke (33) . Despite everything, the literature allows us to hypothesize the **recruitment of 25 patients in order to obtain data saturation.** Patient recruitment will be stopped when data saturation is reached. We choose a saturation of themes with an analysis of the interviews over time. This saturation of themes will be considered to have been reached when the analysis of two successive interviews does not make it possible to identify new themes relevant to our research question.

**semi-directed face-to-face interview** on the same day or within 15 days . If for health reasons or scheduling constraints, a physical meeting cannot take place, the interview will be done by videoconference or by telephone. A double digitized recording (2 dictaphones) of these interviews will be made. A researchers' logbook will be kept during all the interviews in order to be able to note elements perceived by the researcher but potentially not perceptible on the recordings, and to provide information which can help interpret the data under discussion. These elements will be explained to the patient in writing by an information note and orally by the investigating doctor (oncologist or palliative care doctor who follows the patient) on the day of inclusion. It will be stipulated that the research focuses on the perception of medications and particularly those that are potentially inappropriate. The regulatory elements linked to the research will be specified (validation by a local ethics committee, GDPR, etc. described further in the “administrative and regulatory aspects” paragraph).

Only one interview will be carried out per participant. An average interview duration of 45 minutes is estimated (given as an indication to allow participants to anticipate the time needed for this interview). Our population being fragile, it appears complex to carry out focus groups, which is why this method of data collection was not chosen.

**Individual interviews will be carried out by two researchers** . The main researcher, Adrien Evin, palliative care doctor, researcher trained in qualitative methodology and semi-structured interviews and a second researcher, for the patients followed by this doctor, Pierre Nizet, clinical pharmacist, trained in qualitative methods and interviews semi-directed. The two researchers and the entire scientific team of the project are clinicians and researchers (pharmacists, doctors or psychologists) who are interested in the patient-centered approach and the proper use of medications.

To carry out these interviews, the researchers will use **an interview guide** which serves as support and helps the researcher to address different themes. This guide will be constructed from literature data (17) from studies in other populations, and the fruit of reflection and work by the entire scientific committee. To ensure the quality of this guide (both in terms of acceptability but also robustness of the themes covered) it will be tested beforehand with patients meeting the study inclusion criteria.

**The interviews will be transcribed** in full, strictly respecting the patient's words. This mission will be carried out by a service provider trained in interview transcription as part of the research protocol.

A **reflective thematic analysis will be conducted on the data set following Braun and Clarke's six steps** (becoming familiar with the data set, coding, creating initial themes, developing and revising themes, refining, defining and name the themes and finally write) **. In addition, we will adopt an inductive** ( *bottom-up* ) approach (30) and semantic (descriptive) coding. Double coding will be carried out and a scientific committee will be consulted for the creation of the themes. The NVivo software (already used by the scientific committee) will be used for this.

### Description of the quantitative part

Secondly, after having drawn up the conclusions of the qualitative study, we will carry out a quantitative methodology study in order to know how the different profiles are distributed within this population and to know the factors influencing the perception of deprescription.

- For each patient included (corresponding to patients fulfilling the inclusion criteria after written and oral information from the doctor and not having non-inclusion criteria), **different data will be collected (an Ennov Clinical e-crf will be carried out for this part of the study):**
- Data from the medical file (collected by a clinical research associate) **:**

Sex, Age (in years), Type of cancer (by major location of the primary), Number of months since the start of the cancer, Number of metastatic sites, Performance status (34) at inclusion, Presence of systemic treatments against cancer (chemotherapy, targeted therapy, immunotherapy, hormonal therapy, etc.), Presence of monitoring by a palliative care team, Patient's place of care (hospitalization, home, EHPAD, etc.).

- Data collected from the doctor who included the patient via a paper form :

- **The number of medications** (number of different molecules prescribed and taken by the patient),

- **The number of PIMs.** For this, the doctor will use the STOPPfrail 2 tool (20) validated in the geriatric population with a life expectancy estimated at less than one year by the doctor. There is no reference grid (15,21) .

- And in addition to having estimated the patient's prognosis at less than one year at inclusion, we will have **the prognosis estimated by the doctor for the period less than 6 months and less than 3 months by using the surprise question** ( ex: “would you be surprised if your patient died within 3 months?”) (22–24) in order to more precisely estimate the patient's prognosis (we will thus be able to divide our sample into 3 groups according to the estimated prognosis:< 3 months, <6 months or <12 months).

- Data collected from the patient via a paper form :

- **Highest level of diploma** ,

- **Person in charge of medication management** (by the patient himself, by a caregiver, by a caregiver)

- **BMQ self-questionnaire French version** , which studies the patient's perception of medications (35) (in appendix 4).

The *Beliefs about Medicine Questionnaire* (BMQ) is a self-questionnaire which explores the patient's representations in 18 items. It is validated in French, its use is free and unrestricted.

It is made up of 10 items concerning specific beliefs relating to prescribed treatments:

- the need to take treatment to maintain good health (items 1, 3, 4, 7,10),

- fears relating to the risks linked to its treatment (items 2, 5, 6, 8.9),

And eight items concern general beliefs:

- the notion of overuse of medications by doctors (items 11, 14, 17, 18),

- fear of potential danger linked to medications in general (items 12, 13, 15, 16).

For each item, a 5-point Likert-type scale is used. The higher the sum of the scores obtained, the stronger the subject's belief.

- **rPATD patient self-questionnaire** , French version (36) (in appendix 4)

The rPATD is a validated questionnaire adapted in French to assess patients' perceptions regarding their treatments and deprescription. This self-questionnaire is made up of 22 questions rated on a 5-point Likert scale.

The questionnaire includes two questions on overall satisfaction with medication use and willingness to accept deprescribing recommendations, as well as 20 questions grouped into four validated factors: (I) perception of medication burden (Burden factor), (II) attitudes toward the appropriateness of prescribed medications (Relevance factor), (III) concerns about stopping medications (Concerns About Stopping factor), and (IV ) the participants' degree of knowledge about their medications and their degree of involvement in the decision-making process regarding medications (Involvement factor).

It was initially used in the geriatric population but recent studies use it in adult patients regardless of age (37–39) .

- Depending on the results of the qualitative study, **items not present in the rPATD self-questionnaire may be added** .

- For accepting patients, an additional questionnaire (ancillary study) will be offered: the **FCCHL (Functional, Communicative and Critical Health Literacy) / HLS14 (14-item health literacy scale) self-questionnaire** (40) ( <https://reflis.fr/wp-content/uploads/2020/07/FCCHL-HLS14-Questionnaire-Litteratie-sante.pdf>)

Self-questionnaire validated in French, free to use and composed of 14 items with a 5-point Likert scale to assess the level of health literacy (41) :

- Functional literacy: sufficient basic reading and writing skills to be able to function effectively in everyday situations.

- Communicative or interactive literacy: more advanced cognitive and literacy skills that, combined with social skills, can be used to actively participate in daily activities, extract information and derive meanings from different forms of communication, and apply new information to changing circumstances.

- Critical literacy: more advanced cognitive skills that, combined with social skills, can be applied to critically analyzing information and using that information to exert greater control over events and situations in the life.

- **Number of topics to include:**

According to the literature, this type of descriptive quantitative methodology is performed in a fairly large patient population, generally on 300 to 400 patients (42,43) . It is not easy (in this study) to calculate the number of subjects needed because we do not have a main hypothesis to test. However, we would like to have sufficient data to be able to carry out analyzes in order to bring out potential influencing factors with sufficient power.

**We are therefore considering 300 inclusions for this part of the study** .

Indeed, we are able to carry out this study in view of the active queues of patients from the different centers and the coverage by these centers of two departments. The prevalence of cancers and that of PIMS being frequent phenomena (as we described in our introduction), we should achieve the inclusion objective set within 12 months. All partners are trained in good clinical research practices and are used to participating in studies. Finally, the scientific committee is part of the two major oncology centers making it possible to monitor the rate of inclusions. This sample size seems a priori sufficient to obtain good power to meet our different objectives.

- **Analysis methods (qualitative phase)**

1. **In order to meet the main objective** (To study the perception of deprescribing by patients in palliative care situations in oncology.), we will describe the distribution of rPATD scores (and items not present in rPATD, potentially added) in our sample.
2. **In order to respond to secondary objective 1** ( Study the factors that can influence patients' attitudes and beliefs regarding de-prescription), we will use a linear model explaining the scores of the rPATD questionnaire by the different covariates collected ( sex, age, level degree, type of cancer, number of metastatic sites, Score Performans status, presence of systemic treatment against cancer, followed by a palliative care team, place of care of the patient, people in charge of treatment, number of medications, number of “potentially inappropriate medications” (PIMs), prognosis assessed by the referring physician, patient's educational level and beliefs about treatments in general (BMQ scores))
3. **In order to respond to secondary objective 2** ( Evaluate the psychometric properties of the rPATD in this population), we will carry out a confirmatory factor analysis (CFA) on the items of the rPATD to validate the structure (structure deemed coherent if CFI>0.9 and RMSEA< 0.08), we will estimate the reliability by the Cronbach's alpha coefficient in each dimension (good reliability if alpha >0.7) and we will estimate the concurrent validity by estimating the Spearman correlations between the rPATD scores and the scores on the BMQ questionnaire (good validity if correlations are significant)
4. **In order to meet the objective of the ancillary study** ( to study the link between the level of health literacy of patients and their perception of deprescription), we will calculate the Spearman correlation coefficients between the rPATD scores and the FCCHL /HLS14 ( significant correlations are expected)

## Study Schedule

The study will be carried out over three years.

For greater clarity in the study schedule and milestones, we will first detail each study phase and then present an overall schedule.

| **Details of the qualitative phase**  **(8 months of patient inclusion):** | **Details of the quantitative phase**  **(12 months of patient inclusion):** |
| --- | --- |
| \| **Actions** \| **J0** \| **between D0 to D15** \| \| --- \| --- \| --- \| \| Information \| x \|  \| \| Collection of non-opposition \| x \|  \| \| Collection of authorization of right to voice \| x \|  \| \| Collection of contact details (email and telephone) \| x \|  \| \| Collection of medical file data \| x \|  \| \| Collection of elements specified by the doctor \| x \|  \| \| Carrying out the interview \|  \| x \| \| Collection of patient data \|  \| x \| | \| **Actions** \| **J0** \| \| --- \| --- \| \| Information \| x \| \| Collection of non-opposition \| x \| \| Collection of medical file data \| x \| \| Collection of patient data:  rPATD questionnaire, BMQ and other patient questions \| x \| \| Collection of elements specified by the doctor \| x \| \| Literacy Questionnaire Collection (ancillary study) \| x \| |

**Overall 3-year calendar**


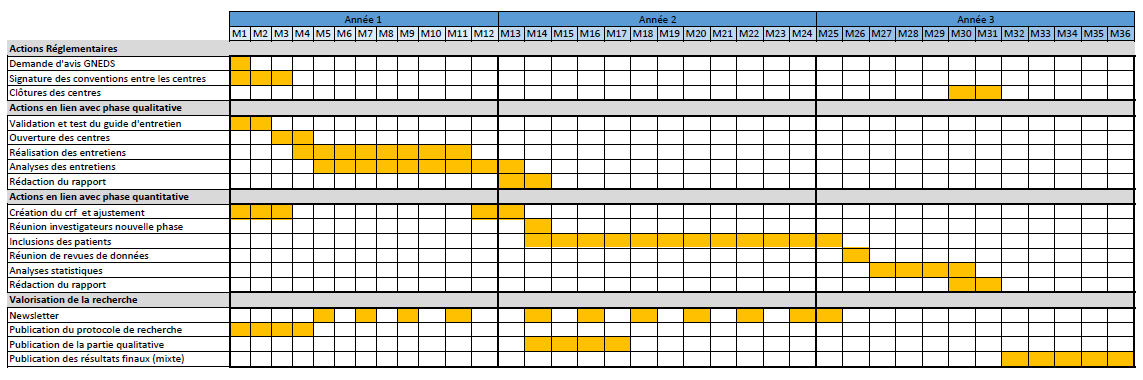


**Schedule Feasibility**

The feasibility of the study schedule was studied by the scientific committee in order to be consistent with the objectives. The project coordinator investigator has 20% dedicated time for this project. The scientific committee is multidisciplinary (one of the strengths of this project) and is experienced in clinical research (two of them work for the research departments of their establishment). He is united and part of the same INSERM research team. All the members of this committee are academics and publish very regularly in international indexed journals. This project will also be supported by work from master 2 students (the members of the scientific committee are teachers in several masters). To have a sufficient number of subjects to include, the choice was made for a multicenter study. The selection of centers was made based on the links that exist between them. This will enable a strong dynamic of inclusion (monthly staff in place for 2 years between the centers). They are all near Nantes and, for the two largest centers, members of the scientific committee work there regularly. In order to ensure the dynamics and rigorous monitoring of inclusions, a clinical study coordinator (clinical research nurse accustomed to end-of-life research) will be involved. Finally, medical time and clinical research technician time are also budgeted to enable quality data entry and to ensure that the centers maintain a rate of inclusion in line with the objectives of the study.

# Administrative and regulatory aspects

We are in a study in human and social sciences in the field of health, observational without risk for the patient. We are therefore outside the Jardé law. Before starting the study, we will seek the opinion of the local Nantais ethics committee (GNEDS Groupe Nantais d'Éthique dans le Domaine de la Santé).

Within the framework of current regulations, the investigator undertakes to inform the patient clearly and fairly of the protocol (written information note of non-opposition to the research in addition to oral information). He will give the patient a copy of the information note. This will specify the possibility for the patient to refuse to participate in the research and to withdraw at any time.

In view of the feedback concerning the use in newsletters of previous studies of the term “patients in a palliative situation”, the scientific committee decided to use the term “patient living with advanced cancer”. Indeed, the term “palliative” is still poorly understood among the population today and is synonymous with imminent end of life, which is not the case with the term “advanced cancer”. This choice aims to prevent the patient from misunderstanding their vital prognosis through this information letter or this study.

Authorization for the right to voice will be collected from patients who will participate in the qualitative part in order to be able to record the semi-directed interviews.

The patient's written agreement to be contacted by a doctor from the Nantes University Hospital will also be collected.

The investigator will note in the patient's file that the patient was informed orally, received the information note and gave written consent to participate in the research as well as for the use of their data for research; he will date this information.

The processing of data will be recorded in the GDPR register of the Nantes University Hospital.

The data collected during the study will be kept in a computer file respecting the law “information technology and freedoms” of January 6, 1978 as amended, law no. 2018-493 of June 20, 2018 relating to the protection of personal data and the Regulation (EU) 2016/679 of the European Parliament and of the Council of April 27, 2016 relating to the protection of individuals with regard to the processing of personal data and the free movement of such data (GDPR). They will therefore be pseudo -anonymized with coding that complies with regulations.

The protocol falls within the scope of the MR004 Reference Methodology to which the Nantes University Hospital, which has authorization from the CNIL, complies.

# expected scientific and public health benefits

- The results of this study will make it possible to **improve knowledge** in the field of the proper use of medications and more particularly the **fight against polypharmacy** through deprescription. The perception of deprescribing among patients in a palliative oncological situation will be better understood, in order to be **able to best adapt the tools but also the discourse/positioning of caregivers on deprescribing** . The objective is to **ultimately reduce polypharmacy in this population, thus improving the quality of life of patients and reducing the costs of care.** (10) .

- The study will provide a better understanding of the links/associations that may exist between patients' perceptions of deprescribing and medical, socio-demographic or even literacy elements. In this way, **caregivers will be able to best adapt to the specific elements of the patient.**

- The ancillary study on the health literacy of this population will allow us to better **understand both the level of literacy of our patients and to think about the actions to be implemented to potentially improve it** . As a reminder, in public health today “improving the level of literacy is a major public health issue so that the population is able to take charge of their health as best as possible. (…) A significant research effort at the international level must be undertaken to raise the level of literacy of as many people as possible.”( [https://www.santepubliquefrance.fr/docs/la-litteratie-en-sante-un-concept -criticism-for-public-health](https://www.santepubliquefrance.fr/docs/la-litteratie-en-sante-un-concept-critique-pour-la-sante-publique) )

- Indirectly, this study will also **probably modify the practices of centers by raising doctors' awareness of the issue of PIMs and deprescribing in palliative care** . Training **for healthcare professionals** could be organized based on our results on the themes of polypharmacy and deprescribing in palliative care.

- This study will also be the start of a project, which will be continued afterwards by **the implementation of deprescribing actions adapted to this population (by evaluating in a more specific manner the different areas of quality of life of potentially impacted patients, the health costs…** ).

List of annexes

- ANNEX 1: Bibliographic references
- APPENDIX 2: List of main speakers
- APPENDIX 3: Questionnaires
- APPENDIX 4: Patient information note and authorization of voice and image rights
- ANNEX 5: Commitment to compliance with MR004

ANNEX 1: BIBLIOGRAPHICAL REFERENCES

1. WHO Center for Health Development (Kobe J. A glossary of terms for community health care and services for older persons [Internet]. Kobe, Japan: WHO Center for Health Development; 2004 [cited 26 Apr 2023]. Report No.: WHO/WKC/Tech.Ser./04.2. Available at: https://apps.who.int/iris/handle/10665/68896

2. Scott IA, Hilmer SN, Reeve E, Potter K, Le Couteur D, Rigby D, et al. Reducing inappropriate polypharmacy: the process of deprescribing. JAMA Intern Med. May 2015;175(5):827 - 34.

3. Cadogan CA, Murphy M, Boland M, Bennett K, McLean S, Hughes C. Prescribing practices, patterns, and potential harms in patients receiving palliative care: A systematic scoping review. Explor Res Clin Soc Pharm. Sep 2021;3:100050.

4. Schenker Y, Park SY, Jeong K, Pruskowski J, Kavalieratos D, Resick J, et al. Associations Between Polypharmacy, Symptom Burden, and Quality of Life in Patients with Advanced, Life-Limiting Illness. J Gen Intern Med. Apr 2019;34(4):559 - 66.

5. Reeve E, Gnjidic D, Long J, Hilmer S. A systematic review of the emerging definition of “deprescribing” with network analysis: implications for future research and clinical practice. Br J Clin Pharmacol. Dec 2015;80(6):1254 - 68.

6. Morin L, Wastesson JW, Laroche ML, Fastbom J, Johnell K. How many older adults receive drugs of questionable clinical benefit near the end of life? A cohort study. Palliat Med. Sep 2019;33(8):1080 - 90.

7. Lees J, Chan A. Polypharmacy in elderly patients with cancer: clinical implications and management. Lancet Oncol. Dec 2011;12(13):1249 - 57.

8. Todd A, Al-Khafaji J, Akhter N, Kasim A, Quibell R, Merriman K, et al. Missed opportunities: unnecessary medicine use in patients with lung cancer at the end of life - an international cohort study. Br J Clin Pharmacol. Dec 2018;84(12):2802 - 10.

9. Blower P, de Wit R, Goodin S, Aapro M. Drug–drug interactions in oncology: Why are they important and can they be minimized? Crit Rev Oncol Hematol. 2005 Aug 1 [cited 2023 Apr 26];55(2):117 – 42.

10. Kutner JS, Blatchford PJ, Taylor DH, Ritchie CS, Bull JH, Fairclough DL, et al. Safety and Benefit of Discontinuing Statin Therapy in the Setting of Advanced, Life-Limiting Illness. JAMA Intern Med. May 2015;175(5):691 - 700.

11. Meyer-Junco L. Time to Deprescribe: A Time-Centric Model for Deprescribing at End of Life. J Palliat Med. Feb 2021;24(2):273 - 84.

12. Tjia J, Karakida M, Alcusky M, Furuno JP. Perspectives on deprescribing in palliative care. Expert Rev Clin Pharmacol. Apr 19, 2023;1 – 11.

13. Paque K, Vander Stichele R, Elseviers M, Pardon K, Dilles T, Deliens L, et al. Barriers and enablers to deprescribing in people with a life-limiting disease: A systematic review. Palliat Med. Jan 2019;33(1):37 - 48.

14. Harris D. Safe and effective prescribing for symptom management in palliative care. Br J Hosp Med Lond Engl 2005. 2 Dec 2019;80(12):C184 - 9.

15. Van Merendonk LN, Crul M. Deprescribing in palliative patients with cancer: a concise review of tools and guidelines. Support Care Cancer Off J Multinatl Assoc Support Care Cancer. Apr 2022;30(4):2933 - 43.

16. Tjia J, Kutner JS, Ritchie CS, Blatchford PJ, Bennett Kendrick RE, Prince-Paul M, et al. Perceptions of Statin Discontinuation among Patients with Life-Limiting Illness. J Palliat Med. Oct 2017;20(10):1098 - 103.

17. Todd A, Holmes H, Pearson S, Hughes C, Andrew I, Baker L, et al. “I don't think I'd be frightened if the statins went”: a phenomenological qualitative study exploring medicines use in palliative care patients, carers and healthcare professionals. BMC Palliat Care. Jan 29, 2016; 3:13 p.m.

18. Sørensen K, Van den Broucke S, Fullam J, Doyle G, Pelikan J, Slonska Z, et al. Health literacy and public health: a systematic review and integration of definitions and models. BMC Public Health. Jan 25, 2012;12:80 p.m.

19. Palliative care. WHO [Internet]. [cited April 26, 2023]. Available at: https://www.who.int/en/news-room/fact-sheets/detail/palliative-care

20. Curtin D, Gallagher P, O'Mahony D. Deprescribing in older people approaching end-of-life: development and validation of STOPPFrail version 2. Age Aging. Feb 26, 2021;50(2):465 - 71.

21. McNeill R, Hanger HC, Chieng J, Chin P. Polypharmacy in Palliative Care: Two Deprescribing Tools Compared with a Clinical Review. J Palliat Med. May 2021;24(5):661 - 7.

22. Hui D, Paiva CE, Del Fabbro EG, Steer C, Naberhuis J, van de Wetering M, et al. Prognosis in advanced cancer: update and directions for future research. Support Care Cancer Off J Multinatl Assoc Support Care Cancer. June 2019;27(6):1973 - 84.

23. White N, Kupeli N, Vickerstaff V, Stone P. How accurate is the “Surprise Question” at identifying patients at the end of life? A systematic review and meta-analysis. BMC Med. 2 Aug 2017;15(1):139.

24. White N, Reid F, Harris A, Harries P, Stone P. A Systematic Review of Predictions of Survival in Palliative Care: How Accurate Are Clinicians and Who Are the Experts? PloS One. 2016;11(8):e0161407.

25. Fortin MF, Gagnon J. Foundations and stages of the research process. 4th edition. CHENELIERE; 2022. 496 p.

26. Sandelowski M. Whatever happened to qualitative description? Res Nurs Health. August 2000;23(4):334 - 40.

27. Sandelowski M. What's in a name? Qualitative description revisited. Res Nurs Health. Feb 2010;33(1):77 - 84.

28. Braun V, Clarke V. Using thematic analysis in psychology. Qual Res Psychol. 1 Jan 2006;3(2):77 - 101.

29. Braun V, Clarke V. Reflecting on reflexive thematic analysis. Qual Res Sport Exercise Health. August 8, 2019;11(4):589 - 97.

30. Braun V, Clarke V. Conceptual and design thinking for thematic analysis. Qual Psychol. 2022;9:3 - 26.

31. Tong A, Sainsbury P, Craig J. Consolidated criteria for reporting qualitative research (COREQ): a 32-item checklist for interviews and focus groups. Int J Qual Health Care J Int Soc Qual Health Care. Dec 2007;19(6):349 - 57.

32. Braun V, Clarke V. One size fits all? What counts as quality practice in (reflexive) thematic analysis? Qual Res Psychol. Jul 3, 2021;18(3):328 - 52.

33. Braun V, Clarke V. To saturate or not to saturate? Questioning data saturation as a useful concept for thematic analysis and sample-size rationales. Qual Res Sport Exercise Health. March 4 202;113(2):201 - 16.

34. Oken MM, Creech RH, Tormey DC, Horton J, Davis TE, McFadden ET, et al. Toxicity and response criteria of the Eastern Cooperative Oncology Group. Am J Clin Oncol. Dec 1982;5(6):649 - 55.

35. Fall E, Gauchet A, Izaute M, Horne R, Chakroun N. Validation of the French version of the Beliefs about Medicines Questionnaire (BMQ) among diabetes and HIV patients. Eur Rev Appl Psychol. 1 Nov 2014;64(6):335 - 43.

36. Roux B, Sirois C, Niquille A, Spinewine A, Ouellet N, Pétein C, et al. Cross-cultural adaptation and psychometric validation of the revised Patients' Attitudes Towards Deprescribing (rPATD) questionnaire in French. Res Soc Adm Pharm RSAP. August 2021;17(8):1453 - 62.

37. Gaurang N, Priyadharsini R, Balamurugesan K, Prakash M, Reka D. Attitudes and beliefs of patients and primary caregivers towards deprescribing in a tertiary health care facility. Pharm Pract. 2021;19(3):2350.

38. Lukacena KM, Keck JW, Freeman PR, Harrington NG, Huffmyer MJ, Moga DC. Patients' attitudes toward deprescribing and their experiences communicating with clinicians and pharmacists. Ther Adv Drug Saf. 2022;13:20420986221116464.

39. Nguyen-Soenen J, Rat C, Gaultier A, Schirr-Bonnans S, Tessier P, Fournier JP. Effectiveness of a multi-faceted intervention to deprescribe proton pump inhibitors in primary care: protocol for a population-based, pragmatic, cluster-randomized controlled trial. BMC Health Serv Res. Feb 17, 2022;22(1):219.

40. Ousseine YM, Rouquette A, Bouhnik AD, Rigal L, Ringa V, Smith A “Ben”, et al. Validation of the French version of the Functional, Communicative and Critical Health Literacy scale (FCCHL). J Patient-Rep Outcomes. 2017;2(1):3.

41. Nutbeam D. Health literacy as a public health goal: a challenge for contemporary health education and communication strategies into the 21st century. Health Promot Int. 1 Sep 2000;15(3):259 - 67.

42. Crutzen S, Abou J, Smits SE, Baas G, Hugtenburg JG, Heringa M, et al. Older people's attitudes towards deprescribing cardiometabolic medication. BMC Geriatr. June 16, 2021;21(1):366.

43. Roux B, Rakheja B, Sirois C, Niquille A, Pétein C, Ouellet N, et al. Attitudes and beliefs of older adults and caregivers towards deprescribing in French-speaking countries: a multicenter cross-sectional study. Eur J Clin Pharmacol. 1 Oct 2022;78(10):1633 - 46.

ANNEX 2: LIST OF MAIN SPEAKERS

| **FIRST AND LAST NAME** | **Speciality**  **& Function** | **Facility Name** | **Name of home service** | **Role in the study** |
| --- | --- | --- | --- | --- |
| Adrien EVIN | MCU-PH (palliative medicine) | Nantes University and University Hospital of Nantes | Interdisciplinary Pain Service, Palliative and Supportive Care, Integrative Medicine | Principal investigator |
| André COLPAERT | PH (palliative medicine) | CH CNP and CHU Nantes | Interdisciplinary Pain Service, Palliative and Supportive Care, Integrative Medicine and Mobile Palliative Care Unit CH CNP | Investigator |
| Véronique BARBAROT | Center practitioner (oncologist/palliative medicine) | ICO site Nantes | Medical oncology department | Investigator |
| Yann TOUCHEFEU | PU-PH (gastroenterologist-oncologist) | Nantes University Hospital | multidisciplinary medical oncology unit | Investigator |
| Elvire PONS-TOSTIVIN | MCU-PH | Nantes University Hospital | Thoracic Oncology | Investigator |
| Gaëlle QUEREUX BAUMGARTNER | MCU-PH | Nantes University Hospital | Onco-Dermatology | Investigator |
| Virginie DESSUS-CHEVREL | PH (palliative medicine) | CH ST Nazaire | Palliative care unit | Investigator |
| Caroline HENNION | PH (palliative medicine) | CHD Vendée | Palliative care unit | Investigator |

APPENDIX 3: QUESTIONNAIRES

*
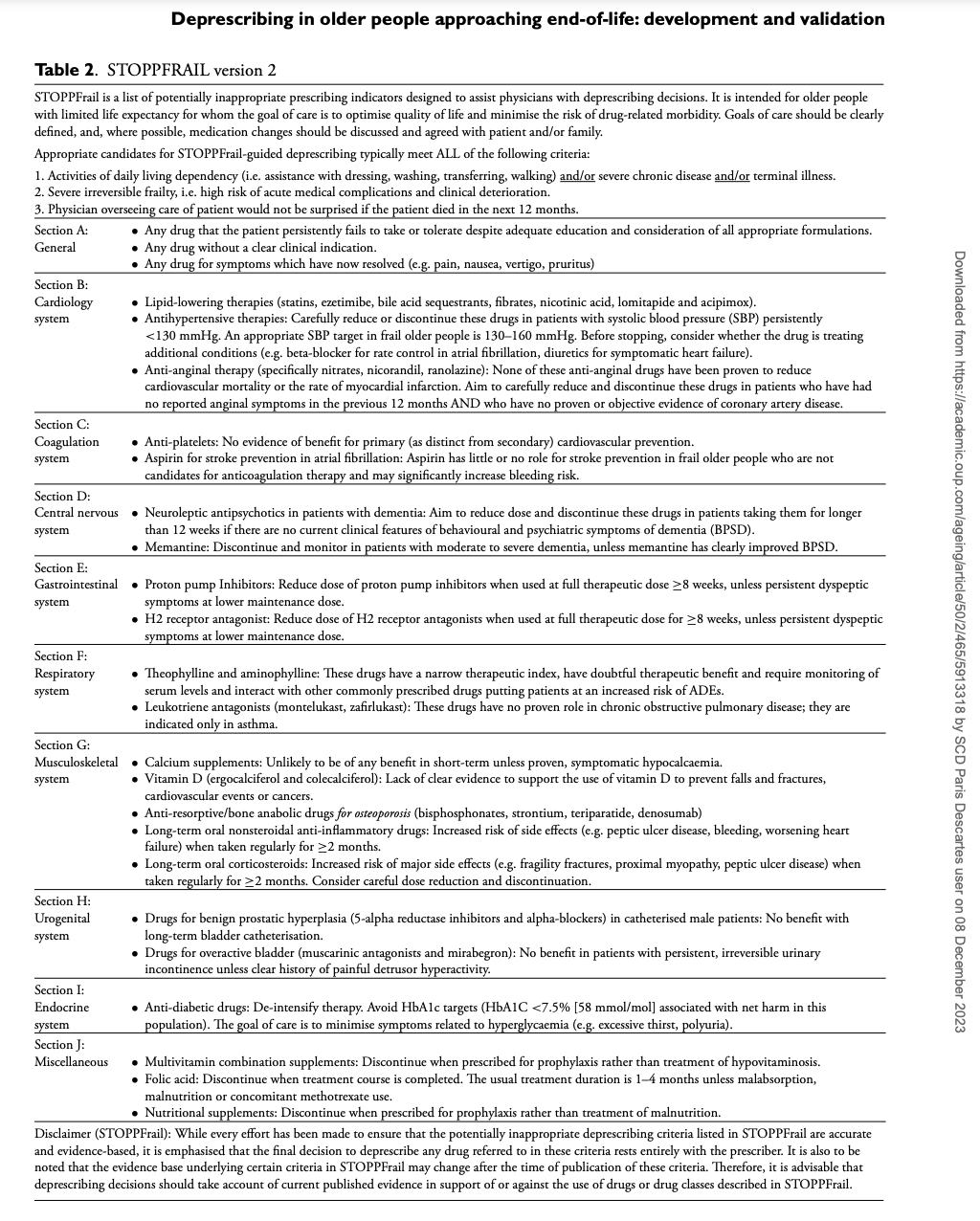
*

***French version of the Beliefs about Medicines Questionnaire.***

We would like to ask you about your personal views regarding the treatments prescribed to you. The following statements have been made by other people regarding their medications.

**• Please indicate to what extent you agree or disagree with them by placing a cross in the appropriate box (answer that best suits you).**

**• There are no right or wrong answers.**

**We are interested in your personal opinions**

| Quite  All right | All right | Uncertain | In  disagreement | Strongly disagree |
| --- | --- | --- | --- | --- |

**Specific beliefs:**

1. My health today depends on my treatment.

2. Having to take medication worries me.

3. My life would be impossible without my treatment.

4. Without my treatment, I would be very sick.

5. I sometimes worry about the long-term effects of my treatment.

6. My treatment is a mystery to me.

7. My future health depends on my treatment.

8. My treatment is disrupting my life.

9. I sometimes worry about becoming too dependent on my treatment.10. My treatment prevents my condition from getting worse.

**General beliefs** :

11. Doctors use too many treatments.

12. People who are taking medications should stop their medication from time to time.

13. Most treatments are addictive.

14. Natural remedies are safer than medical treatments.

15. Treatments do more harm than good.

16. All treatments are poisons.

17. Doctors place too much faith in treatments.

18. If doctors spent more time with patients, they would prescribe fewer treatments.

***rPATD French version***

• Please indicate to what extent you agree or disagree with these propositions by putting a cross in the appropriate box (answer that best suits you).

• There are no right or wrong answers.

| Quite  All right | All right | Uncertain | In  disagreement | Strongly disagree |
| --- | --- | --- | --- | --- |

| Q1 | I spend a lot of money on my medicine |
| --- | --- |
| Q2 | Taking my medication every day is not very practical |
| Q3 | I find that I take a lot of medications |
| Q4 | I find that my medications are a constraint for me |
| Q5 | Sometimes I think I'm taking too much medication |
| Q6 | I find that I may be taking one or more medications that I no longer need |
| Q7 | I would like to try stopping one of my medications to see how I would feel without it. |
| Q8 | I would like my doctor to reduce the dose of one or more of my medications |
| Q9 | I think one or more of my medications may not be working |
| Q10 | I think one or more of my medications may be giving me side effects at the moment |
| Q11 | I would be reluctant to stop a medication that I have been taking for a long time |
| Q12 | If one of my medications was stopped, I would be worried about missing out on its future benefits |
| Q13 | I am worried/stressed every time my medications are changed |
| Q14 | If my doctor recommended that I stop taking a medication, I would feel like he was giving up on treating me. |
| Q15 | I already had a bad experience when a medication was stopped |
| Q16 | I understand why I was prescribed each of my medications |
| Q17 | I know exactly what medications I am currently taking and/or I maintain a medication list |
| Q18 | I like to know as much as possible about my medications |
| Q19 | I like to be involved with my doctors in the decisions that are made regarding my medications |
| Q20 | I always ask the doctor, pharmacist, or other healthcare professional if there is anything I don't understand about my medications |
| Q21 | If my doctor said it was possible, I would be willing to stop one or more of my usual medications |
| Q22 | Overall, I am satisfied with my current medications |

***FCCHL (FUNCTIONAL, COMMUNICATIVE AND CRITICAL HEALTH LITERACY) / HLS14 (14-ITEM HEALTH LITERACY SCALE)***


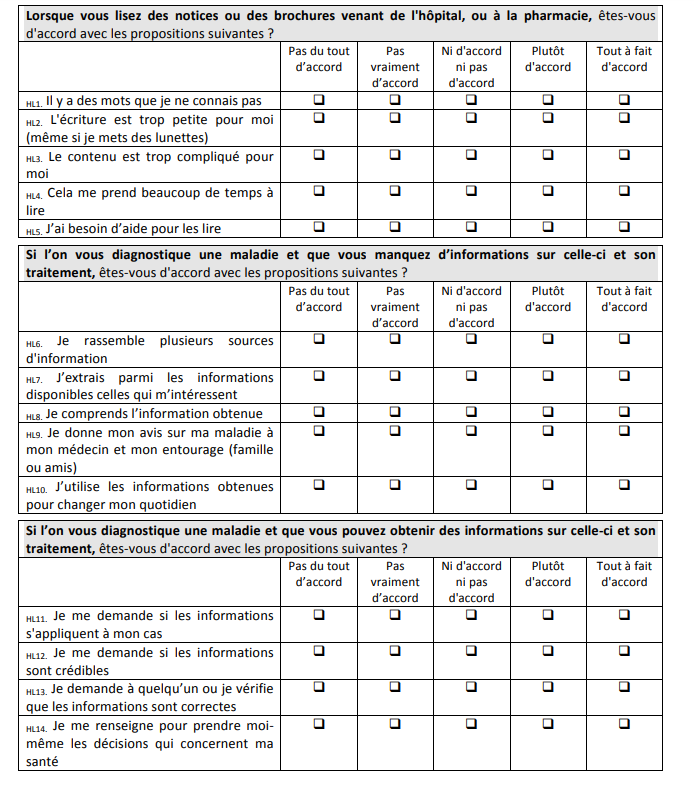


ANNEX 4: PATIENT INFORMATION NOTES


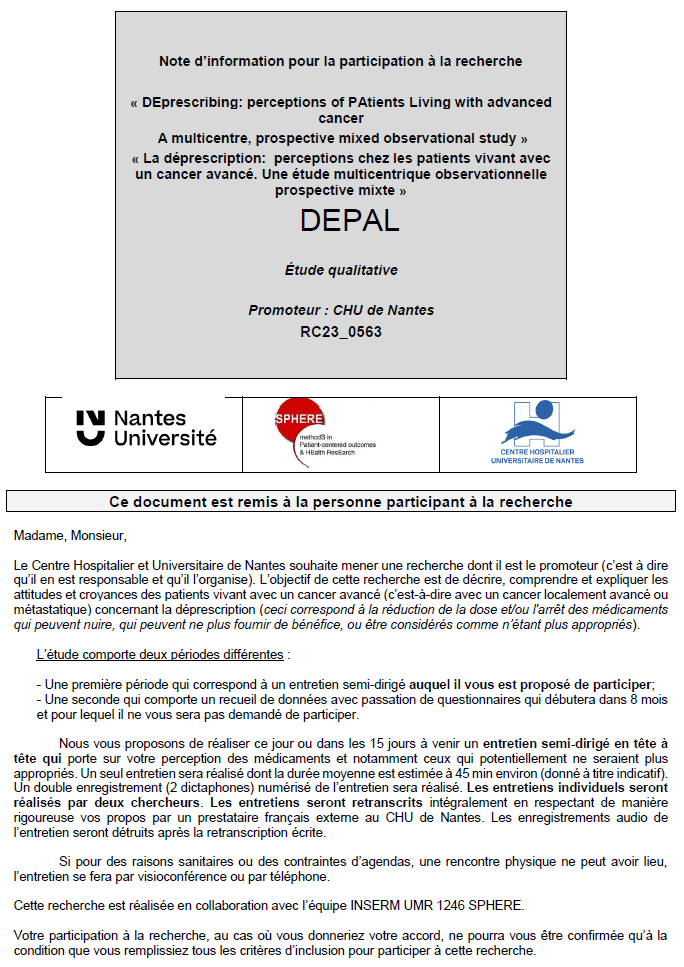


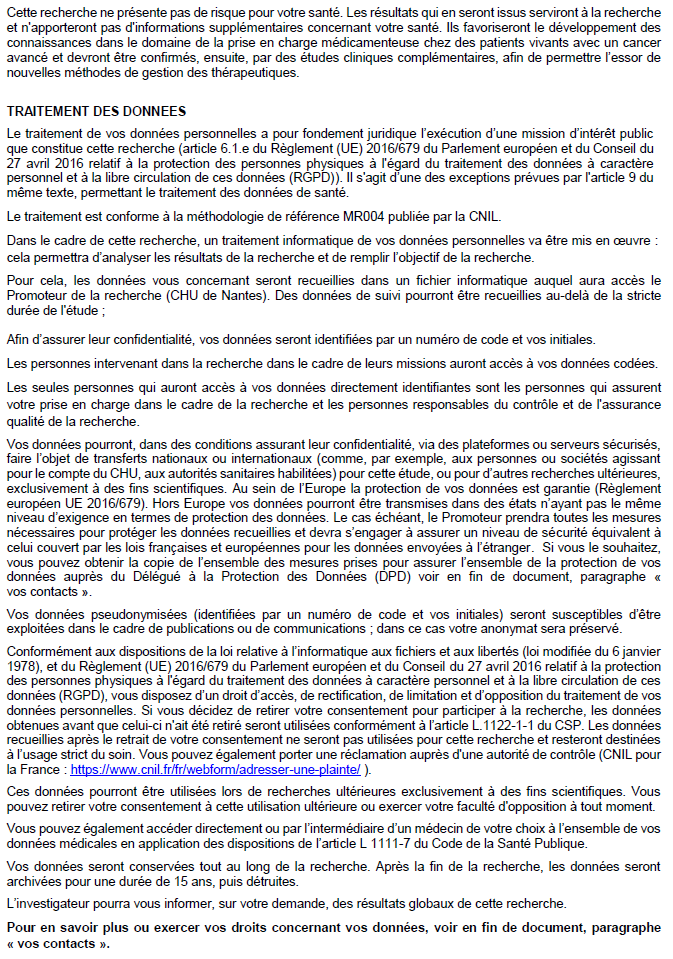


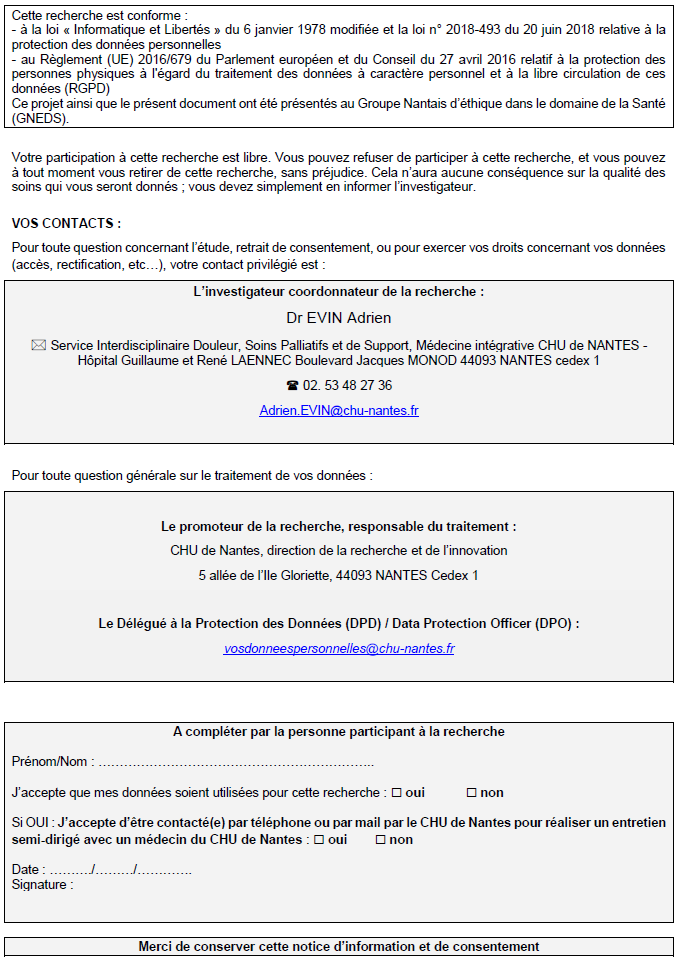


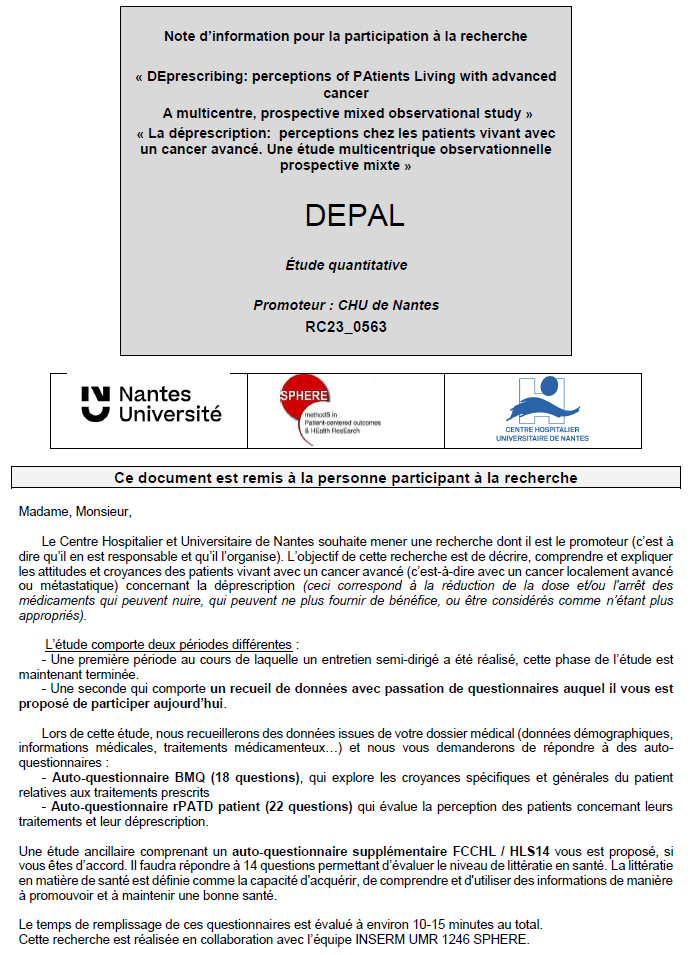


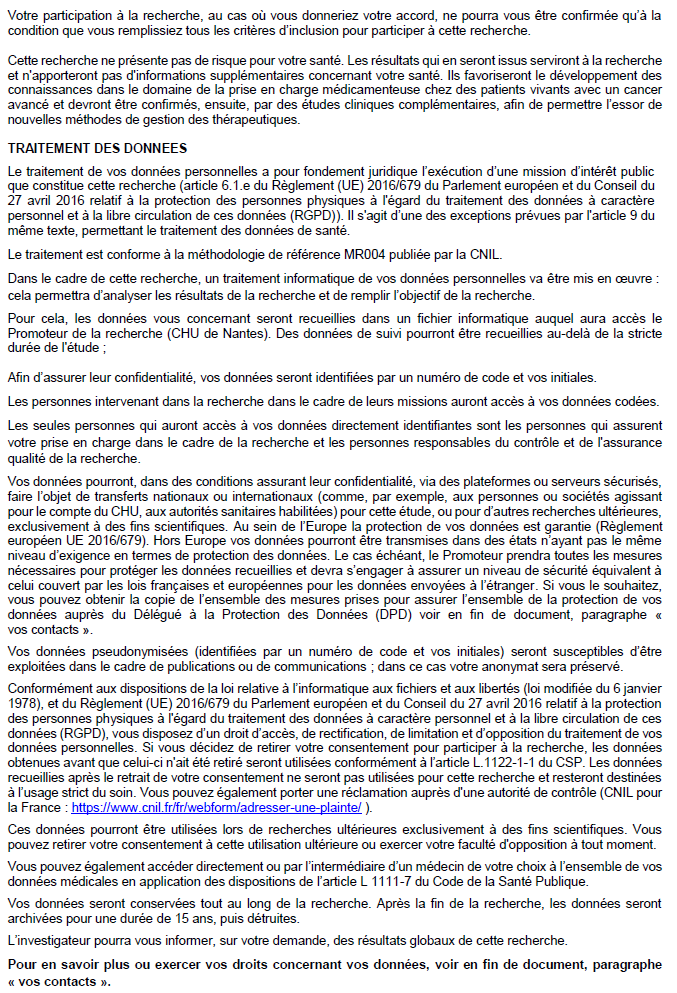


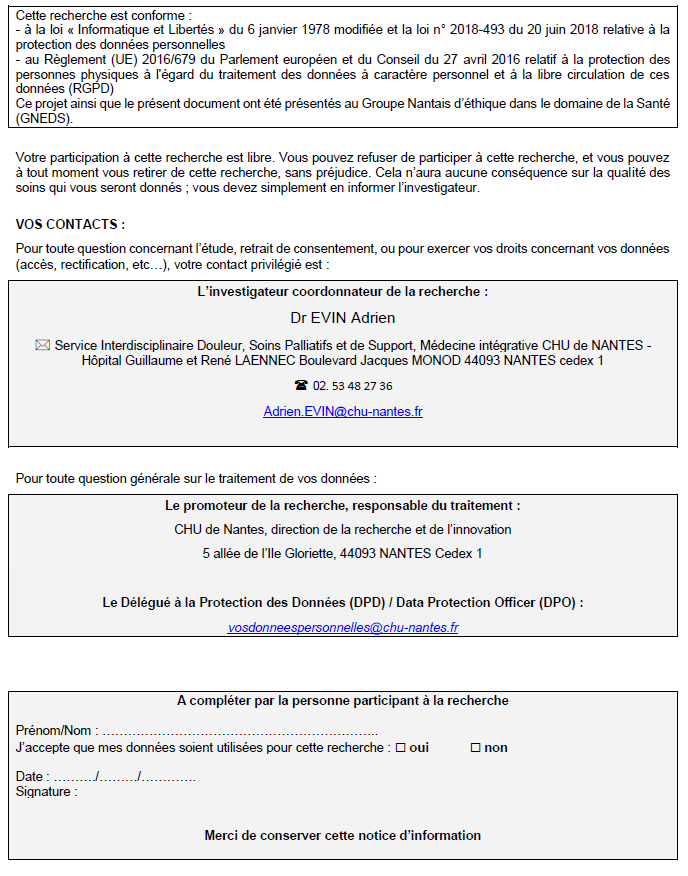


ANNEX 5 : AUTHORIZATION OF RIGHT TO VOICE


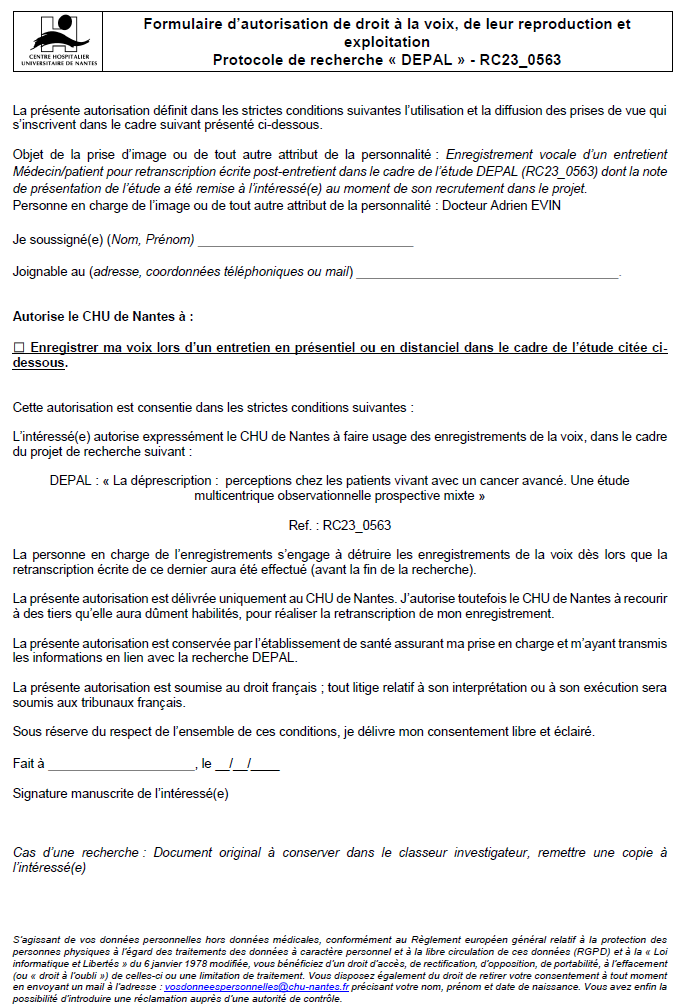


ANNEX 6: Commitment to compliance with MR004


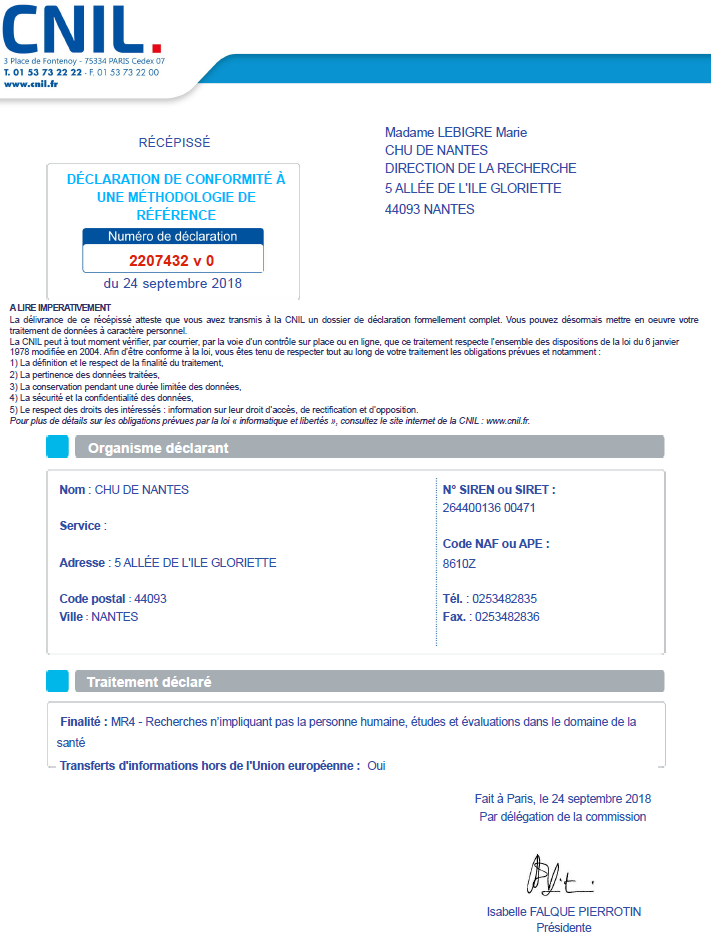

Supplement: S9 File — (DOCX) [file pone.0305737.s010.docx]
